# *Fucus vesiculosus* fucoidan alone and in combination with simvastatin is associated with both alleviation of atherosclerosis and modulations in the gut microbiota and its metabolites in New Zealand rabbits

**DOI:** 10.3389/fmicb.2026.1768989

**Published:** 2026-06-18

**Authors:** Feng-Yu Liu, Zhi-Zhen Liu, Lei Fang, Jing-Jing Huang, Xu-Hang Zhang, Xue-Ying Zhang, Chao-Nan Ma, Shan-Rui Shi, Hong-Hai Ji, Jun-Wei Xin, Shou-Dong Guo

**Affiliations:** 1 Institute of Lipid Metabolism and Atherosclerosis, School of Pharmacy, Shandong Second Medical University, Weifang, China; 2 School of Stomatology, Shandong Second Medical University, Weifang, China; 3 Department of Dentistry, Affiliated Hospital of Shandong Second Medical University, Weifang, China; 4 School of Public Health, Shandong Second Medical University, Weifang, China

**Keywords:** animal model, cardiovascular disease, fucoidan, gut metabolites, gut microbiota, statin

## Abstract

**Background:**

While fucoidans show anti-atherosclerotic potential, their impact on this gut-host axis is unclear. This study investigated the effects of *Fucus vesiculosus* fucoidan, alone and combined with simvastatin, on gut microbiota and metabolome in an atherosclerotic rabbit model that closely recapitulates key features of human atherosclerotic pathogenesis.

**Methods:**

Atherosclerosis was induced in New Zealand rabbits via high-fat diet feeding combined with balloon catheter injury. Animals in the experimental groups (*n* = 6 per group) were treated with either *F. vesiculosus* fucoidan alone (100 mg/kg) or a combination of fucoidan (100 mg/kg) and simvastatin (5 mg/kg). Lipid profiles were assayed using commercial kits, and aortic pathological changes were evaluated by Oil Red O staining. Genomic DNA from intestinal contents was analyzed by polymerase chain reaction, and untargeted metabolomics were performed using liquid chromatography–tandem mass spectrometry.

**Results:**

Treatments significantly reduced atherosclerotic plaque formation. This effect was particularly pronounced when fucoidan was combined with simvastatin, reducing plaque formation from 41.77 ± 16.02% to 5.91 ± 8.03% in the abdominal aorta and from 10.72 ± 3.49% to 2.29 ± 2.30% in the thoracic aorta (Model vs. combination group). The treatment also ameliorated hyperlipidemia, as shown by decreased plasma TC (24.55 ± 0.73 to 17.45 ± 0.58 mmol/L) and TG (7.75 ± 0.46 to 0.83 ± 0.25 mmol/L) in the combination group compared to the Model group. Both intervention groups exhibited enhanced microbial diversity and increased species richness across all taxonomic levels compared to the model group. Moreover, fucoidan and combination treatments significantly upregulated 83 and 128 metabolites and downregulated 125 and 121 metabolites, respectively. KEGG enrichment analysis indicated that these metabolic changes were associated with multiple pathways. Moreover, the combination therapy may mitigate certain side effects associated with simvastatin, although this possibility requires further investigation.

**Conclusion:**

The combination of *F. vesiculosus* fucoidan and simvastatin holds promising therapeutic potential for preventing and treating atherosclerosis. Our findings provide novel evidence that this enhanced effect is likely linked to structural and functional modifications in the gut microbiota and its associated metabolic profile, supporting a multifaceted strategy that extends beyond lipid-lowering.

## 1 Introduction

Cardiovascular diseases (CVDs) affect individuals across different age groups, imposing a significant societal burden, with atherosclerosis being the primary underlying cause of most CVDs. Accumulating evidence indicates that dyslipidemia—particularly elevated total cholesterol (TC) and triglyceride (TG) levels—plays a central role in atherosclerosis pathogenesis (Zhang et al., 2022; Lee et al., 2025). While statins, the first-line hypolipidemic drugs, effectively lower cholesterol by inhibiting HMG-CoA reductase, they cannot fully suppress atherosclerosis progression and are associated with various adverse effects (Lamminpää et al., 2024; Brandts and Müller-Wieland, 2025).

The gut microbiome plays a pivotal role in human health and disease, earning its designation as the “tenth system” of the human body. It evolves dynamically with the host—developing, maturing, and aging—while producing diverse bioactive metabolites that enter systemic circulation to exert widespread physiological effects (Shi et al., 2024; Adolph and Tilg, 2024). Critically, gut microbiota dysbiosis has been linked not only to gastrointestinal pathologies but also to host metabolic disturbances in lipid and glucose homeostasis, underscoring its regulatory influence on CVDs (Lamminpää et al., 2024; Adolph and Tilg, 2024; Warmbrunn et al., 2024). Preclinical studies using apolipoprotein E-deficient mice and other models demonstrate that high-fat diet (HFD) reshapes gut microbial communities and their metabolic outputs—including lipids, amino acids, trimethylamine N-oxide (TMAO), and short-chain fatty acids—which collectively modulate lipid disorders, atherogenesis, and thrombotic risk (Lamminpää et al., 2024; Xue et al., 2025; Khuu et al., 2025). These clinical and experimental findings position the gut microbiota as a promising target for novel therapeutic strategies, precision diagnostics, and personalized CVD interventions (Shi et al., 2024).

Marine-derived bioactive compounds have emerged as promising modulators of CVD risk factors through their ability to influence gut microbiota composition and function (Lamminpää et al., 2024; Wan et al., 2025; Yang et al., 2022). Particularly noteworthy are brown algae-derived fucoidans, which exhibit multi-faceted anti-atherosclerotic properties by simultaneously addressing hyperlipidemia, oxidative stress, and inflammation across various animal models (Lamminpää et al., 2024; Liu T. et al., 2024; Łagowska et al., 2025). These effects are partially mediated through gut microbial fermentation of dietary fibers like fucoidan and fructan, which reshape the intestinal microbial ecosystem and its metabolic output, ultimately improving inflammatory markers, lipid homeostasis, and oxidative stress parameters, key determinants of cardiovascular health in experimental models (Delzenne et al., 2025; du Preez et al., 2021; Liu et al., 2022; Wang et al., 2020; Zhong et al., 2024).

Despite growing evidence of fucoidans’ cardiovascular benefits, their precise mechanisms of action, particularly concerning the gut-heart axis, remain poorly understood. Current mechanistic insights primarily derive from rodent studies, which are limited by significant metabolic differences from humans. In contrast to rodents, rabbits offer several advantages for atherosclerosis research. First, they express cholesteryl ester transfer protein and exhibit high plasma levels of low-density lipoprotein cholesterol—a lipid profile that closely mirrors that of humans. Second, they uniquely modify apoB100 mRNA expression, further aligning their lipid metabolism with the human condition. Third, Rabbits possess a sufficiently large aorta, making them well suited for balloon catheter injury procedures. Following injury, rabbits subsequently develop robust neointimal hyperplasia, marked by extensive lipid infiltration, a high density of smooth muscle cells, and the accumulation of macrophage-derived foam cells—features that closely resemble those of human atherosclerotic plaques (Jain et al., 2017; Kamato et al., 2022). These features make rabbits particularly appropriate for atheroma formation studies. Importantly, because rabbit lipid profiles closely resemble those of humans, findings from rabbit models have greater potential for clinical translation. Therefore, rabbit models provide distinct advantages for atherosclerosis research, including enhanced physiological relevance for evaluating anti-atherosclerotic compounds and their mechanisms, as well as facilitating potential clinical applications (Hayashi et al., 2023; Kamato et al., 2022). Our previous studies (Shi et al., 2025; Wang et al., 2025) demonstrated that the combination of *Fucus vesiculosus*-derived fucoidan and simvastatin effectively attenuated atherosclerosis in a rabbit model, exhibiting effects on lipid regulation and inflammatory pathways, such as reductions in pro-inflammatory factors, while confirming safety, as indicated by unchanged alanine aminotransferase and aspartate aminotransferase levels. However, the specific and detailed alterations in gut microbiota and systemic metabolome, which are crucial for understanding the underlying mechanisms, remain unexplored. Furthermore, the potential of fucoidan to modulate statin-associated side effects via the gut microbiome represents a novel and clinically significant question.

**Figure 1:**
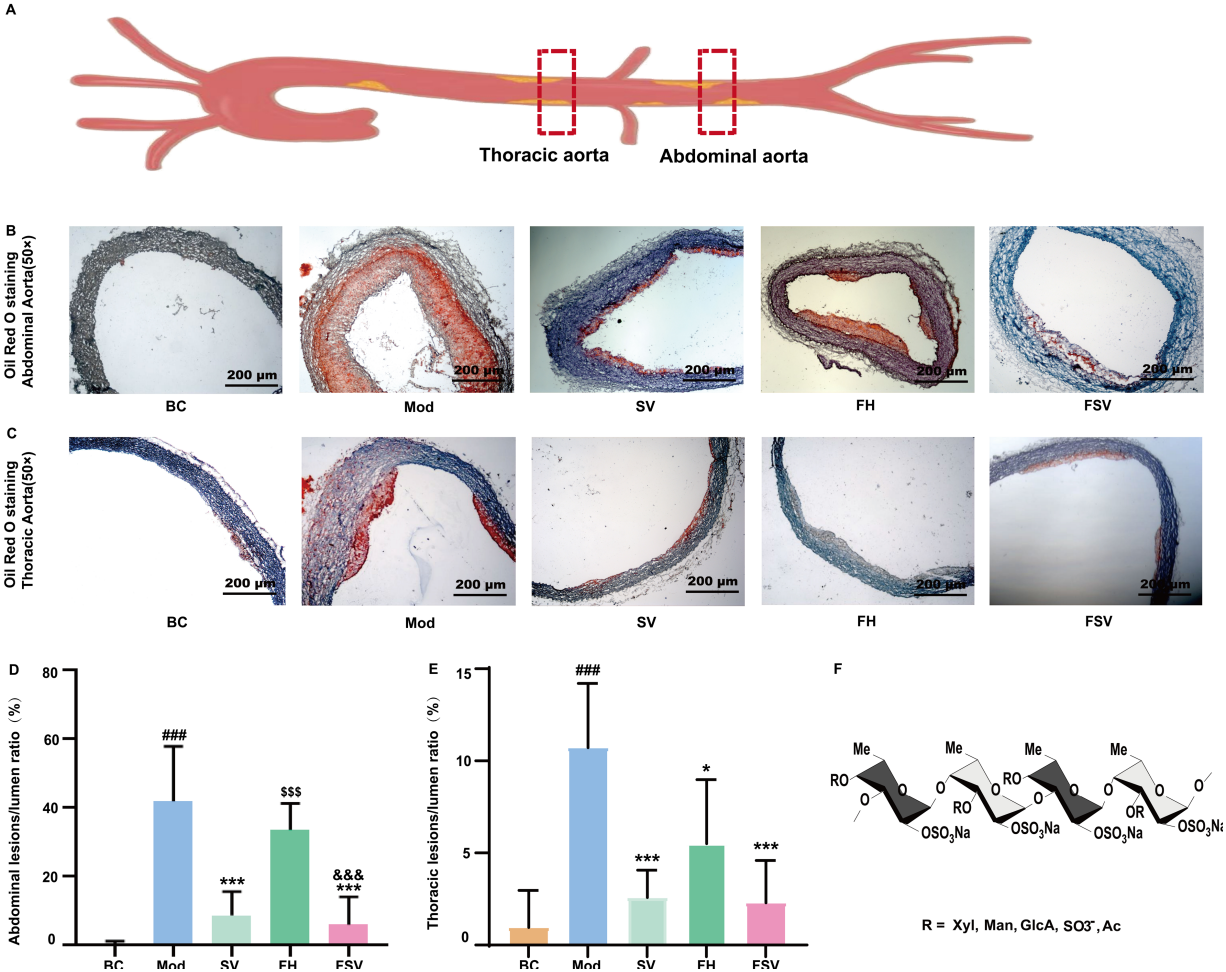
Effects of *F. vesiculosus*-derived fucoidan alone or in combination with simvastatin on atherosclerotic plaque formation in New Zealand rabbits (*n* = 6). **(A)** Illustrates the schematic diagram for the preparation of aorta sections; **(B)** displays representative images of abdominal aorta sections stained with Oil Red O and hematoxylin; **(C)** shows typical images of thoracic aorta sections stained with Oil Red O and hematoxylin; **(D)** presents the statistical analysis of Oil Red O-staining areas in the abdominal aorta; **(E)** provides the corresponding quantitative analysis for the thoracic aorta sections; and **(F)** shows the presumed structure of *F. vesiculosus*-derived fucoidan. BC: Rabbits fed a chow diet; Mod: rabbits fed an HFD; SV: rabbits fed an HFD and treated with simvastatin (5 mg/kg/d); FH: rabbits fed an HFD and treated with *F. vesiculosus*-derived fucoidan (100 mg/kg/d); FSV: rabbits fed an HFD and treated with simvastatin (5 mg/kg/d) and *F. vesiculosus*-derived fucoidan (100 mg/kg/d). ^###^*p* < 0.001 *vs.* BC group; ^*^p < 0.05 *vs.* Mod group; ^***^p < 0.001 *vs.* Mod group; ^$$$^means *p* < 0.001 vs. SV group; ^&&&^means *p* < 0.001 vs. FH group.

To address these gaps, we investigated the therapeutic potential of *F. vesiculosus* fucoidan (Figure 1F), both alone and in combination with simvastatin, in a New Zealand rabbit model of atherosclerosis induced by HFD and balloon catheter injury, with particular focus on gut microbiota modulation and associated metabolic changes. By integrating 16S rRNA sequencing and untargeted metabolomics, we sought to elucidate the microbial and metabolic mechanisms underlying the observed therapeutic benefits, thereby providing deeper mechanistic insights into our earlier findings.

## 2 Materials and methods

### 2.1 Materials

*Fucus vesiculosus* fucoidan (FvF), provided by Weihai Rensheng Pharmaceutical Group Co., Ltd. (Weihai, China), was further purified by our group using a previously reported method (Shi et al., 2025). The resulting FvF had a molecular weight of ~517.6 kDa, contained 20.7% sulfate and 73.5% carbohydrate, and was composed primarily of fucose (64.5%), xylose (14.5%), and glucuronic acid (5.0%), with trace amounts of galactose and glucose. All pharmaceutical reagents were obtained from commercial sources: propofol medium/long-chain fat emulsion injection from Guangdong Jiabo Pharmaceutical Co., Ltd. (Guangdong, China); iodophor disinfectant from Shandong Lilkang Medical Technology Co., Ltd. (Shandong, China); pentobarbital sodium from Sinopharm Chemical Reagent Co., Ltd. (Beijing, China); heparin sodium from Solarbio Science & Technology Co., Ltd. (Beijing, China); lidocaine hydrochloride injection and normal saline from Shandong Hualu Pharmaceutical Co., Ltd. (Liaocheng, China); and penicillin sodium from Shandong Lukang Pharmaceutical Co., Ltd. (Jining, China). Medical devices included a Merit analog inflation device (Merit Medical Systems, South Jordan, UT, USA) and Gateway PTA balloon catheter (Boston Scientific Corporation, Marlborough, MA, USA). All remaining reagents were of analytical grade.

### 2.2 Animal grouping and treatment

This study was conducted in compliance with ethical standards following approval by the Laboratory Animal Ethical Committee of Shandong Second Medical University (Approval No. 2020SDL106) and adhered to both the ARRIVE guidelines and the National Research Council’s Guide for the Care and Use of Laboratory Animals. Given the potential influence of estrogen on the experimental results, female New Zealand rabbits were excluded from this study (Hayashi et al., 2000). Male New Zealand rabbits (body weight 1.56 ± 0.05 kg) were obtained from Qingdao Kangda Biotechnology Co., Ltd. (Qingdao, China). Balloon catheter-induced endothelial denudation was performed using an established methodology (Jain et al., 2017). Surgical anesthesia was achieved via intravenous injection of 3% pentobarbital sodium solution (1.0 mL/kg) through the marginal ear vein.

Before grouping, an *a priori* power analysis was conducted using SPSSAU online software^1^ with a power of 0.8 and *α* = 0.05 (Langan and Brooks, 2022). Based on the plasma TC and TG values from rabbits on an HFD treated with *F. vesiculosus*-derived fucoidan or simvastatin (Wang et al., 2025), the analysis yielded a minimum sample size of 3 per group. The surviving rabbits that had undergone the operation were randomly allocated into four experimental groups: A model group (Mod), a simvastatin control group (SV, 5 mg/kg), an FvF alone group (FH, 100 mg/kg), and an FvF-simvastatin combination group (FSV, 100 mg/kg + 5 mg/kg). All operated groups received 300–400 g/day of an HFD (provided as 3 separate meals per day) consisting of normal chow supplemented with 10% lard and 0.5% cholesterol. Non-operated rabbits served as blank controls (BC) and were maintained on standard diet *ad libitum*. The detailed methodology for grouping and intervention is available in our previous report (Shi et al., 2025). Adhering to the 3R principles, we utilized a subset of animals from that study by randomly selecting six from each group for the present investigation.

A previous meta-analysis reported that fucoidan dosages in human participants ranged from 350 to 5,000 mg/d (Łagowska et al., 2025). Based on the Meeh-Rubner equation for body surface area conversion (Spiers and Candas, 1984), this range was adjusted to a suitable rabbit dosage of 25–350 mg/d. Accordingly, a fucoidan dosage of 100 mg/kg was selected for this study, consistent with this calculation and previously established protocols (Liu T. et al., 2024; Zheng et al., 2023; Carrasqueira et al., 2025; Ren et al., 2024). Regarding the dosing regimen, the elimination half-life (t_1/2_) of fucoidan in rat plasma after oral administration is approximately 3.4–4.1 h (Pozharitskaya et al., 2018; Zhang et al., 2020). However, the t_1/2_ value may increase to over 10 h in rabbits as discussed in the “Discussion” section. To ensure consistent drug intake, 50 g of medicated HFD (e.g., containing 100 mg/kg fucoidan) was provided as the first meal of the day each morning. A dedicated staff member verified that each rabbit had consumed the entire 50 g of medicated feed before an additional 50 g of non-medicated HFD was supplied. Similarly, the simvastatin dosage of 5 mg/kg used in this study was determined based on the Meeh-Rubner equation and relevant literature (Hernández-Presa et al., 2003; Wang et al., 2025).

### 2.3 Sample collection in experimental animals

Following the final drug administration, rabbits underwent an 8-h fasting period before sample collection. After recording final body weights, animals were anesthetized with isoflurane, and blood samples were obtained via cardiac puncture. Plasma was separated by centrifugation at 1,000 × g for 20 min. Systemic perfusion with 50 mL normal saline was performed prior to tissue collection (Shi et al., 2025). Small intestinal contents were aseptically collected and flash-frozen in liquid nitrogen. Aortic tissues were carefully dissected, with thoracic and abdominal segments embedded in optimal cutting temperature (OCT) compound and stored at −80 °C for subsequent analysis (Figure 1A).

### 2.4 Lipids analysis

Plasma TC and TG concentrations were quantified using commercial enzymatic assay kits (Biosino Bio-technology and Science Inc., Beijing, China) following the manufacturer’s standardized protocols.

### 2.5 Oil red O staining

The Oil Red O staining protocol followed established methodology (Yin et al., 2019) with the following specifications: A stock solution (0.5% w/v) was prepared by dissolving Oil Red O powder in 100 mL isopropanol, with working solution freshly prepared in a 3:2 ratio of stock to distilled water. Aortic sections (7 μm thickness) were processed through sequential steps: (1) air-drying (15 min, room temperature), (2) rapid dehydration in 60% isopropanol (10 s), (3) staining with filtered (0.45 μm) Oil Red O working solution (15 min, room temperature), (4) differentiation in 60% isopropanol, (5) rinsing in distilled water (3 min), and (6) nuclear counterstaining with hematoxylin (30 s). Stained sections were imaged using an Axio Vert. A1 inverted microscope (Zeiss, Jena, Germany).

### 2.6 Gut microbiota analysis

Intestinal content genomic DNA was extracted using the MagPure Soil DNA LQ Kit (Magen, Guangzhou, China) following manufacturer protocols. DNA quality assessment included concentration measurement (NanoDrop 2000 spectrophotometer, Thermo Fisher Scientific, Waltham, MA, USA) and integrity verification (1% agarose gel electrophoresis). PCR amplification was performed using extracted DNA as template with barcoded primers and Tks Gflex DNA Polymerase (Takara Bio Inc., Kusatsu, Japan), targeting V3-V4 hypervariable regions of 16S rRNA genes (primers 343F:5′-TACGGRAGGCAGCAG-3′/798R:5′-AGGGTATCTAATCCT-3′). Amplicons were quality-checked by electrophoresis, purified using Agencourt AMPure XP beads (Beckman Coulter Inc.), and subjected to a second PCR. After additional AMPure XP bead purification and quantification (Qubit dsDNA Assay Kit), equimolar pools were sequenced on an Illumina NovaSeq 6000 platform (2 × 250 bp paired-end; San Diego, CA, USA).

Raw sequencing data in FASTQ format were generated from original image files through base calling. Adapter sequences were trimmed from the raw reads using Cutadapt. Subsequently, quality filtering, denoising, read merging, and chimera removal were performed using the DADA2 plugin within QIIME2 with default parameters, resulting in the generation of amplicon sequence variants (ASVs), which represent unique, dereplicated sequences resolved by the DADA2 denoising algorithm. The abundance of each ASV per sample was quantified by counting the number of quality-filtered tags assigned to that ASV. To ensure reliable comparisons, we confirmed that the sequencing depth—reflected by the number of high-quality reads per sample—was sufficient to capture the majority of microbial diversity, as evidenced by rarefaction curves approaching saturation. To normalize uneven sequencing depths across samples, the ASV table was rarefied by random subsampling without replacement to the ASV count of the sample with the lowest depth, and all subsequent alpha and beta diversity analyses were performed on this rarefied dataset. For differential abundance analysis, cumulative sum scaling normalization was applied as a robust alternative to rarefaction. Alpha diversity indices (e.g., Chao1, Shannon, and Simpson) were calculated to reflect microbial community richness and diversity, whereas beta diversity analyses (e.g., Bray–Curtis, weighted UniFrac, and unweighted UniFrac) were conducted to evaluate differences in microbial composition between samples. Due to the large number of ASVs and the inherently noisy nature of microbiome sequencing data with subtle differences between groups, applying FDR correction often yields few or no statistically significant results—a common issue in studies with limited sample sizes or small intergroup effect sizes. Therefore, to explore potentially differential microbial taxa, we employed uncorrected *p*-values (*p* < 0.05) for preliminary screening in the present study. Furthermore, the linear discriminant analysis effect size (LefSe) analysis was used to screen for statistically significant microbial taxa, with a linear discriminant analysis (LDA) score threshold greater than 2 and a *p*-value less than 0.05. Comparisons between the two groups were performed using the t-test and Wilcoxon test. Microbial community analysis employed unweighted UniFrac distances (QIIME) for principal coordinates analysis (PCoA), while functional prediction utilized Kyoto Encyclopedia of Genes and Genomes (KEGG) pathway analysis with Kruskal-Wallis testing. All sequencing and bioinformatics were conducted by OE Biotech Co., Ltd. (Shanghai, China). Double-blinding was maintained throughout the analysis.

### 2.7 Untargeted metabolomics

Intestinal contents were lyophilized and processed for liquid chromatography (LC)-tandem mass spectrometry (MS/MS) analysis. For each sample, 15 mg of freeze-dried material was homogenized with two steel balls in 600 μL of ice-cold methanol–water (4:1, v/v) containing 4 μg/mL internal standards (L-2-chlorophenylalanine, succinate-d4, L-valine-d8, and cholic acid-d4) using a pre-cooled (−40 °C) 1.5 mL Eppendorf tube. The samples were mechanically disrupted (60 Hz, 2 min), followed by ice-water sonication (10 min) and overnight incubation at −40 °C. After centrifugation (12,000 × g, 10 min, 4 °C), 200 μL of supernatant was transferred to LC vials and dried under vacuum. The residue was reconstituted in 300 μL methanol–water (1:4, v/v), vortexed (30 s), sonicated in ice-water (3 min), and incubated at −40 °C for 2 h. Following repeat centrifugation (12,000 × g, 10 min, 4 °C), 150 μL of supernatant was filtered (0.22 μm) for LC–MS/MS analysis. A pooled quality control sample was prepared by combining equal volumes from all extracts. Chromatographic separation was achieved using an Acquity UPLC HSS T3 column (2.1 × 100 mm, 1.8 μm) on a Waters Acquity UPLC I-Class system coupled to a Thermo Q Exactive mass spectrometer. The mobile phases consisted of (A) 0.1% formic acid in water and (B) 100% acetonitrile, with the column maintained at 45 °C. The gradient elution program (2 μL injection volume) was: 0–2 min, 5% B; 4 min, 30% B; 8 min, 50% B; 10 min, 80% B; 14–15 min, 100% B; 15.1–16 min, 5% B. Mass detection employed electrospray ionization in both positive and negative modes.

MS parameters were optimized as follows: spray voltages at 3800 V (positive mode) and 3,000 V (negative mode), with auxiliary gas heater and capillary temperatures maintained at 350 °C and 320 °C, respectively. Gas flow rates were set to 35 arbitrary units (sheath gas) and 8 arbitrary units (auxiliary gas), with a mass scanning range of 100–1,200 m/z in both ionization modes. Instrument resolution was established at 70,000 for full MS scans and 17,500 for MS/MS scans. Quality control samples were analyzed every 10 injections to monitor system stability. Raw LC–MS/MS data were processed using Progenesis QI software (v3.0; Nonlinear Dynamics, Newcastle, UK) with the following tolerances: 5 ppm (precursor mass), 10 ppm (product ions), and 12 s (retention time), applying a minimum intensity threshold of 15% base peak intensity. Metabolite identification integrated retention time matching, exact mass measurements, MS/MS fragmentation patterns, and isotopic distributions, cross-referenced against the Human Metabolome Database, LIPID MAPS (v2.3), METLIN, and the proprietary LuMet-Animal database (v3.0). To ensure the reliability of qualitative and quantitative results, the following data processing and quality control procedures were applied. First, for missing value handling and zero replacement, ion features exhibiting missing values or zero intensities in >50% of samples within any group were removed. The remaining zero values were then imputed with half of the minimum ion intensity observed across all samples and features. Second, for compound identification, a score-based filtering criterion was applied. Compounds were assigned confidence scores (maximum: 80 points). Processed data were analyzed using the R ropls package following mean-centering and Pareto scaling, with orthogonal partial least squares-discriminant analysis (OPLS-DA) modeling to identify group-specific metabolic patterns. To prevent model overfitting, the OPLS-DA model was validated using seven-fold cross-validation and response permutation testing with 200 permutations. Applying a stringent FDR threshold of < 0.05 to our dataset proved overly conservative. Such an approach would severely limit our ability to capture biologically meaningful signals, thereby hindering hypothesis generation and the design of subsequent validation studies. Therefore, the FDR method was not applied in the following study. Statistically significant metabolites were selected based on variable importance projection (VIP > 1.0) and t-test analysis (*p* < 0.05) of normalized peak intensities (Supplementary Table 3). Pathway enrichment analysis was performed using KEGG metabolic pathways. All MS analyses were conducted by Shanghai Lu-Ming Biotech Co., Ltd. (Shanghai, China). Double-blinding was maintained throughout the analysis.

### 2.8 Data analysis

Statistical analysis was carried out using GraphPad Prism software, version 8.0 (San Diego, CA, U. S. A.). The data were initially analyzed for normality and homogeneity of variance using the Kolmogorov–Smirnov and Bartlett tests, respectively. Results were presented as the mean ± standard deviation (SD). The differences among groups were compared using one-way ANOVA followed by Tukey *post hoc* test, and the differences between two groups were performed using Student-*t*-test. Differences were considered to be significant at a *p* < 0.05.

## 3 Results

### 3.1 *Fucus vesiculosus*-derived fucoidan alone or in combination with simvastatin significantly ameliorated hyperlipidemia and atherosclerosis

The model group exhibited a marked elevation in average body weight, epididymal fat pad indices, plasma TC, and TG levels, increasing by 30.9% (*p* < 0.05), 1.91-fold (*p* < 0.001), 18.6-fold (*p* < 0.001), and 11.8-fold (*p* < 0.001), respectively, compared to the blank group (Table 1; Supplementary material 1). In contrast, simvastatin, the positive control drug, significantly reduced epididymal fat pad indices, plasma TC, and TG levels by approximately 4.5, 25.8% (*p* < 0.001), and 78.3% (*p* < 0.001), respectively, compared to the model group. Similarly, *F. vesiculosus*-derived fucoidan significantly lowered these values by 3.3, 16.1% (*p* < 0.001), and 76.2% (*p* < 0.001), respectively, compared to the model group (Table 1). The FSV group demonstrated a more pronounced effect, significantly decreasing epididymal fat pad indices, plasma TC, and TG levels by 34.3, 30.2% (*p* < 0.001), and 88.2% (*p* < 0.001), respectively, compared to the model group. Notably, the FSV group achieved greater reductions in plasma TC and TG levels than either simvastatin or *F. vesiculosus*-derived fucoidan monotherapy (*p* < 0.05–*p* < 0.001, Table 1). Specifically, compared with the SV and FH groups, the FSV group achieved additional reductions in TC, with decreases of 5.9 and 16.8%, and in TG, with decreases of 45.5 and 50.3%, respectively. The observed lipid alterations in each group were consistent with our prior findings (Shi et al., 2025).

**Table 1:** Effects of *F. vesiculosus* fucoidan alone and in combination with simvastatin on hyperlipidemia in New Zealand rabbits (*n* = 6).

Group	Blank	Model	SV	FH	FSV
Starting BW (kg)	1.56 ± 0.06	1.55 ± 0.05	1.57 ± 0.02	1.55 ± 0.03	1.58 ± 0.07
Ending BW (kg)	2.02 ± 0.10	2.64 ± 0.43^###^	2.62 ± 0.15^*^	2.58 ± 0.52	2.76 ± 0.08
Fat indices	2.31 ± 0.71	6.71 ± 2.20^###^	6.41 ± 1.59^*^	6.49 ± 2.05	4.41 ± 0.72^*^
TC (mmol/L)	1.26 ± 0.27	24.75 ± 0.70^###^	18.37 ± 0.65^***^	20.77 ± 0.74^***^	17.28 ± 0.59^***$&&&^
TG (mmol/L)	0.60 ± 0.08	7.69 ± 0.52^###^	1.67 ± 0.22^***^	1.83 ± 0.14^***^	0.91 ± 0.24^***$$&&^

BW, average body weight. Fat index: (epididymal fat weight ÷ body weight) × 10,000%. ^###^means *p* < 0.001 vs. Blank group; ^*^means *p* < 0.05 vs. Model group; ^***^means *p* < 0.001 vs. Model group; ^$^means *p* < 0.01 vs. SV group; ^$$^means *p* < 0.01 vs. SV group; ^&&^means *p* < 0.01 vs. FH group; ^&&&^means *p* < 0.001 vs. FH group.

There were only minor visible Oil Red O staining areas in the sections of abdominal and thoracic aortas from the BC group, while balloon catheter injury plus HFD induced significant increase in the formation of atherosclerotic plaques in the model group (Figures 1B–E). Compared to the model group, the simvastatin group reduced abdominal atherosclerotic plaque formation by 79.3% (*p* < 0.001), while the *F. vesiculosus*-derived fucoidan monotherapy group decreased it by 19.9%. The combination group exhibited a more pronounced reduction of 85.9% (*p* < 0.001). Although the combination group further decreased abdominal plaque formation compared to simvastatin alone, the difference was not statistically significant (Figure 1D). Similarly, in thoracic atherosclerotic plaque formation, the simvastatin group showed a 75.9% reduction (*p* < 0.001), whereas *F. vesiculosus*-derived fucoidan alone reduced it by 48.6% (*p* < 0.05). The combination group achieved the highest reduction at 78.6% (*p* < 0.001, Figure 1E). These results demonstrate that *F. vesiculosus*-derived fucoidan, both alone and in combination with simvastatin, significantly ameliorates atherosclerosis. Consistent with our previous report (Shi et al., 2025), the alterations in atherosclerotic plaque were observed across all groups.

### 3.2 *Fucus vesiculosus*-derived fucoidan alone or in combination with simvastatin modulated the component of gut microbiota

**Figure 2:**
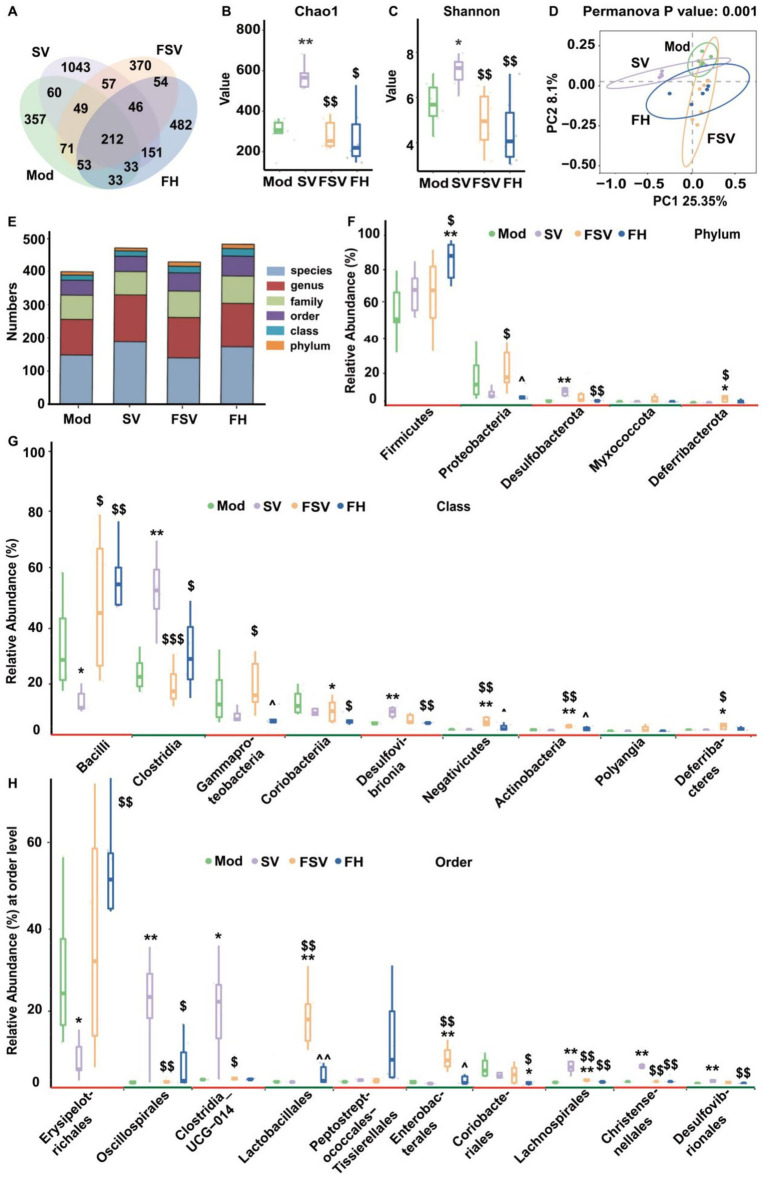
Effects of *F. vesiculosus*-derived fucoidan alone or in combination with simvastatin on the abundance of gut microbiota (*n* = 6). **(A)** ASV Venn graph; **(B)** α-diversity (Chao 1 graph); **(C)** α-diversity (Shannon graph); **(D)** β-diversity (unweighted PCoA); **(E)** The average levels of gut microbiota species in different classification levels. The relative abundance of gut microbiota at **(F)** Phylum; **(G)** Class; and **(H)** Order levels. ^*^p < 0.05 *vs.* Mod group; ^**^p < 0.01 *vs.* Mod group; ^$^*p* < 0.05 *vs.* SV group; ^$$^*p* < 0.01 *vs.* SV group; ^$$$^*p* < 0.001 *vs.* SV group; ^^^*p* < 0.05 *vs.* FSV group; ^^^^*p* < 0.01 *vs.* FSV group.

The rarefaction curves for ASVs are presented in Supplementary Figure 1. The curves plateau as sequencing read count increases, suggesting that the sequencing depth is adequate for capturing most of the microbial diversity, and that further sequencing would generate only a limited number of additional ASVs. The Venn diagram clearly demonstrated that the intervention groups, particularly the SV group, significantly increased the number of ASVs compared to the model group (Figure 2A, Supplementary material 2). Specifically, the SV intervention group exhibited a higher median number and greater dispersion of observed species, as confirmed by *α*-diversity analysis using Chao1 (*p* < 0.01, Figure 2B) and Shannon indices (*p* < 0.05, Figure 2C). Additionally, *β*-diversity analysis revealed that the intervention groups displayed higher levels of species dispersion than the model group (Figure 2D; Supplementary Figure 2). These findings were further supported by Figure 2E, which showed that the intervention groups increased the total number of observed species across different taxonomic levels.

**Figure 3:**
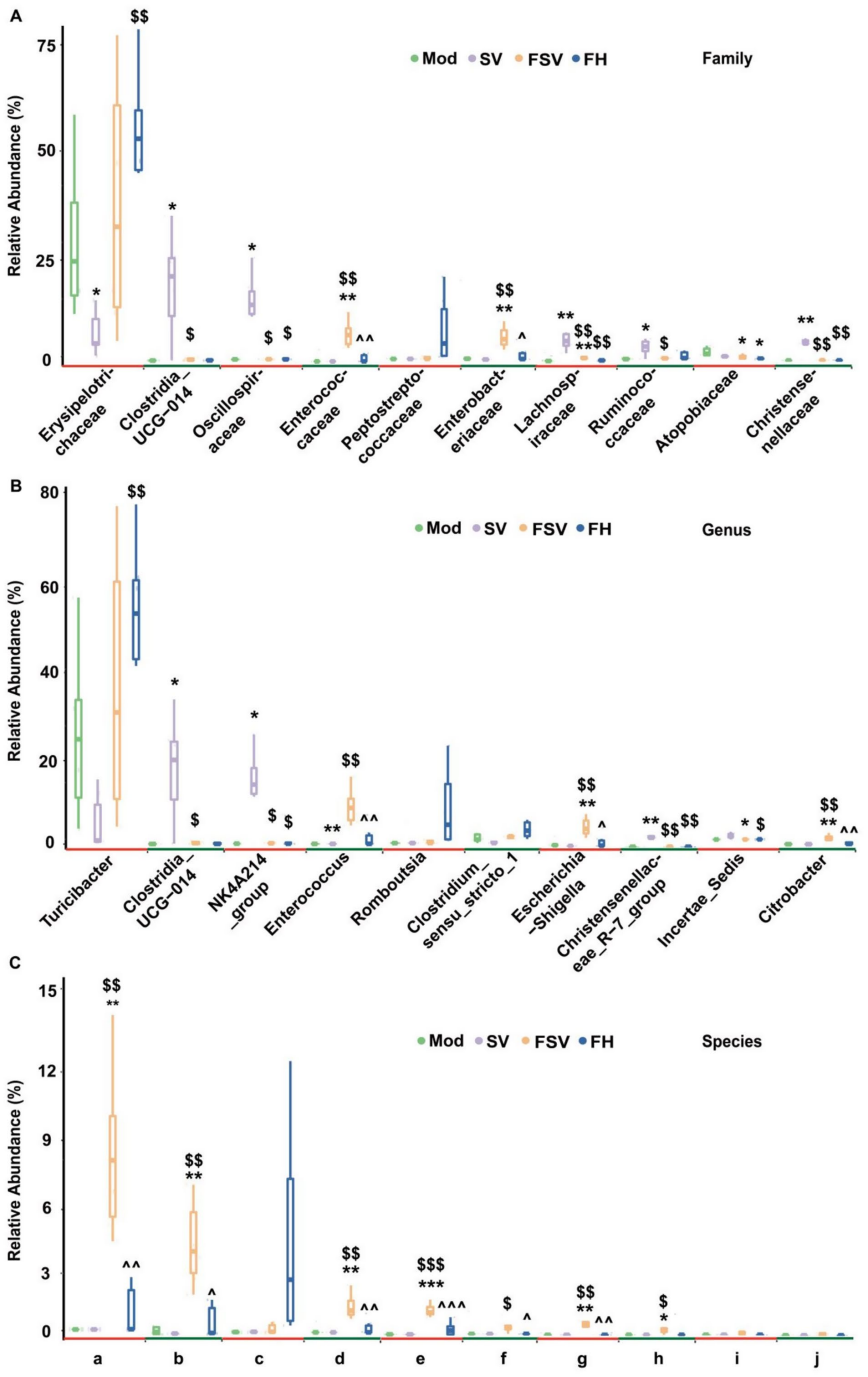
Effects of *F. vesiculosus*-derived fucoidan alone or in combination with simvastatin on the relative abundance of gut microbiota at **(A)** Family; **(B)** Genus; and **(C)** Species levels (*n* = 6). The abbreviations in panel **(C)**. a, *Enterococcus_durans_g_Enterococcus*; b, *Escherichia_coli_g_Escherichia-Shigella*; c, *Romboutsia_ilealis_g_Romboutsia*; d, *Citrobacter_amalonaticus_g_Citrobacter*; e, *Clostridium_butyricum_g_Clostridium_sensu_stricto_1*; f, *Faecalibacterium_prausnitzii_g_Faecaliba*; g, *Bifidobacterium_dentium_g_Bifidobacterium*; h, *Veillonella_ratti_g_Veillonella*; i, *Anaerostipes_caccae_g_Anaerostipes*; j, *Porphyromonas_asaccharolytica_g_Porphyro*. *p < 0.05 *vs.* Mod group; ^**^p < 0.01 *vs.* Mod group; **^*^p < 0.001 *vs.* Mod group; ^$^*p* < 0.05 *vs.* SV group; ^$$^*p* < 0.01 *vs.* SV group; ^$$$^*p* < 0.001 *vs.* SV group; ^*p* < 0.05 *vs.* FSV group; ^^^^*p* < 0.01 *vs.* FSV group; ^^^^^*p* < 0.001 *vs.* FSV group.

The gut microbiota data are presented in Supplementary material 2. Compared to the model group, the SV intervention group significantly increased the abundance of *p_Desulfobacterota* (*p* < 0.01), the FH group significantly increased the abundance of *p_Firmicutes* (*p* < 0.01), while the combined therapy group dramatically increased the abundance levels of *p_Deferribacterota* (*p* < 0.05, Figure 2F). At the class level, the SV intervention group significantly decreased the abundance of *c_Bacilli* (*p* < 0.05) and increased the abundance levels of *c_Clostridia* and *c_Desulfovibrionia*, compared to the model group (*p* < 0.01, Figure 2G). The combined therapy group significantly increased the abundance levels of *c_Gammaproteobacteria*, *c_Negativicutes*, and *c_Actinobacteria* compared to the SV or FH intervention group (*p* < 0.05 or *p* < 0.01, Figure 2G). At the order level, the SV intervention group decreased the abundance of *o_Erysipelotrichales* and dramatically increased the abundance levels of *o_Oscillospirales*, *o_Clostridia_UCG-014*, *o_Lachnospirales*, *o_Christensenellales*, and *o_Desulfovibrionales*, compared to that of the model group (*p* < 0.05 or *p* < 0.01, Figure 2H). Compared to the rest groups, the combined therapy group significantly increased the abundance of *o_Lactobacillales* and *o_Enterobacterales* (*p* < 0.01 or *p* < 0.05, Figure 2H). At the family level, the SV intervention group significantly decreased the abundance of *f_Erysipelotrichaceae* and dramatically increased the abundance levels of *f_Clostridia_UCG-014*, *f_Oscillospiraceae*, *f_Lachnospiraceae*, *f_Ruminococcaceae* and *f_Christensenellaceae*, while the combined therapy and FH intervention groups significantly decreased the abundance of *f_Atopobiaceae*, compared to those of the model group (*p* < 0.05 or *p* < 0.01, Figure 3A). Compared to the rest groups, the combined therapy group significantly increased the abundance of *f_Enterococcaceae* and *f_Enterobacteriaceae* (*p* < 0.05 or *p* < 0.01, Figure 3A). At the genus level (Figure 3B), compared to the model group, the SV intervention group dramatically increased the abundance of *g_Clostridia_UCG-014*, *g_NK4A214*, *g_Christensenellaceae_R-7_group*, and *g_Incertae_Sedis* (*p* < 0.05 or *p* < 0.01). Compared to the rest groups, the combined therapy group significantly increased the abundance levels of *g_Enterococcus*, *g_Escherichia-Shigella*, and *g_Citrobacter* (*p* < 0.05 or *p* < 0.01). At the species level (Figure 3C), the combined therapy group significantly increased the abundance levels of *s_Enterococcus_durans_g_Enterococcus* (a), *s_Escherichia_coli_g_Escherichia-Shigella* (b), and *s_Citrobacter_amalonaticus_g_Citrobacter* (d), *s_Clostridium_butyricum_g_Clostridium_sensu_stricto_1* (e), *s_Faecalibacterium_prausnitzii_g_Faecaliba* (f), and *s_Bifidobacterium_dentium_g_Bifidobacterium* (g), compared to the rest groups (*p* < 0.05, *p* < 0.01 or *p* < 0.001).

**Figure 4:**
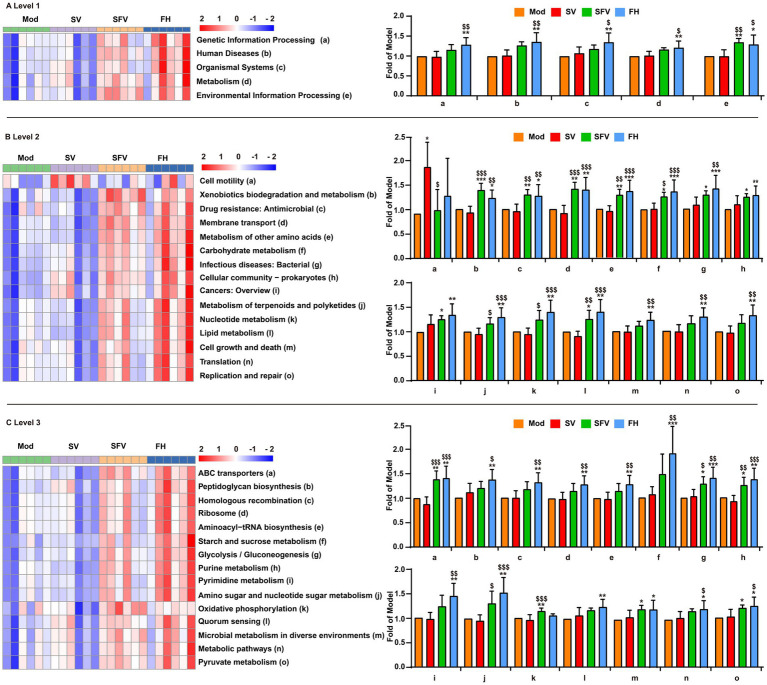
KEGG analysis of predicted functions of the enriched gut microbiota at **(A)**, level 1, **(B)**, level 2; and **(C)**, level 3 (*n* = 6). The abbreviations in panel **(A)**. a, Genetic information processing; b, Human diseases; c, Organismal systems; d, Metabolism; e, Environmental information processing. The abbreviations in panel **(B)**. a, Cell motility; b, Xenobiotics biodegradation and metabolism; c, Drug resistance: antimicrobial; d, Membrane transport; e, Metabolism of other amino acids; f, Carbohydrate metabolism; g, Infectious diseases: bacterial; h, Cellular community-prokaryotes; i, Cancers: overview; j, Metabolism of terpenoids and polyketides; k, Nucleotide metabolism; l, Lipid metabolism; m, Cell growth and death; n, Translation; o, Replication and repair. The abbreviations in panel **(C)**. a, ABC transporters; b, Peptidoglycan biosynthesis; c, Homologous recombination; d, Ribosome; e, Aminoacyl-tRNA biosynthesis; f, Starch and sucrose metabolism; g, Glycolysis/gluconeogenesis; h, Purine metabolism; i, Pyrimidine metabolism; j, Amino sugar and nucleotide sugar metabolism; k, Oxidative phosphorylation; l, Quorum sensing; m, Microbial metabolism in diverse environments; n, Metabolic pathways; o, Pyruvate metabolism. ^*^p < 0.05 *vs.* Mod group; ^**^p < 0.01 *vs.* Mod group; ^***^p < 0.001 *vs.* Mod group; ^$^*p* < 0.05 *vs.* SV group; ^$$^p < 0.01 *vs.* SV group; ^$$$^*p* < 0.001 *vs.* SV group.

KEGG analysis categorized enriched microbiota gene functions into three hierarchical levels (Figures 4A–C), revealing that the intervention groups, particularly FSV and FH, significantly upregulated multiple terms related to human diseases, organismal systems, metabolism, and environmental information processing. Most notably, the FH and FSV intervention groups demonstrated marked enhancement in key metabolic pathways including xenobiotics biodegradation and metabolism, metabolism of other amino acids, carbohydrate metabolism, metabolism of terpenoids, and polyketides, nucleotide metabolism, lipid metabolism, ATP-binding cassette (ABC) transporters, peptidoglycan biosynthesis, aminoacyl-tRNA biosynthesis, starch and sucrose metabolism, purine metabolism, pyrimidine metabolism, amino sugar and nucleotide sugar metabolism, microbial metabolism in diverse environments, and pyruvate metabolism.

### 3.3 *Fucus vesiculosus*-derived fucoidan alone or in combination with simvastatin affected gut microbiota-derived metabolites

**Figure 5:**
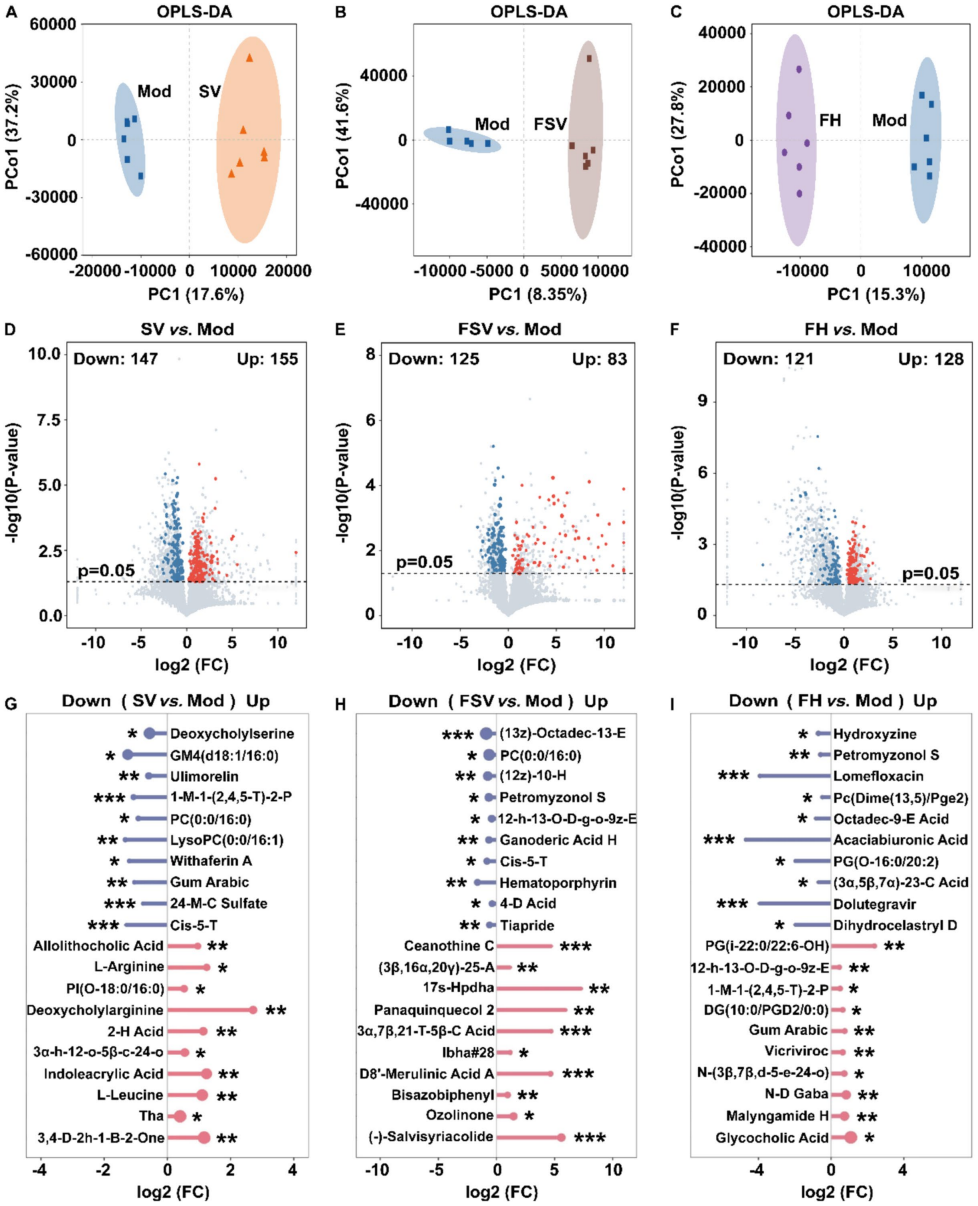
Effects of *F. vesiculosus*-derived fucoidan alone or in combination with simvastatin on gut microbiota metabolites (*n* = 6). OPLS-DA graph of **(A)** the SV group *vs.* the model group; **(B)** the FSV group *vs.* the model group; and **(C)** the FH group *vs.* the model group. The volcano plot graph of **(D)** the SV group *vs.* the model group; **(E)** the FSV group *vs.* the model group; and **(F)** the FH group *vs.* the model group. The top 10 downregulated and upregulated metabolites of **(G)** the SV group *vs.* the model group; **(H)** the FSV group *vs.* the model group; and **(I)** the FH group *vs.* the model group. The abbreviations for the metabolites in **(G–I)**. 1-M-1-(2,4,5-T)-2-P: 1-Methoxy-1-(2,4,5-Trimethoxyphenyl)-2-Propanol; 12-h-13-O-D-g-o-9z-E: 12-Hydroxy-13-O-D-Glucuronoside-Octadec-9z-Enoate; (12z)-10-H: (12z)-10-Hydroxyoctadecenoylcarnitine; (13z)-Octadec-13-E: (13z)-Octadec-13-Enoylcarnitine; 24-M-C Sulfate: 24-Methylene-Cholesterol Sulfate; 2-H Acid: 2-Hydroxycinnamic Acid; 3,4-D-2 h-1-B-2-One: 3,4-Dihydro-2 h-1-Benzopyran-2-One; (3α,5*β*,7α)-23-C Acid: (3α,5β,7α)-23-Carboxy-7-Hydroxy-24-Norcholan-3-Yl-B-D-Glucopyranosiduronic Acid; 3α,7β,21-T-5β-C Acid: 3α,7β,21-Trihydroxy-5β-Cholanoic Acid; 3α-h-12-o-5β-c-24-o: 3alpha-Hydroxy-12-Oxo-5beta-Cholan-24-Oic Acid; (3β,16α,20γ)-25-A: (3β,16α,20γ)-25-Acetoxy-3,16,20,22-Tetrahydroxy-5-Cucurbiten-11-One 3-Glucoside; 4-D Acid: 4-Dodecylbenzenesulfonic Acid; Cis-5-T: Cis-5-Tetradecenoylcarnitine; Dihydrocelastryl D: Dihydrocelastryl Diacetate; N-(3β,7β,d-5-e-24-o): N-(3beta,7beta, Dihydroxycholest-5-En-24-Oyl) Glycine; N-D Gaba: N-Docosahexaenoyl Gaba; Octadec-9-E Acid: Octadec-9-Enoic Acid; Petromyzonol S: Petromyzonol Sulfate; PG(i-22:0/22:6-OH): PG(i-22:0/22:6(4Z,7Z,10Z,12E,16Z,19Z)-OH(14)); PG(O-16:0/20:2): PG(O-16:0/20:2(11Z,14Z)). ^*^p < 0.05 *vs.* Mod group; ^**^p < 0.01 *vs.* Mod group; ^***^p < 0.001 *vs.* Mod group.

The OPLS-DA analysis revealed distinct separation between the gut metabolite profiles of the SV, FSV, and FH intervention groups compared to the model group (Figures 5A–C; Supplementary material 3). Generally, for the comparison group in conventional correspondence analysis, larger R2Y and Q2 values indicate better model performance. In the permutation test, a positive slope of the Q2 regression line is expected, and an intercept below zero on the y-axis is considered desirable. The permutation data suggested that our LC–MS/MS results were reliable (Supplementary Figure 3). Metabolomic profiling demonstrated that the SV, combination therapy (FSV), and FH interventions significantly upregulated 155, 83, and 128 metabolites while downregulating 147, 125, and 121 metabolites, respectively, relative to the model group (Figures 5D–F). Visualization of these metabolic changes through Lolipopmap highlighted the top 10 most significantly altered metabolites in each comparison group (Figures 5G–I), which predominantly comprised lipids, amino acids, and various organic molecules showing substantial quantitative differences between groups.

**Figure 6:**
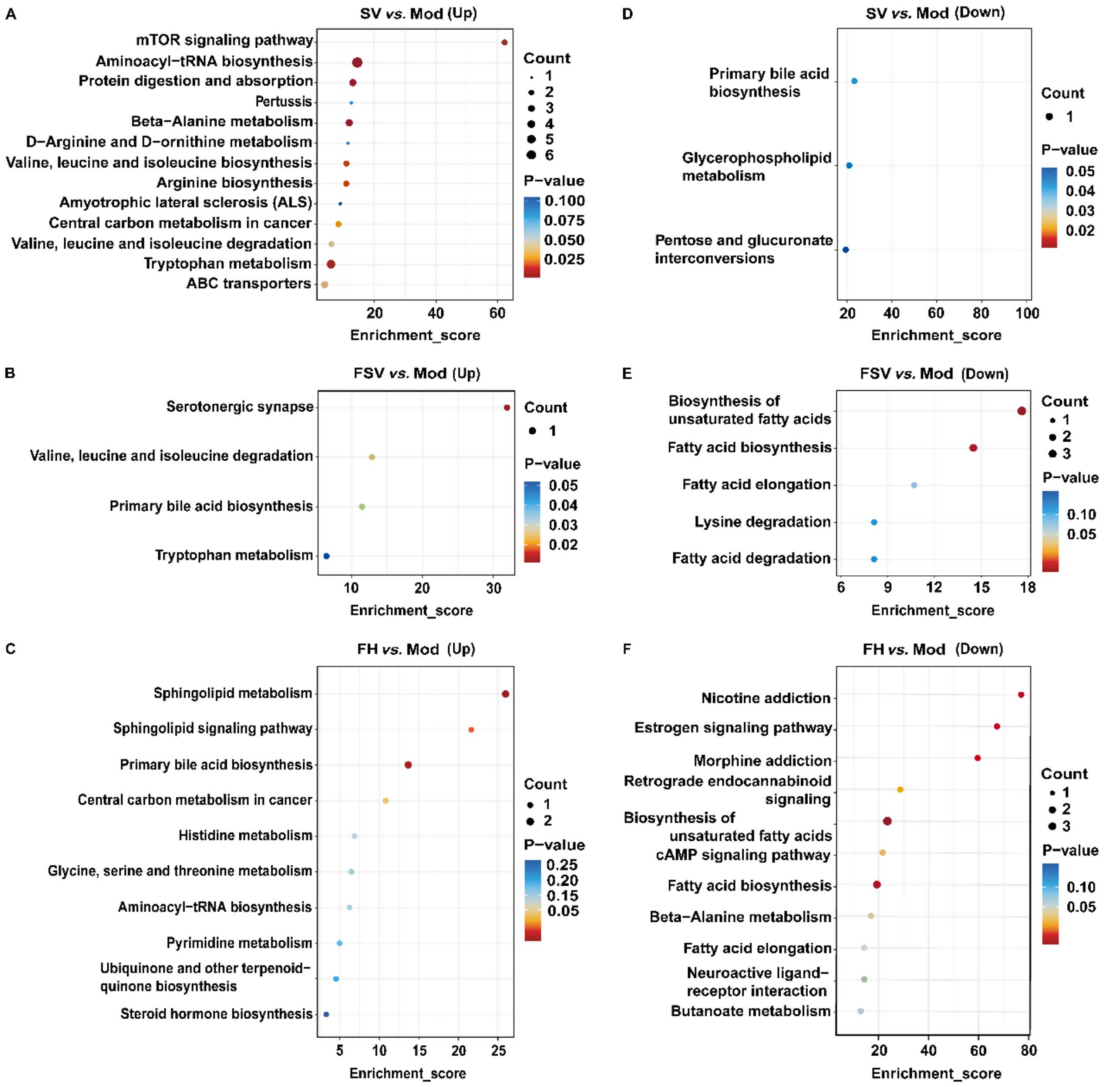
KEGG analysis of the enriched gut microbiota-derived metabolites (*n* = 6). The top upregulated KEGG terms of **(A)** the SV group *vs.* the model group; **(B)** the FSV group *vs.* the model group; and **(C)** the FH group *vs.* the model group. The top downregulated KEGG terms of **(D)** the SV group *vs.* the model group; **(E)** the FSV group *vs.* the model group; **(F)** the FH group *vs.* the model group.

KEGG enrichment analysis revealed distinct metabolic pathway alterations across intervention groups, with the SV group showing significant upregulation of aminoacyl-tRNA biosynthesis, protein digestion and absorption, amino acid metabolism, and ABC transporters, while downregulating primary bile acid biosynthesis, glycerophospholipid metabolism, and pentose and glucuronate interconversions (Figures 6A,D). This group also elevated disease-related pathways including pertussis and amyotrophic lateral sclerosis (ALS) (Figure 6A). The combined therapy group demonstrated unique regulatory patterns, upregulating amino acid metabolism, serotonergic synapse, and primary bile acid biosynthesis while suppressing fatty acid biosynthesis, fatty acid elongation, and lysine degradation (Figures 6B,E). Most notably, the FH intervention group exhibited the most comprehensive metabolic modulation, significantly enhancing sphingolipid metabolism, primary bile acid biosynthesis, steroid hormone biosynthesis, aminoacyl-tRNA biosynthesis, pyrimidine metabolism, and amino acid metabolism, while reducing fatty acid biosynthesis and certain amino acid metabolic pathways (Figures 6C,F).

**Figure 7:**
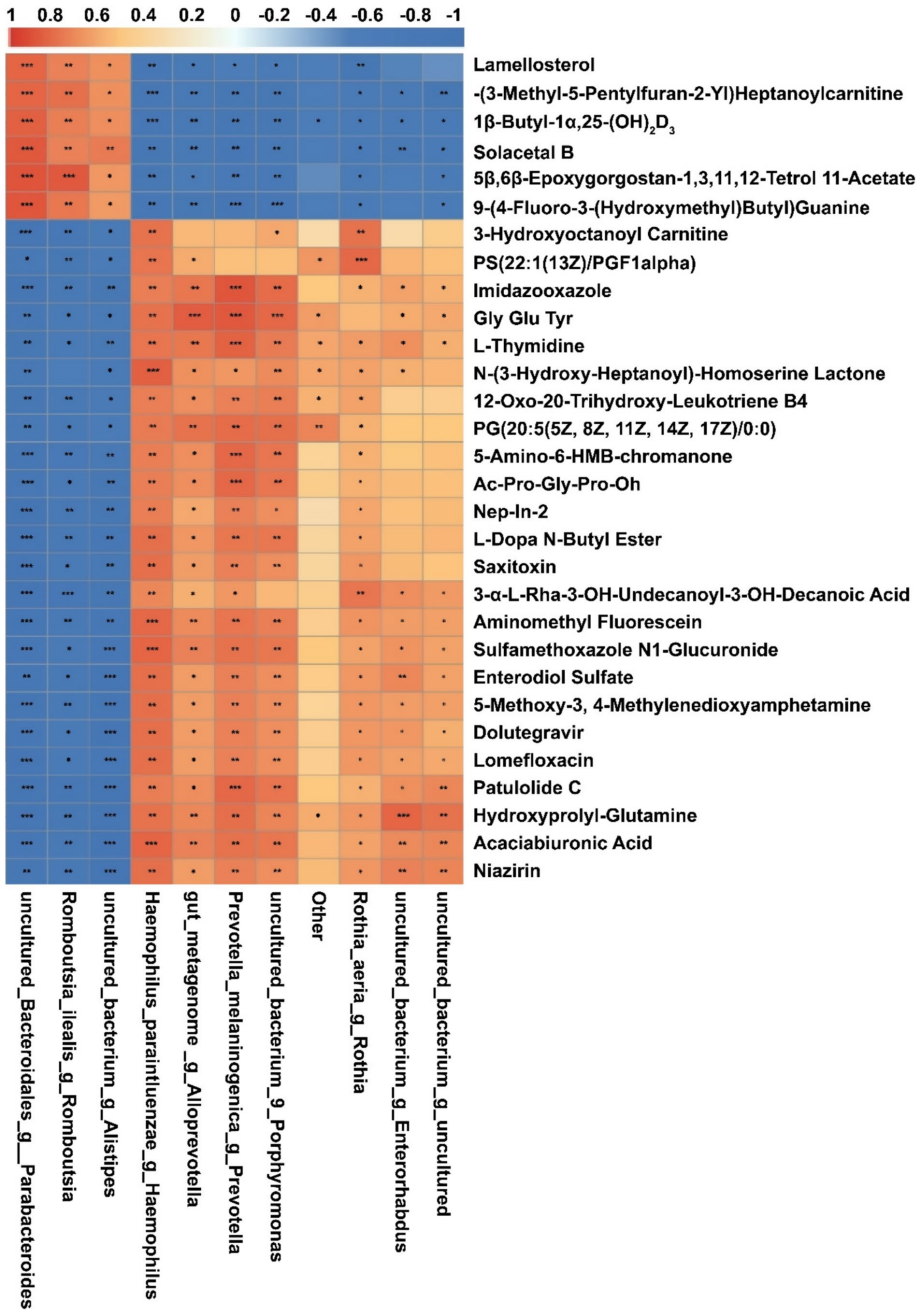
Spearman correlation analysis between significantly regulated gut microbiota and metabolites in the SV group versus the model group. The abbreviations for the gut microbiota. 1β-Butyl-1α,25-(OH)_2_D_3_: 1beta-Butyl-1alpt dihydroxyvitamin D3/1beta-Butyl-1alpha, 25-dihydroxycholecalciferol; 5β, 6β-Epoxygorgostan-1, 3, 11, 12-Tetrol 11-Acetate: 5beta, 6beta-Epoxygorgostan-1alpha, 3beta, 11alpha, 12beta-tetrol 11-acetate; 5-Amino-6-HMB-chromanone: 5-Amino-2, 3-dihydro-6-(3-hydroxy-4-methoxy-1-oxobutyl)-2, 2-dimethyl-4H-1-benzopyran-4-one; 3-α-L-Rha-3-OH-Undecanoyl-3-OH-Decanoic Acid: 3-*O*-Alpha-L-rhamnopyranosyl-3-hydroxyunde canoyl-3-hydroxydecanoic acid. ^*^p < 0.05; ^**^p < 0.01; ^***^p < 0.001.

**Figure 8:**
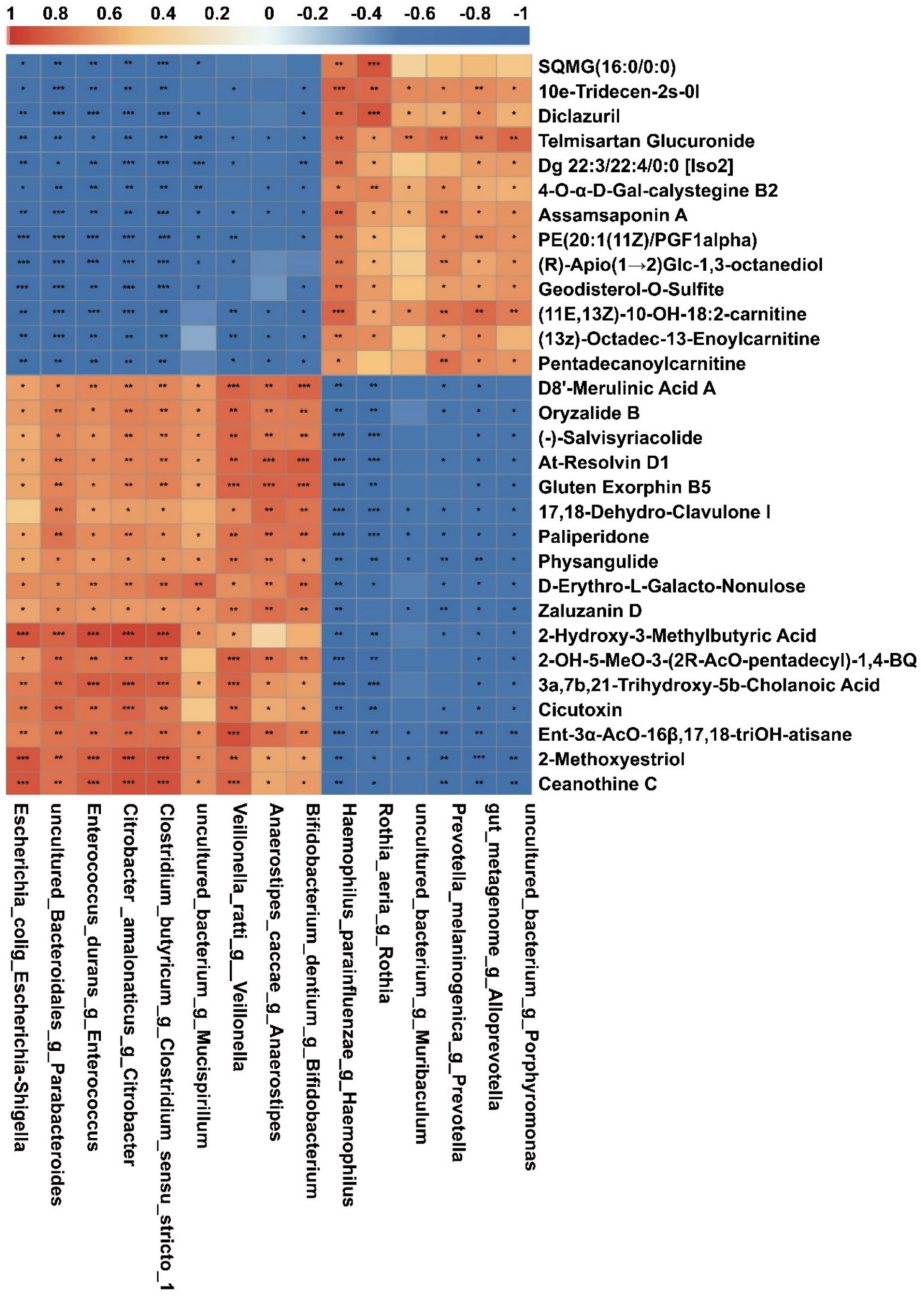
Spearman correlation analysis between significantly regulated gut microbiota and metabolites in the FSV group versus the model group. The abbreviations for the gut microbiota. Dg 22:3/22:4/0:0 [Iso2]: Dg(22:3(10*z*, 13*z*, 16*z*)/22:4(7*z*, 10*z*, 13*z*, 16*z*)/0:0)[Iso2]; 4-O-α-D-Gal-calystegine B2: 4-O-Alpha-D-Galactopyranosylcalystegine B2; (R)-Apio(1 → 2)Glc-1,3-octanediol: (R)-1-O-[B-D-Apiofuranosyl-(1- > 2)-B-D- Glucopyranoside]-1, 3-Octanediol; (11E,13Z)-10-OH-18: 2-carnitine: (11e, 13*z*)-10-Hydroxyoctadeca-11, 13-Dienoylcarnitine; 2-OH-5-MeO-3-(2R-AcO-pentadecyl)-1,4-BQ: 2-Hydroxy-5-Methoxy-3-(2r-Acetoxy-Pentadecyl)-1, 4-Benzoquinone; Ent-3α-AcO-16β,17,18-triOH-atisane: Ent-3alpha-Acetoxy-16beta, 17, 18-Trihydroxyatisane. ^*^p < 0.05; ^**^p < 0.01; ^***^p < 0.001.

Spearman correlation analysis revealed the correlations between differentially expressed microbiota and metabolites (Figures 7, 8; Table 2; Supplementary Figure 4). In the FH group, for instance, the upregulated metabolites—lamellosterol B and solacetal B—showed positively correlations with *p_Firmicutes*, *o_Peptostreptococcales-Tissierellales*, *f_Peptostreptococcaceae*, *f_Rhizobiaceae*, *g_ Rikenellaceae_RC9_gut_group*, *s_uncultured_Bacteroidales_g__Parabacteroides*, and *s_Romboutsia_ilealis_g__Romboutsia*. In contrast, they were negatively correlated with *c_Coriobacteriia*, *o_Pasteurellales*, *f_Pasteurellaceae*, *f_Porphyromonadaceae*, *g_Haemophilus*, *g_Porphyromonas*, *s_Haemophilus_parainfluenzae_g__Haemophilus*, and *s_ gut_metagenome_g__Alloprevotella*. Similarly, the down-regulated metabolites—lomefloxacin, dolutegravir, acaciabiuronic acid, and amino acids (glycine, glutamic acid, tryptophan)—exhibited positively correlations with *p_Actinobacteriota*, *c_Coriobacteriia*, *o_Pasteurellales*, *f_Atopobiaceae*, *f_Pasteurellaceae*, *g_Haemophilus*, *s_Haemophilus_parainfluenzae_g_Haemophilus*, *s_Prevotella_melaninogenica_g_Prevotella*, and *s_uncultured_bacterium_g_Porphyromonas*. In contrast, they were negatively correlated with *p_Firmicutes*, *c_Alphaproteobacteria*, *o_Peptostreptococcales-Tissierellales*, *o_Rhizobiales*, *f_Peptostreptococcaceae*, *f_Rhizobiaceae*, *g_Allorhizobium-Neorhizobium-Pararhizobium-Rhizobium*, *g_Rikenellaceae_RCg_gut_group*, and *s_uncultured_Bacteroidales_g_Parabacteroides* (Figure 7; Supplementary Figure 4). In the FSV group, for instance, the upregulated metabolites—D8’-merulinic acid A, (−)-salvisyriacolide, and ceanothine C—showed positive correlations with *p_Deferribacterota*, *c_Actinobacteria*, *c_Negativicutes*, *c_Deferribacteres*, *o_Lactobacillales*, *o_Lachnospirales*, *f_Lachnospiraceae*, *g_Veillonella*, *s_ Clostridium_butyricum_g__Clostridium_sensu_stricto_1*, *s_Citrobacter_amalonaticus_g__Citrobacter*, and *s_Veillonella_ratti_g__Veillonella*. In contrast, they were negatively correlated with *o_Pasteurellales*, *f_Pasteurellaceae*, *f_ Neisseriaceae*, *f_ Streptococcaceae*, *g_Haemophilus*, *g_Rothia*, *s_Haemophilus_parainfluenzae_g__Haemophilus*, and *s_Rothia_aeria_g__Rothia* (Figure 8; Supplementary Figure 4). Additional correlations between differentially expressed microbiota and metabolites can be found in Supplementary Figure 4 and Supplementary material 4.

**Table 2:** Gut metabolites of rabbits affected by the simvastatin (SV), combined *F. vesiculosus* fucoidan and simvastatin (FSV), and *F. vesiculosus*-derived fucoidan (FH), as compared to the model group (*n* = 6).

Groups	Terms	Metabolites
SV *vs.* Model	mTOR signaling pathway↑	L-arginine, L-leucine↑
	Chagas disease (American trypanosomiasis)↑	L-arginine↑
	African trypanosomiasis↑	L-tryptophan↑
	Aminoacyl-tRNA biosynthesis↑	L-lysine, L-arginine, L-tryptophan, L-phenylalanine, L-leucine, L-histidine↑
	Protein digestion and absorption↑	L-lysine, L-arginine, L-phenylalanine↑
	Pertussis↑	Nicotinic acid↑
	beta-Alanine metabolism↑	L-histidine, carnosine, pantothenic acid↑
	Choline metabolism in cancer↑	Glycerophosphocholine↑
	D-Arginine and D-ornithine metabolism↑	L-arginine↑
	Valine, leucine and isoleucine biosynthesis↑	L-leucine, ketoleucine↑
	Arginine biosynthesis↑	L-arginine, citrulline↑
	Amoebiasis↑	L-arginine↑
	Amyotrophic lateral sclerosis (ALS)↑	L-arginine↑
	Central carbon metabolism in cancer↑	L-arginine, L-leucine↑
	Phenylalanine, tyrosine and tryptophan biosynthesis↑	L-tryptophan, L-phenylalanine↑
	Valine, leucine and isoleucine degradation↑	L-Leucine, ketoleucine↑
	Tryptophan metabolism↑	L-tryptophan, Indoleacetaldehyde, 2-formaminobenzoylacetate, picolinic acid↑
	Histidine metabolism↑	L-histidine, carnosine↑
	Phenylalanine metabolism↑	L-phenylalanine, 2-hydroxycinnamic acid↑
	ABC transporters↑	L-arginine, L-lysine, L-histidine↑
	Choline metabolism in cancer↓	PC (16:1(9z)/0:0)↓
	Primary bile acid biosynthesis↓	5b-cyprinol sulfate↓
	Glycerophospholipid metabolism↓	PC(16:1(9Z)/0:0) ↓
	Pentose and glucuronate interconversions↓	25-hydroxyvitamin D2-25-glucuronide↓
FSV *vs.* Model	Serotonergic synapse↑	5-hydroxy-L-tryptophan↑
	Valine, leucine and isoleucine biosynthesis↑	Ketoleucine↑
	Valine, leucine and isoleucine degradation↑	Ketoleucine↑
	alpha-Linolenic acid metabolism↑	Stearidonic acid↑
	Primary bile acid biosynthesis↑	7α-hydroxy-3-oxocholest-4-en-27-oic acid↑
	Pentose and glucuronate interconversions↑	Phenethylamine glucuronide↑
	Pyrimidine metabolism↑	6-dehydrotestosterone 17-glucosiduronic acid↑
	Tryptophan metabolism↑	5-hydroxy-L-tryptophan↑
	Valine, leucine and isoleucine biosynthesis↓	Isopropylmaleic acid↓
	alpha-Linolenic acid metabolism↓	9 s-hotre↓
	Pentose and glucuronate interconversions↓	25-hydroxyvitamin D2-25-glucuronide↓
	Pyrimidine metabolism↓	Pseudouridine↓
	Biosynthesis of unsaturated fatty acids↓	Palmitic acid, stearic acid, cis-gondoic acid↓
	Fatty acid biosynthesis↓	Palmitic acid, stearic Acid↓
	Fatty acid elongation↓	Palmitic acid↓
	Lysine degradation↓	4-Trimethylammoniobutanoic acid↓
	Fatty acid degradation↓	Palmitic acid↓
FH *vs.* Model	Sphingolipid metabolism↑	Sphinganine, Phytosphingosine↑
	Sphingolipid signaling pathway↑	Sphinganine↑
	Primary bile acid biosynthesis↑	Glycocholic acid, 3α,7α,12α-trihydroxy-5β-cholestan-26-oic acid↑
	Alanine, aspartate and glutamate metabolism↑	L-asparagine↑
	Central carbon metabolism in cancer↑	L-asparagine↑
	Histidine metabolism↑	Urocanic acid↑
	Glycine, serine and threonine metabolism↑	Creatine↑
	Aminoacyl-tRNA biosynthesis↑	L-asparagine↑
	Pyrimidine metabolism↑	Cytidine↑
	Ubiquinone and other terpenoid-quinone biosynthesis↑	Demethylphylloquinone↑
	Arginine and proline metabolism↑	Creatine↑
	Steroid hormone biosynthesis↑	Estrone 3-glucuronide↑
	Alanine, aspartate and glutamate metabolism↓	4-amino-butanoic acid↓
	Nicotine addiction↓	4-amino-butanoic acid↓
	Estrogen signaling pathway↓	4-amino-butanoic acid↓
	Morphine addiction↓	4-amino-butanoic acid↓
	Arginine and proline metabolism↓	4-amino-butanoic acid↓
	GnRH secretion↓	4-amino-butanoic acid↓
	GABAergic synapse↓	4-amino-butanoic acid↓
	Retrograde endocannabinoid signaling↓	4-amino-butanoic acid↓
	Biosynthesis of unsaturated fatty acids↓	Palmitic acid, stearic acid, cis-gondoic acid↓
	Taste transduction↓	4-amino-butanoic acid↓
	cAMP signaling pathway↓	4-amino-butanoic acid↓
	Fatty acid biosynthesis↓	Palmitic acid, stearic acid↓
	beta-Alanine metabolism↓	4-amino-butanoic acid↓
	Fatty acid elongation↓	Palmitic acid↓
	Neuroactive ligand-receptor interaction↓	4-amino-butanoic acid↓
	Butanoate metabolism↓	4-amino-butanoic acid↓
	Lysine degradation↓	4-trimethylammoniobutanoic acid↓
	Fatty acid degradation↓	Palmitic acid↓
	Nicotinate and nicotinamide metabolism↓	4-amino-butanoic acid↓
	Tryptophan metabolism↓	2-formaminobenzoylacetate↓

## 4 Discussion

The main chain of *F. vesiculosus* fucoidan has been shown to consist of alternating [→4)-*α*-L-Fuc*p*(1 → 3)-α-L-Fuc*p*(1→] glycosyl residues, with side chains composed of other sugar residues such as galactose, glucose, glucuronic acid, and xylose (Wang et al., 2025; Xing et al., 2023; Chevolot et al., 1999; Chevolot et al., 2001; Pereira et al., 1999). As discussed in the “Introduction,” rabbits exhibit several advantages over rodents for modeling human dyslipidemia and atherosclerosis, most notably that their lipid profiles and plaque characteristics closely resemble those of hyperlipidemic patients with atherosclerosis (Dabravolski et al., 2025; Hayashi et al., 2000, 2023; Kamato et al., 2022). These advantages facilitate the translation of findings from this rabbit model into clinical practice. To the best of our knowledge, this study is the first to investigate the gut microbiota-regulatory effects of fucoidan, both alone and in combination with simvastatin, using an HFD-fed rabbit model with balloon catheter injury. Our findings extend beyond our previous reports (Shi et al., 2025; Wang et al., 2025), which focused on the lipid-lowering, anti-inflammatory, and general safety of this combination therapy. This work highlights the intricate interplay between the host, gut microbes, and their metabolites as a pivotal mechanism. The findings suggest that the anti-atherosclerotic effect of the combination of *F. vesiculosus* fucoidan and simvastatin is associated with alterations in the gut microbiota and its derived microbial metabolites.

Importantly, the dosages of both simvastatin and fucoidan used in this investigation were precisely converted as aforementioned in the “Method.” Specifically, the rabbit doses of fucoidan (100 mg/kg) and simvastatin (5 mg/kg) used in this study correspond to human equivalent doses of approximately 900 mg fucoidan per day and 40 mg simvastatin per day for an adult. These dosages hold potential for direct translation into clinical application. However, the actual clinical dosage of fucoidan, as well as the appropriate dosage in rabbits, needs to be determined in future studies. To the best of our knowledge, the specific t_1/2_ of fucoidan following oral administration in rabbits is not available. However, one study reported this parameter in New Zealand rabbits after subcutaneous and intravenous injection (Cattaneo et al., 2002). The t_1/2_ of the *β* phase ranged from 5.2 h to 25.8 h for subcutaneous injection, and from 0.32 h to 1.2 h for intravenous injection. Furthermore, the t_1/2_ of fucoidan has been shown to increase with molecular weight. For example, following intravenous injection in rats, the t_1/2_ of low-molecular-weight fucoidan (~8 kDa) was approximately 1 h (Deux et al., 2002). When the molecular weight was increased to 19 kDa, the t_1/2_ extended to approximately 2.0 h (Deux et al., 2009). Notably, the pharmacokinetic parameters obtained from rats can be extrapolated to rabbits using formulas from the literatures (van Valkengoed et al., 2025; Greenblatt, 1985; Caldwell et al., 2004; Karalis et al., 2001). The rat-specific values employed for this interspecies conversion were derived from the references (Deux et al., 2009; Deux et al., 2002; Pozharitskaya et al., 2018; Zhang et al., 2020). After careful calculation, the t_1/2_ of fucoidan in rabbits is presumed to range from 1.3 h to 2.4 h. This range corresponds to molecular weights increasing from 6.9 kDa to 19.0 kDa. Given that the fucoidan FvF has a substantially higher molecular weight of ~517.6 kDa, its t_1/2_ is expected to be longer than these calculated values. However, its actual t_1/2_ needs to be determined in future studies.

Lipid disorders, particularly elevated levels of TC and TG, have been shown to contribute to atherosclerosis (Lee et al., 2025; Zhang et al., 2022). In this study, *F. vesiculosus* fucoidan significantly reduced plasma TC and TG levels in hyperlipidemic rabbits in a dose-dependent manner. Compared to monotherapy groups, the combination of *F. vesiculosus* fucoidan and simvastatin further improved TC and TG levels. These lipid-regulatory effects align with findings from other brown algae-derived fucoidans in apolipoprotein E-deficient mice and obese individuals (Liu T. et al., 2024; Łagowska et al., 2025; Yin et al., 2019; Yang et al., 2019; Zhang et al., 2020; Yokota et al., 2016). The hypolipidemic properties of *F. vesiculosus* fucoidan may be attributed to its carbohydrate and glucuronic acid composition, sulfate content, and substitution patterns, as evidenced by prior rodent studies (Yang et al., 2022; Liu T. et al., 2024; Yang et al., 2019; Zhang et al., 2020; Li S. et al., 2024; Yin et al., 2024). Notably, emerging evidence suggests that fucoidans play a crucial role in gut microbiota fermentation, thereby influencing lipid metabolism and atherosclerosis progression (Carrasqueira et al., 2025; Yin et al., 2024; Wei et al., 2022; Rashed et al., 2022). It is worth noting that the dose of fucoidan (100 mg/kg) used in this study was selected based on its optimal efficacy in ameliorating hyperlipidemia and inflammation in our prior dose-ranging investigation (Shi et al., 2025; Wang et al., 2025), allowing us to focus resources on elucidating the microbial and metabolomic underpinnings at this effective dosage.

Generally, *Firmicutes* and *Bacteroidota* exhibit positive and negative correlations with hyperlipidemia and atherosclerosis, respectively. The FH intervention increased the abundance of *p_Firmicutes*, aligning with findings in type 2 diabetic mice induced by HFD/streptozotocin and treated with *Sargassum fusiforme* fucoidan (Wu et al., 2021), but contrasting with the results from hyperlipidemic mice treated with *Laminaria digitata* fucoidan (Li S. et al., 2024). Notably, the Bacteroidetes-to-Firmicutes ratio is typically lower in type 2 diabetic mellitus than in healthy individuals (Larsen et al., 2010), and elevated *p_Firmicutes* levels may confer benefits in nonalcoholic steatohepatitis (Guo et al., 2018). The combined therapy increased the relative abundance of *p_Proteobacteria* and *p_Deferribacterota*, though their associations with hyperlipidemia remain inconsistent across studies (Li S. et al., 2024; Yang et al., 2023). Both the FSV and FH interventions enriched beneficial bacteria such as *c_Bacilli* and *c_Coriobacteriia* (Ma et al., 2022; Jin et al., 2023), while *Clostridia*—a class linked to nonalcoholic steatohepatitis progression in humans—was elevated by SV monotherapy but suppressed by the combined intervention (Lei et al., 2022). The SV intervention also potentially induced side effects by increasing *c_Negativicutes* and *c_Actinobacteria* (Pang et al., 2024). Specifically, the SV intervention group elevated the abundance of *o_Oscillospirales*, *o_Lactobacillales*, *f_Oscillospiraceae*, *f_Lachnospiraceae*, *g_Clostridia_UCG-014*, *g_NK4A214_group*, and short-chain fatty acid producing *f_Christensenellaceae* and *g_Bifidobacterium*, while reducing the abundance of *o_Erysipelotrichales* and *f_Erysipelotrichaceae*, collectively ameliorating hyperlipidemia and atherosclerosis (Guo et al., 2018; Ahrens et al., 2021; Duan et al., 2024; Song et al., 2023; Gao et al., 2024; Zhang et al., 2021; Jia et al., 2021; Li S. Q. et al., 2024). Conversely, the FH intervention might ameliorate hyperlipidemia and bile acid metabolism by enhancing the relative abundance of *g_Turicibacter* (Lynch et al., 2023). The combined therapy further upregulated lipid-lowering and bile acid producing taxa (*f_Enterobacteriaceae*, *g_Escherichia-Shigella*, *g_Citrobacter*, and *g_Enterococcus*) (Jia et al., 2021; Xiong et al., 2022; Wang et al., 2021), and increased the abundance levels of beneficial species as shown in Figure 8 (Liu Q. et al., 2024; Sun et al., 2022; Hughes et al., 2022). A recent study by Liu et al. (2025) demonstrated that *E. coli* Nissle 1917 attenuates atherosclerosis in apolipoprotein E-deficient mice by regulating HFD-induced disruptions of the gut microbiota and serum metabolites. This finding raises the possibility that the enriched *E. coli* may also mediate, at least in part, the anti-atherosclerotic effects of fucoidan. The rise in *g_Enterococcus* may specifically reflect fucoidan’s influence, as observed in *Sargassum fusiforme* fucoidan-treated mice (Wu et al., 2021). However, these altered bacterial taxa may exhibit context-dependent effects or serve merely as markers of dysbiosis rather than direct causal agents. For instance, *Escherichia-Shigella* can produce trimethylamine, a precursor of trimethylamine N-oxide (TMAO) (Lu et al., 2023). Similarly, the prevalence of gut *Enterococcus* and elevated abundance of oral *Escherichia-Shigella* have been identified as potential biomarkers of Kawasaki disease (Zeng et al., 2023), whereas *Enterococcus* is associated with disease severity and poor prognosis in COVID-19 (Farsi et al., 2022). Additionally, *Enterococcus* is recognized as a potential pathobiont in common variable immunodeficiency with immune dysregulation (Berbers et al., 2025). In patients with liver disorders, increased abundances of *Enterobacter*, *Enterococcus*, and *Clostridium* have been observed, which are associated with elevated levels of secondary bile acids (Jia et al., 2018). Moreover, the *Enterobacteriaceae*/*Escherichia-Shigella* group is enriched in patients with atherosclerotic cardiovascular disease (Jie et al., 2017; Zhang et al., 2025). Notably, numerous studies have documented the relationship between gut microbiota dysbiosis and atherosclerosis (Tonch-Cerbu et al., 2025; Sun et al., 2022; Shen et al., 2021; Brown et al., 2023).

The microbiota ameliorates dyslipidemia and atherosclerosis primarily through metabolite production. Branched-chain amino acids (valine, leucine, and isoleucine) share metabolic pathways and promote atherosclerosis, whereas L-Arginine exerts beneficial effects (Wu et al., 2024). Elevated alanine metabolism and aromatic amino acids (phenylalanine, tyrosine, and tryptophan), show positive correlations with CVD events (Deidda et al., 2021; Botello-Marabotto et al., 2025). Additionally, gut microbial metabolism of PC accelerates atherosclerosis by generating choline, trimethylamine, and trimethylamine N-oxide (Wang et al., 2011). While the SV intervention modulated these harmful amino acids and choline pathways inconsistently (Table 2), it potentially benefited atherosclerosis through enhanced ABC transporters. The observed downregulation of glycerophospholipid metabolism may further contribute to simvastatin’s anti-atherosclerotic effects (Su et al., 2022). However, SV intervention promoted ALS-related pathway (Bai et al., 2021), revealing a potential adverse effect. In contrast, both FSV and FH interventions significantly enhanced primary bile acid biosynthesis, suggesting that *F. vesiculosus*-derived fucoidan may promote reverse cholesterol transport. The FSV intervention group particularly modulated alpha-linolenic acid metabolism and serotonergic synapse while downregulating fatty acid biosynthesis/elongation through reduced palmitic and stearic acid levels (Table 2), which may contribute to the reduced atherosclerotic plaque formation (Winnik et al., 2011; Duan et al., 2021; Shen et al., 2025). The pentose and glucuronate interconversions pathway exhibited stage-dependent atherogenic effects (Wang et al., 2023), with SV and FSV interventions exerting opposing modulations. Notably, the FH treatment upregulated the sphingolipid signaling pathway via increased sphinganine (Table 2), implying atherosclerosis protection through HDL-bound sphinganine-1-phosphate generation (Guo et al., 2014).

Collectively, this study demonstrated that combined *F. vesiculosus* fucoidan and simvastatin significantly attenuated atheroma formation in New Zealand rabbits with HFD-induced hyperlipidemia and balloon catheter injury, a model exhibiting pathological characteristics similar to human atherosclerosis. While our prior work focused on lipid disorder and systemic inflammation (Shi et al., 2025; Wang et al., 2025), the present findings indicate that the therapy’s benefits may be linked to a beneficial remodeling of the gut microbiota and its metabolic outputs. These findings in non-genetically modified rabbits—a model mirroring the characteristics of hyperlipidemic patients with atherosclerosis—hold significant translational value for fucoidan’s clinical application. However, several limitations warrant consideration. First, while both fucoidan alone and combined therapy showed multi-target effects on lipid metabolism and gut microbiota-related pathways, unmeasured plasma metabolite levels prevent definitive identification of primary anti-atherosclerotic mechanisms. Second, while the anti-inflammatory properties are established (Shi et al., 2025), the specific link between the regulated gut metabolites and inflammatory pathways within the arterial wall remains unclear and requires future investigation. Third, given that most of the results in this study were derived from exploratory omics experiments and KEGG pathway analysis without FDR adjustment, further investigation is required to determine whether co-administration of fucoidan reduces simvastatin-associated side effects, as well as the extent to which this combination enhances the anti-atherosclerotic efficacy of simvastatin. Fourth, the exclusive use of male rabbits, while unlikely to reflect gender-specific treatment effects, necessitates validation in female models to comprehensively establish therapeutic efficacy. Fifth, while randomization was performed using Excel functions and double blinding was maintained during the analysis of gut microbiota and metabolites, no blinding was applied during treatment administration or during the assessment of the lipid profile and Oil Red O staining. This may have introduced bias, as previously stated (Shi et al., 2025).

## 5 Conclusion

While our previous research established the foundational efficacy and anti-inflammatory profile of this combination (Shi et al., 2025; Wang et al., 2025), the current work uncovers a deeper mechanistic layer by identifying specific microbial taxa and host-microbial co-metabolites associated with the enhanced therapeutic effects. Using an HFD-fed, balloon catheter-injured rabbit model—a system with superior translational relevance due to its human-like hyperlipidemic profile and reliable lesion formation—we demonstrate that combined *F. vesiculosus* fucoidan and simvastatin therapy attenuates atherosclerosis. Based on the present KEGG pathway analysis, the combination may mitigate simvastatin-induced side effects, potentially through modulation of the gut microbiota and its metabolome—a mechanism that warrants further investigation. Nevertheless, our findings suggest that these benefits are partially mediated by a beneficial remodeling of gut microbial communities and their associated metabolic outputs. Furthermore, this work identifies that the gut microbiota-metabolite axis as a promising therapeutic target and elucidates a mechanism by which fucoidan might enhance the efficacy and tolerability of statin therapy, paving the way for novel multi-targeted strategies in atherosclerosis management.

## Data Availability

The data presented in the study are available in the CNCB-NGDC Genome Sequence Archive repository, accession number CRA044158.

## Footnotes

1
https://spssau.com/indexs.html
