# Supplementary material for: Fucus vesiculosus fucoidan alone and in combination with simvastatin is associated with both alleviation of atherosclerosis and modulations in the gut microbiota and its metabolites in New Zealand rabbits
Source: Front Microbiol. 2026 Jun 18;17:1768989. doi: 10.3389/fmicb.2026.1768989 (PMC13323129; doi:10.3389/fmicb.2026.1768989)
Supplement: Supplementary file 1 [file Data_Sheet_1.ZIP › Supplementary Figures.docx]

**Supplementary Figures**

**Note: SFUC means FSV; FUC means FH.**


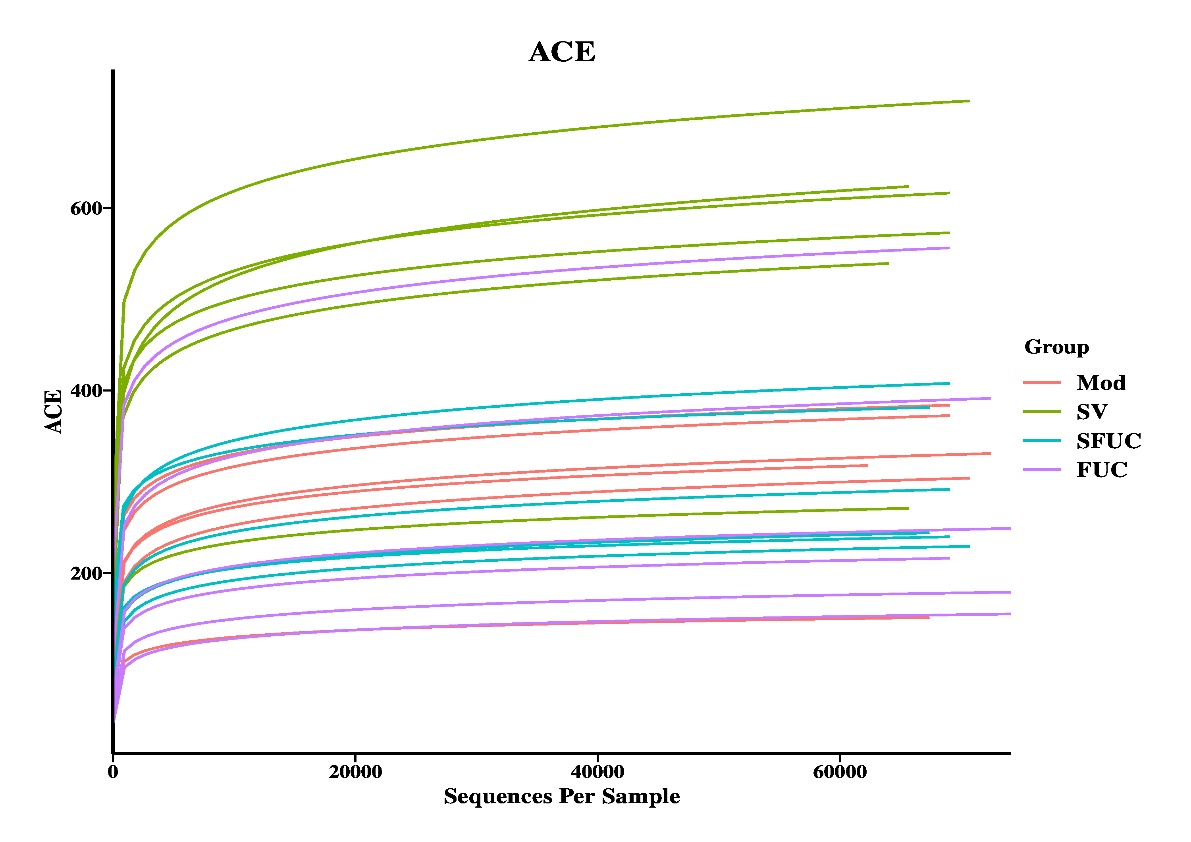


1. ACE


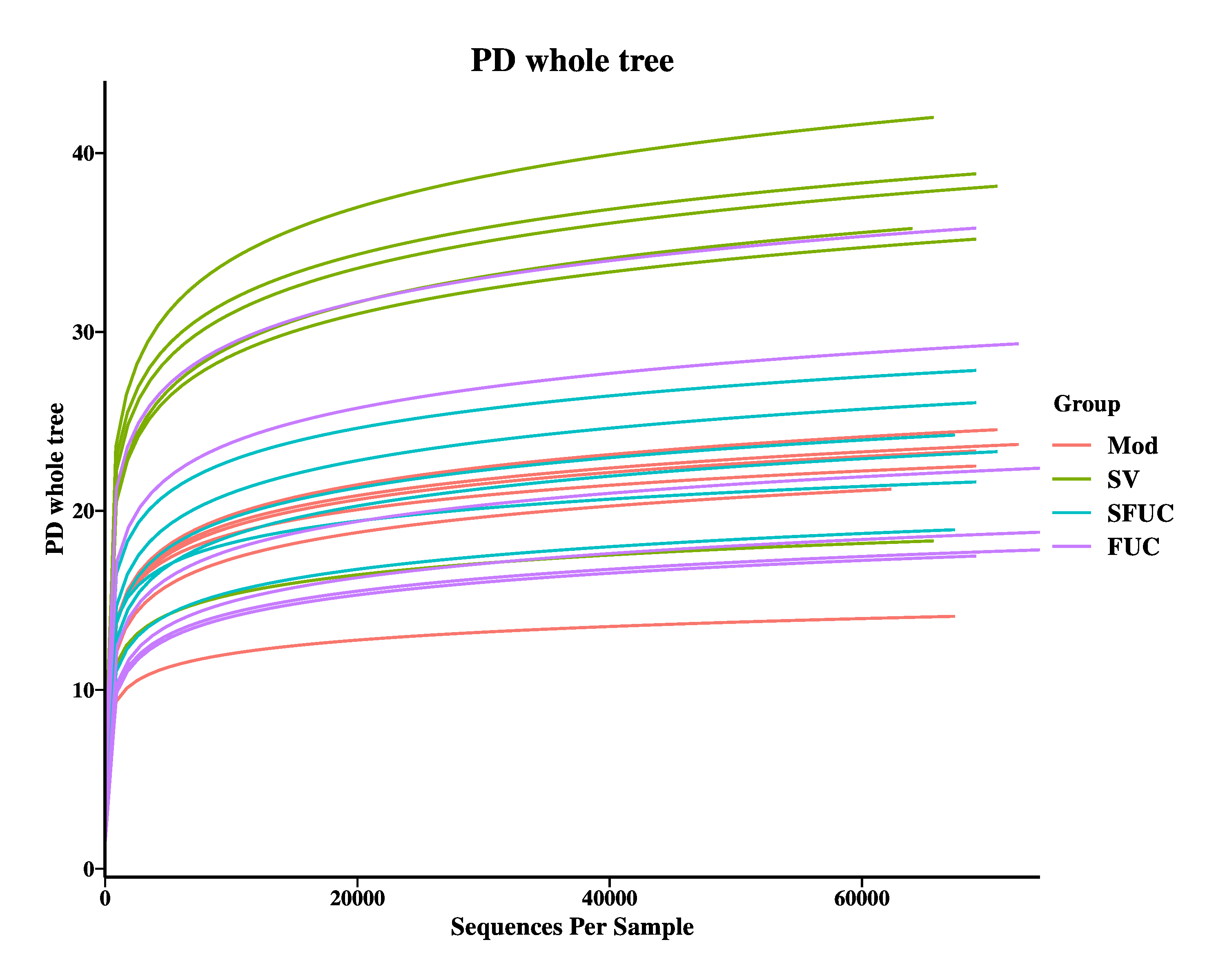


1. **PD whole tree**


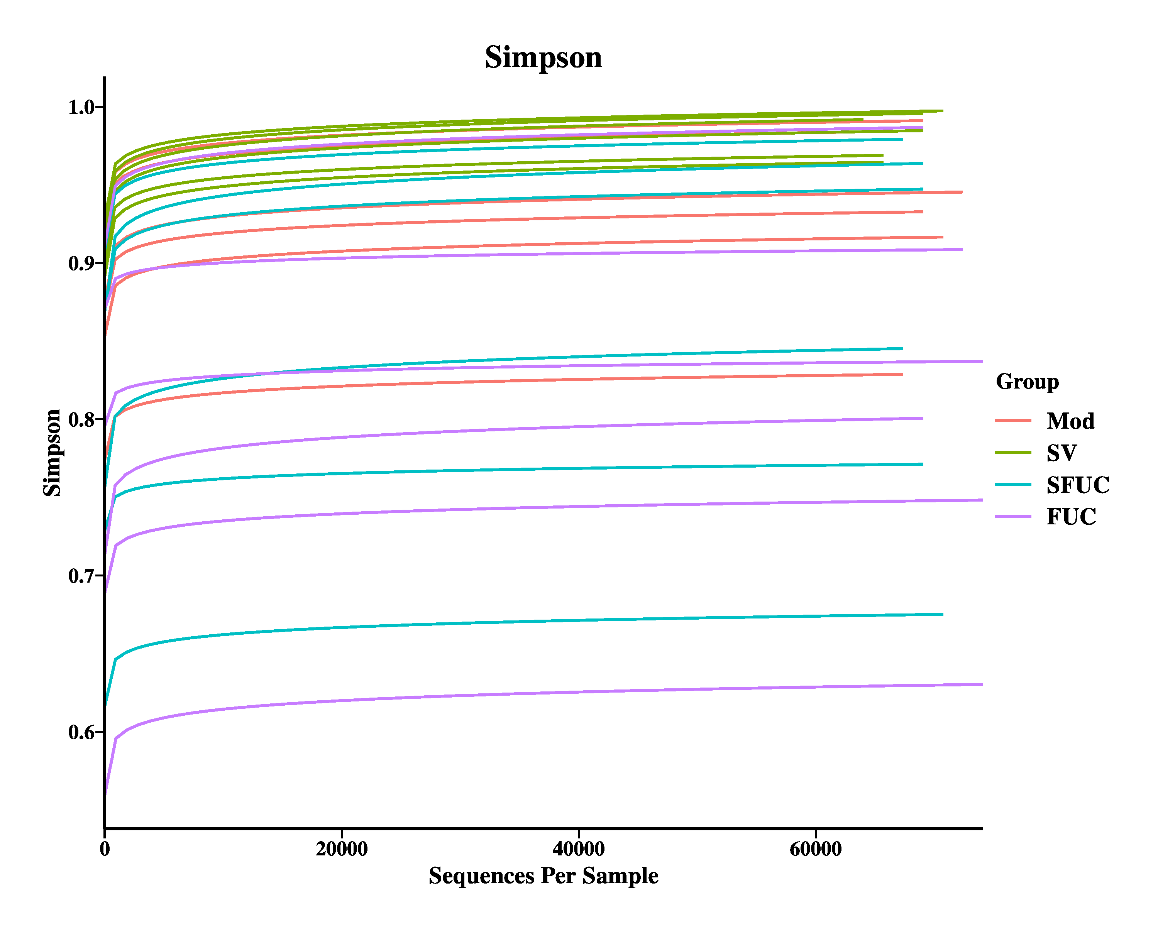


1. **Simpson**


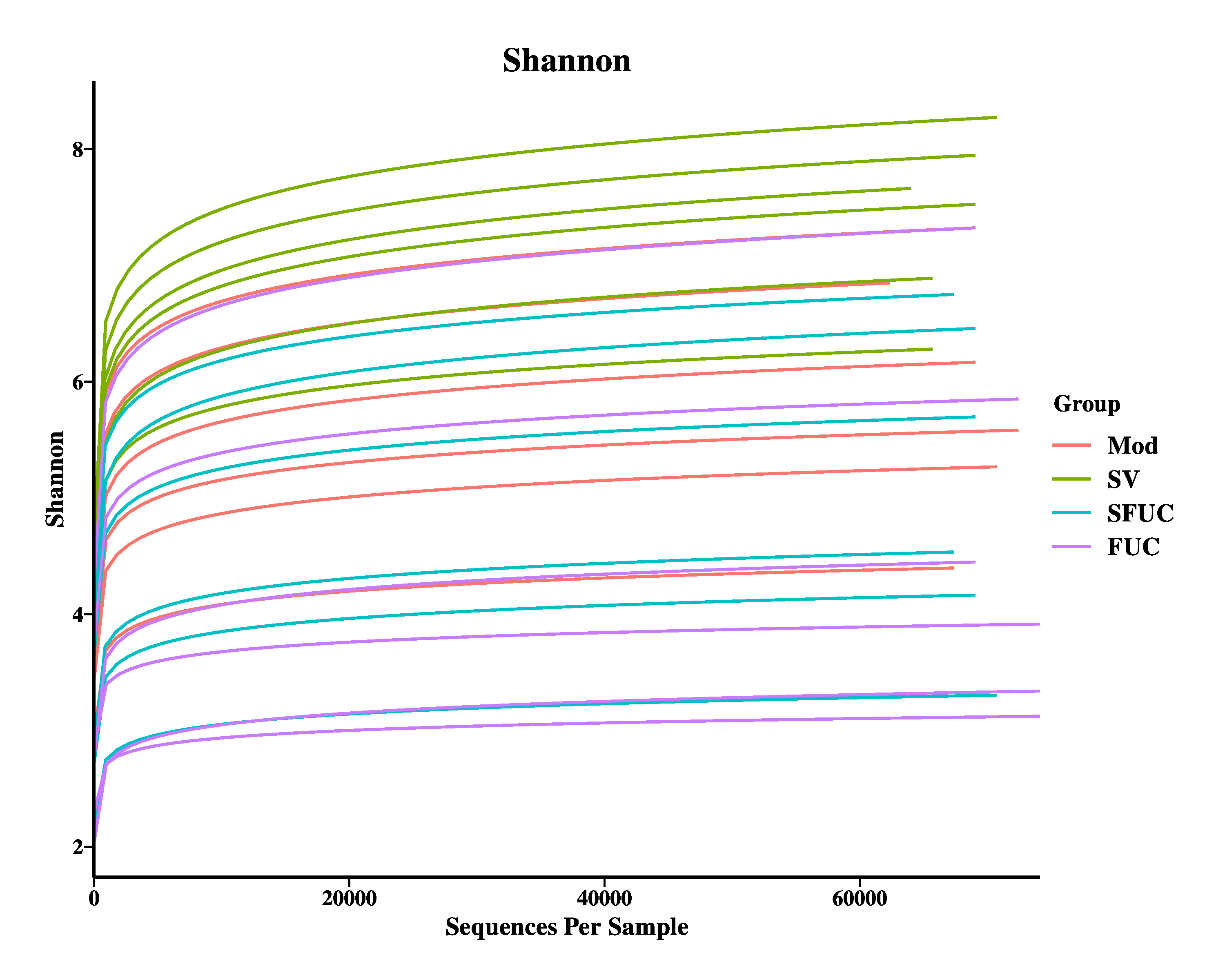


1. **Shannon**

**Supplementary Figure 1.** Rarefaction curves for ASVs and alpha diversity indices.


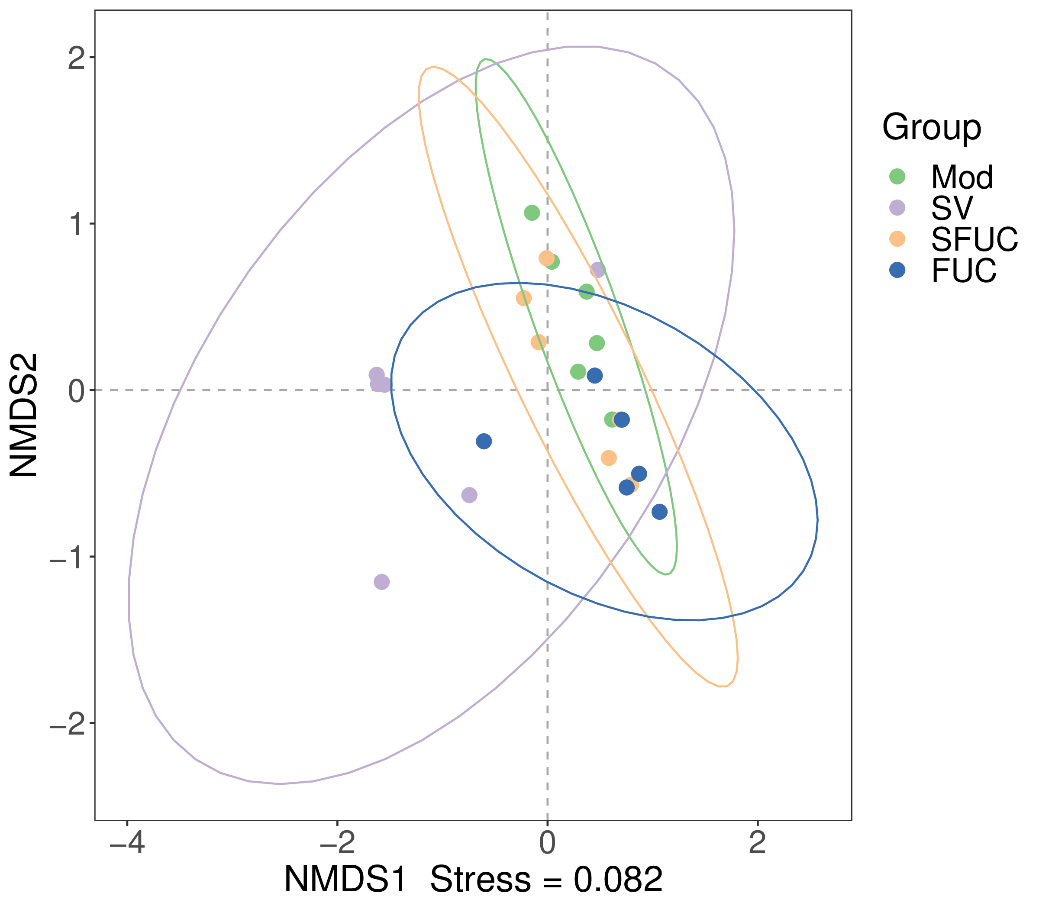


1. NMDS Bray–Curtis


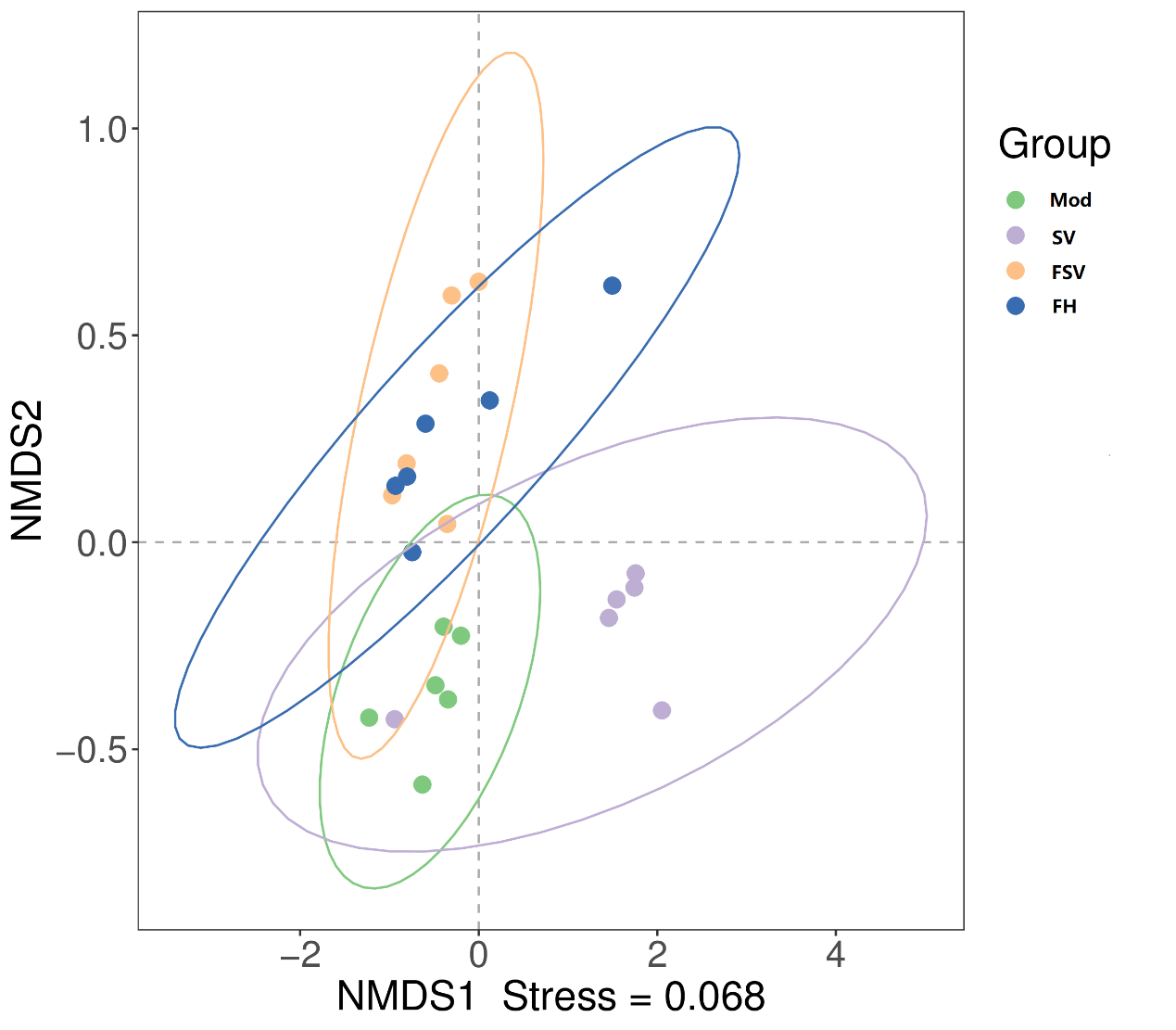


1. NMDS: Unweighted unifrac


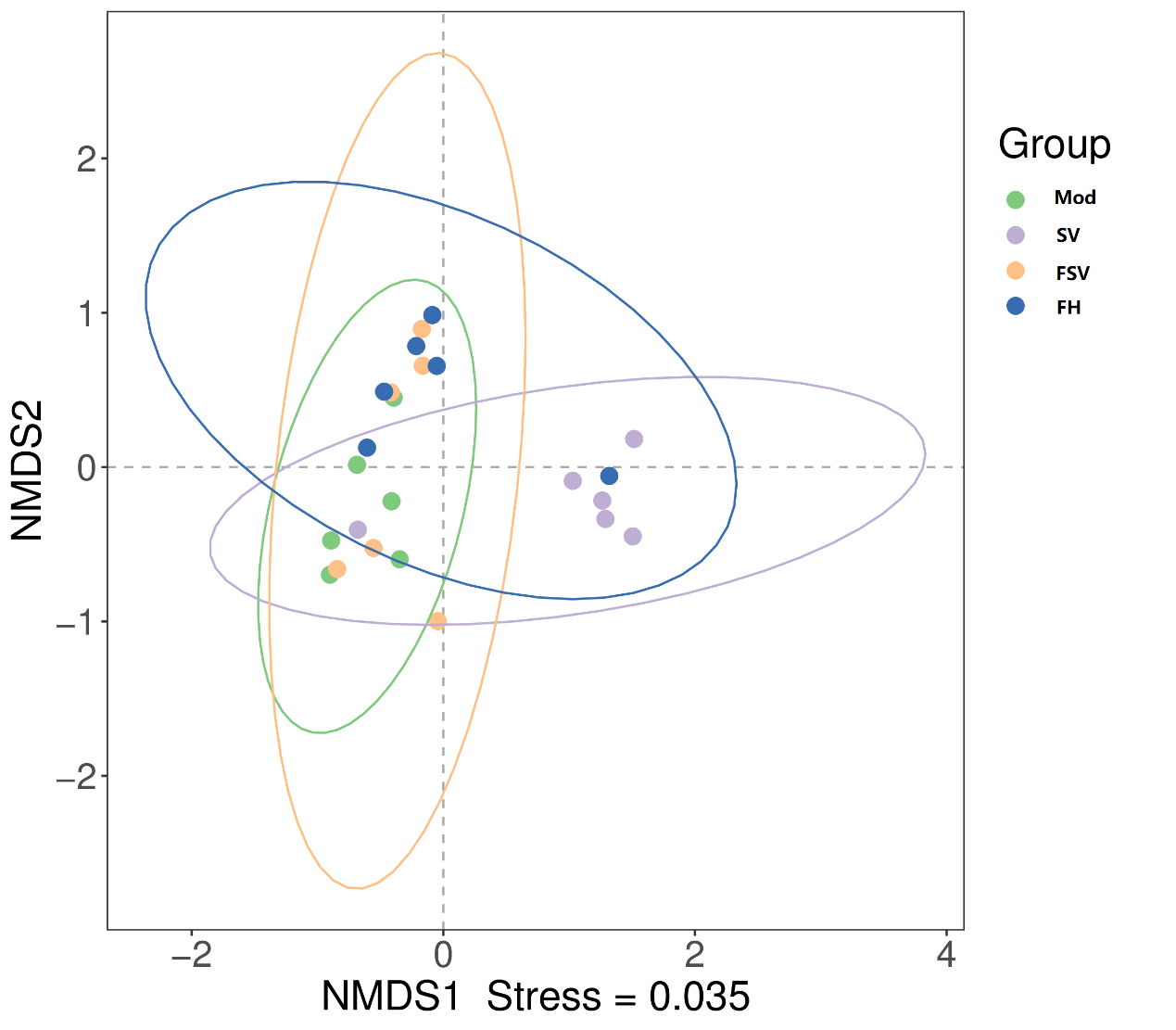


1. NMDS: Weighted unifrac


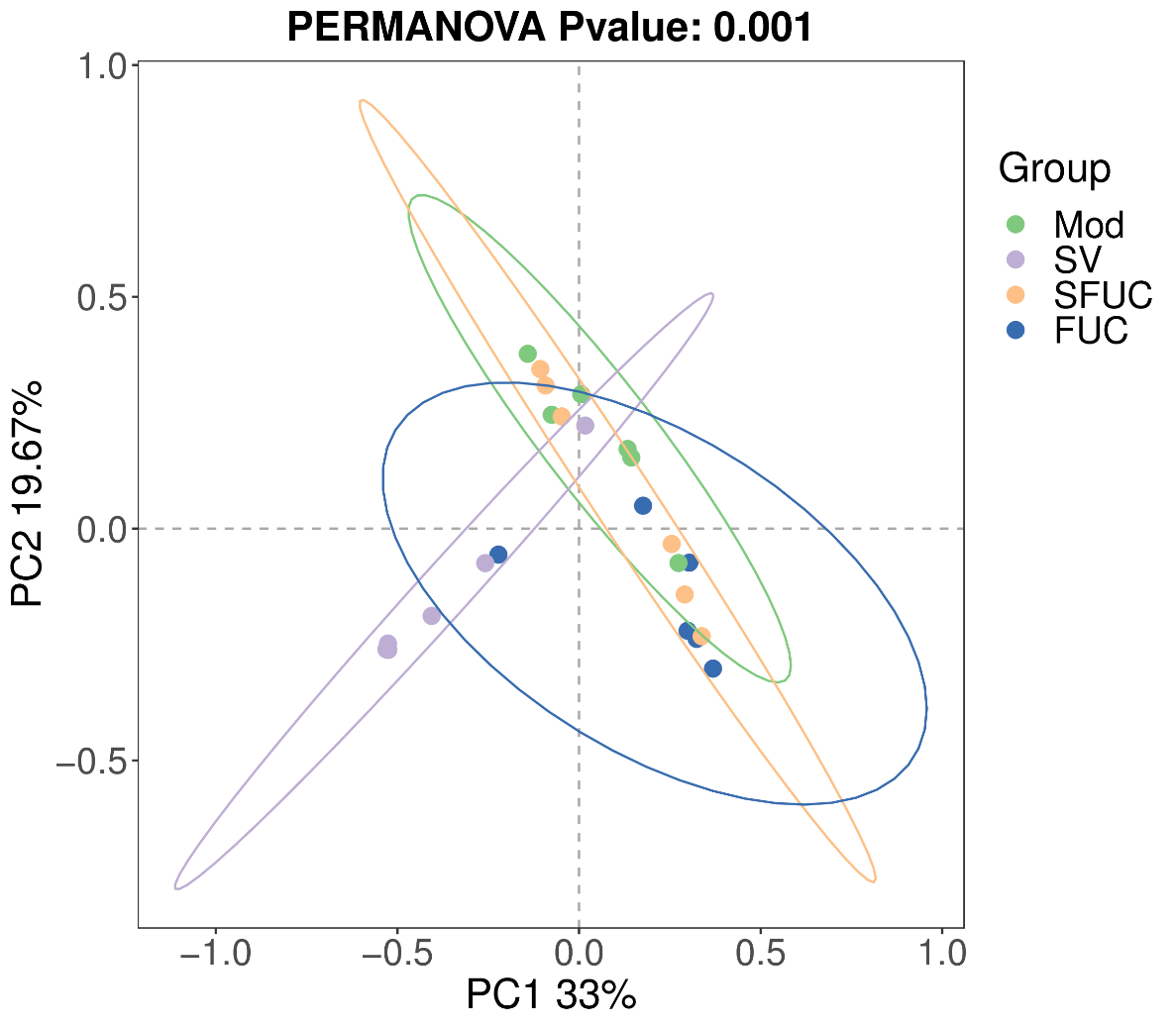


1. PcoA Bray-Curtis


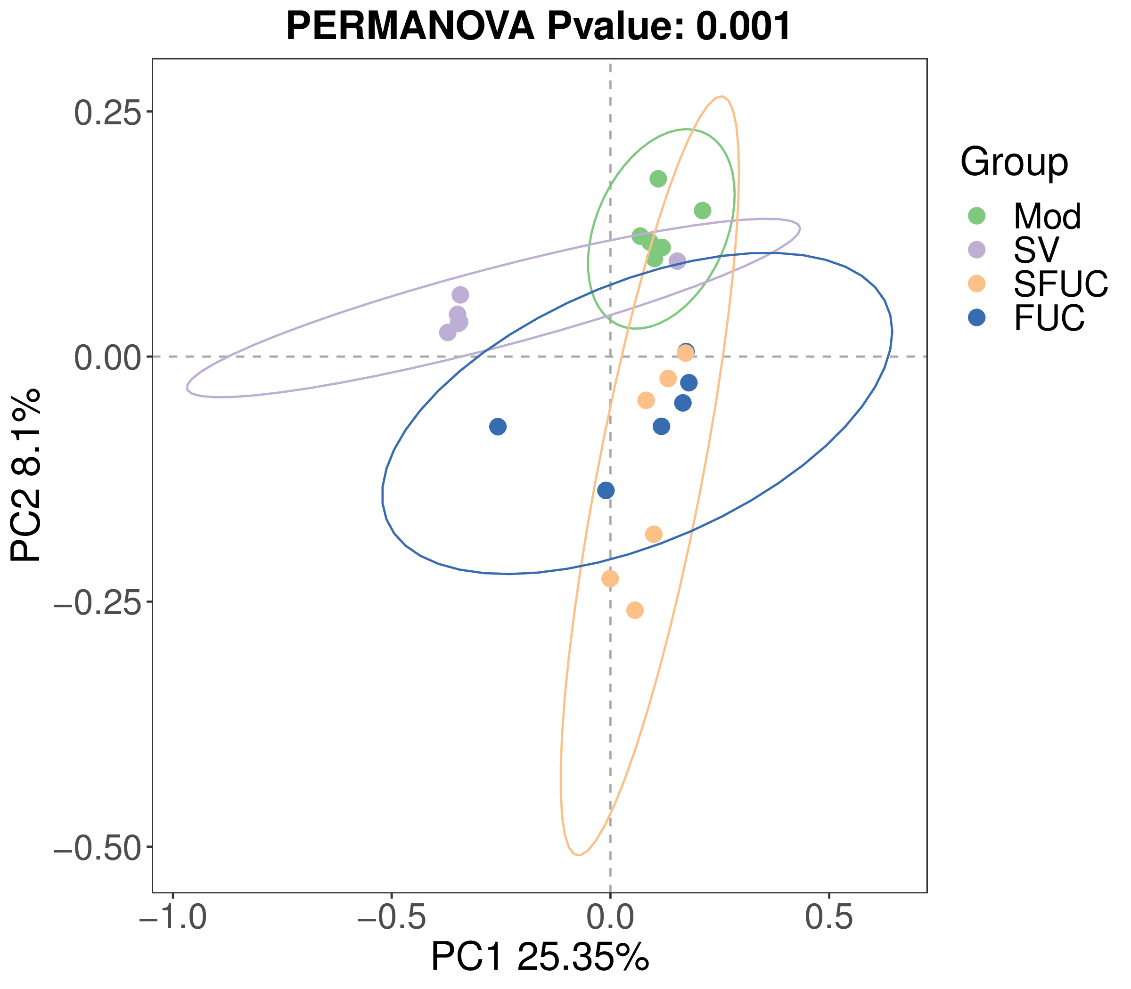


1. PcoA Unweighted Unifrac


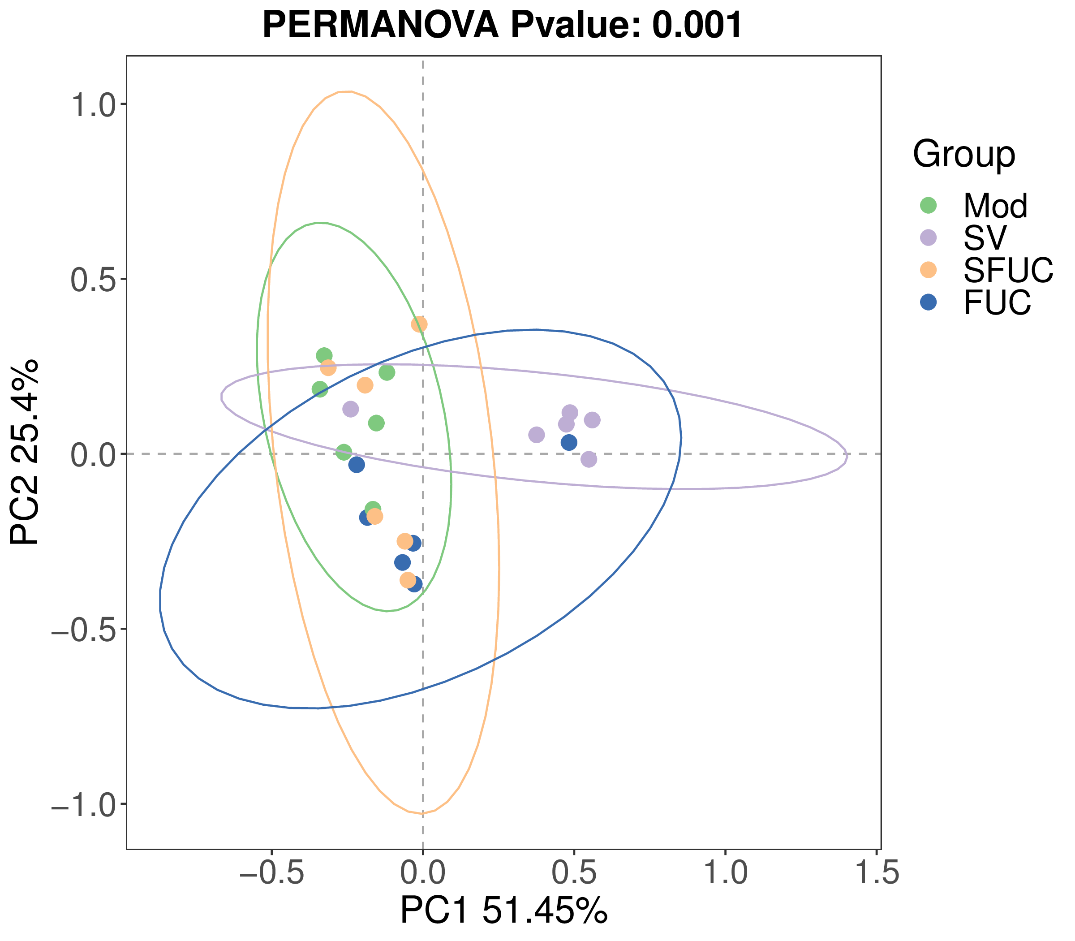


1. PcoA Weighted unifrac

**Supplementary Figure 2.** Beta-diversity testing.


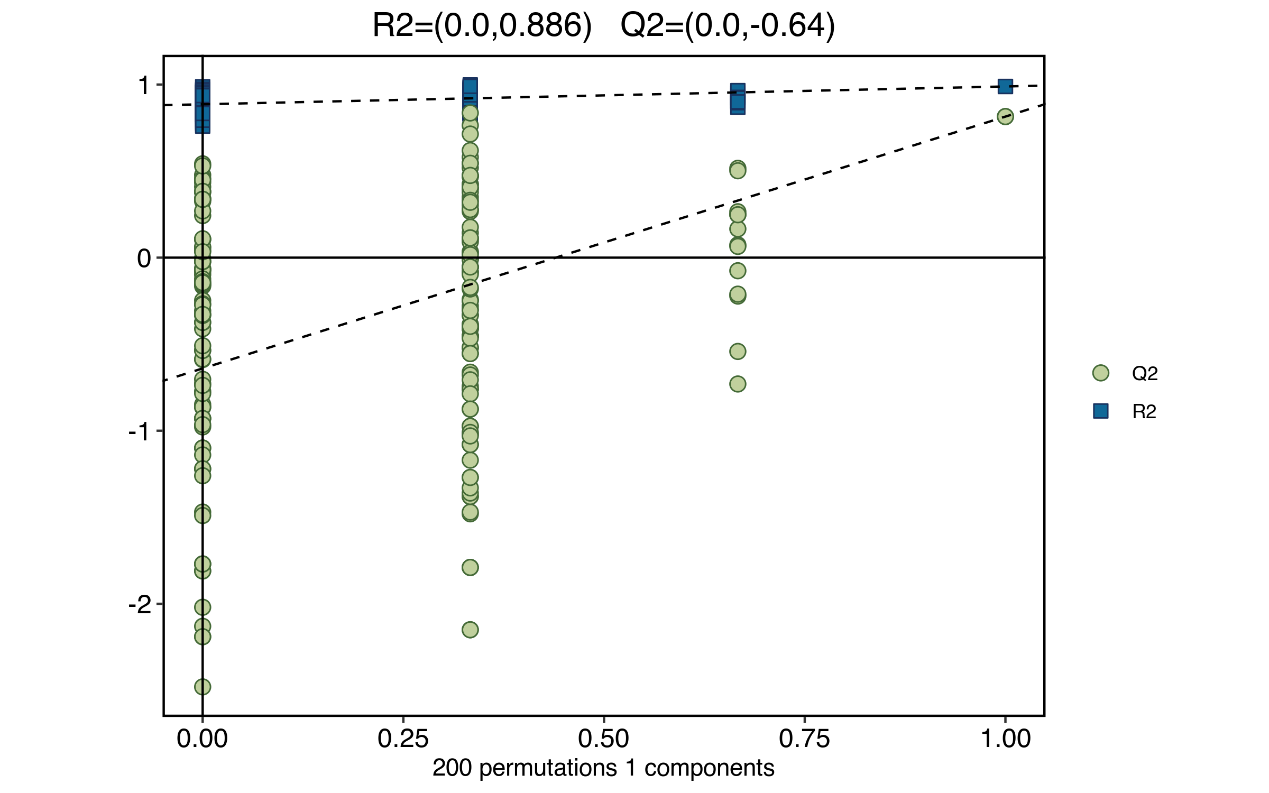


**A**. Permutation-FH-vs-Mod.


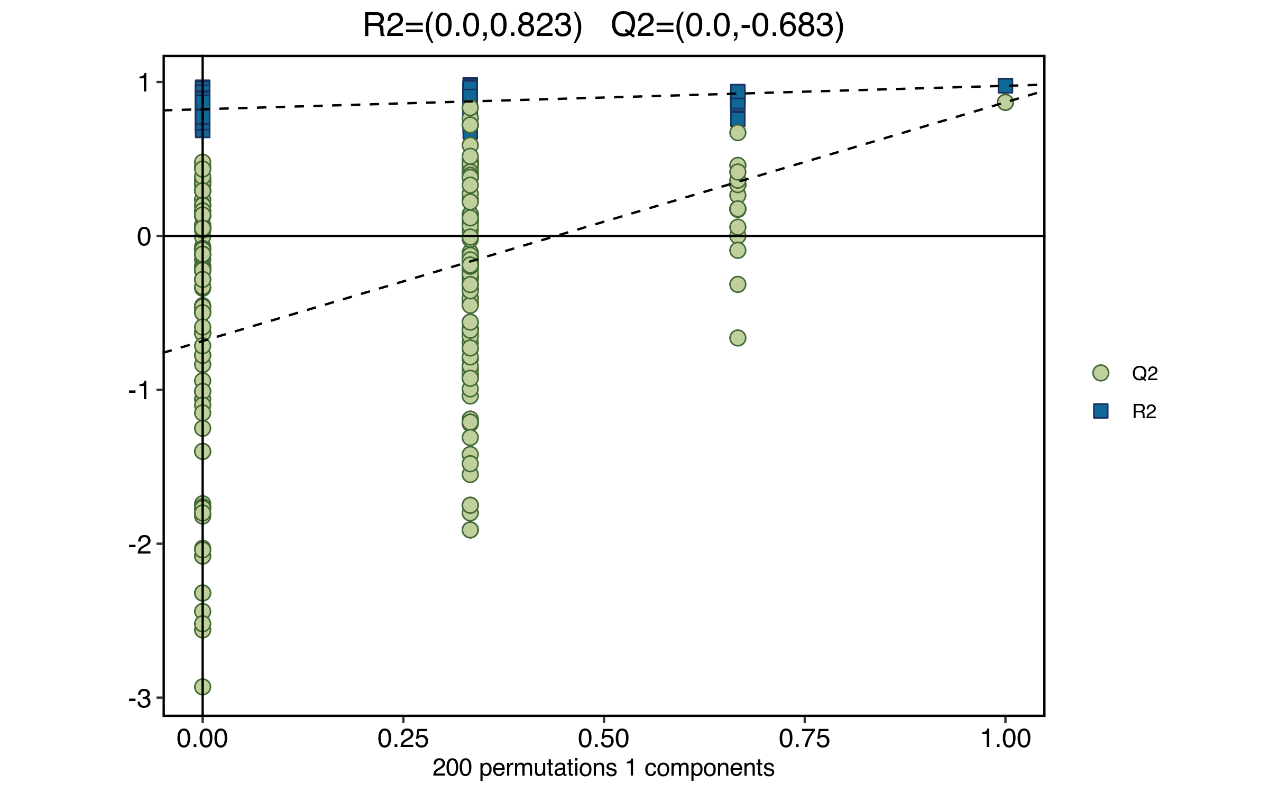


**B.** Permutation-SV-vs-Mod.


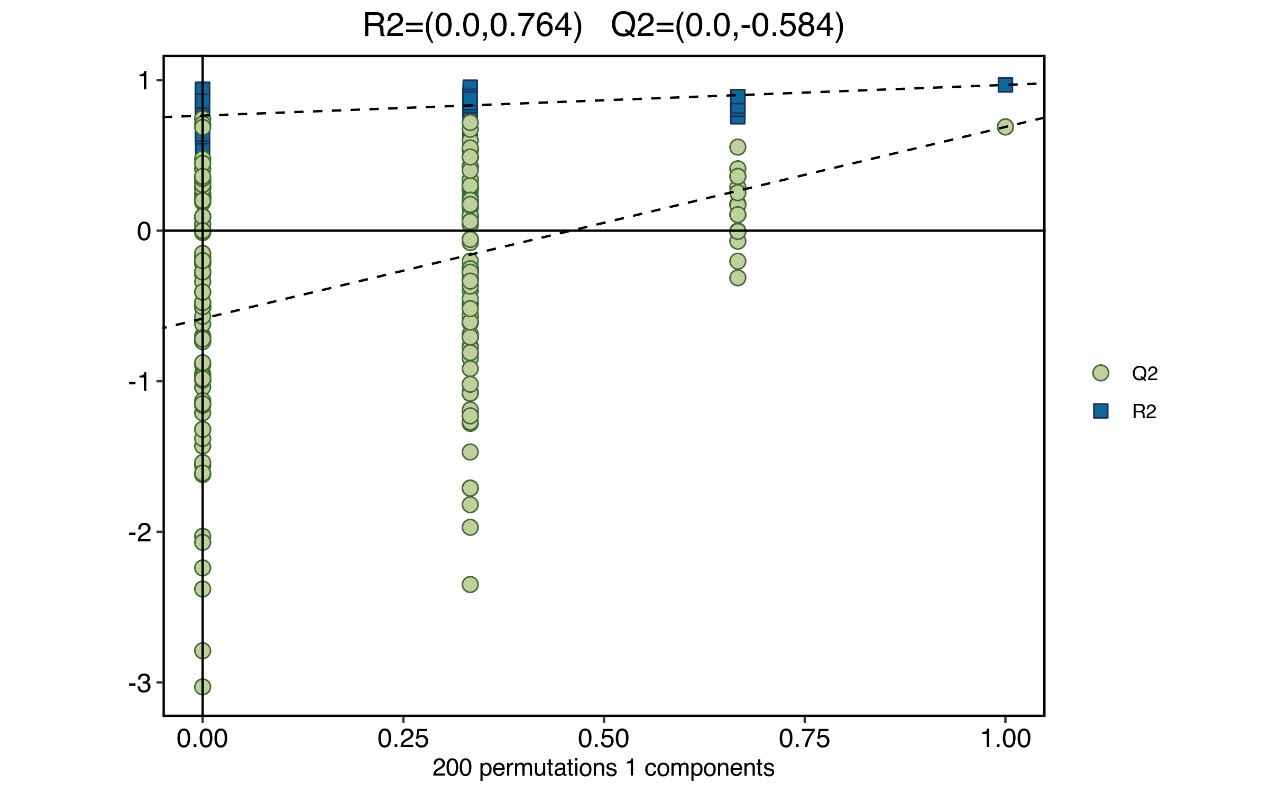


**C.** Permutation-FSV-vs-Mod.

**Supplementary Figure 3.** **Permutation testing of LC-MS/MS data from gut microbial metabolites.**

**
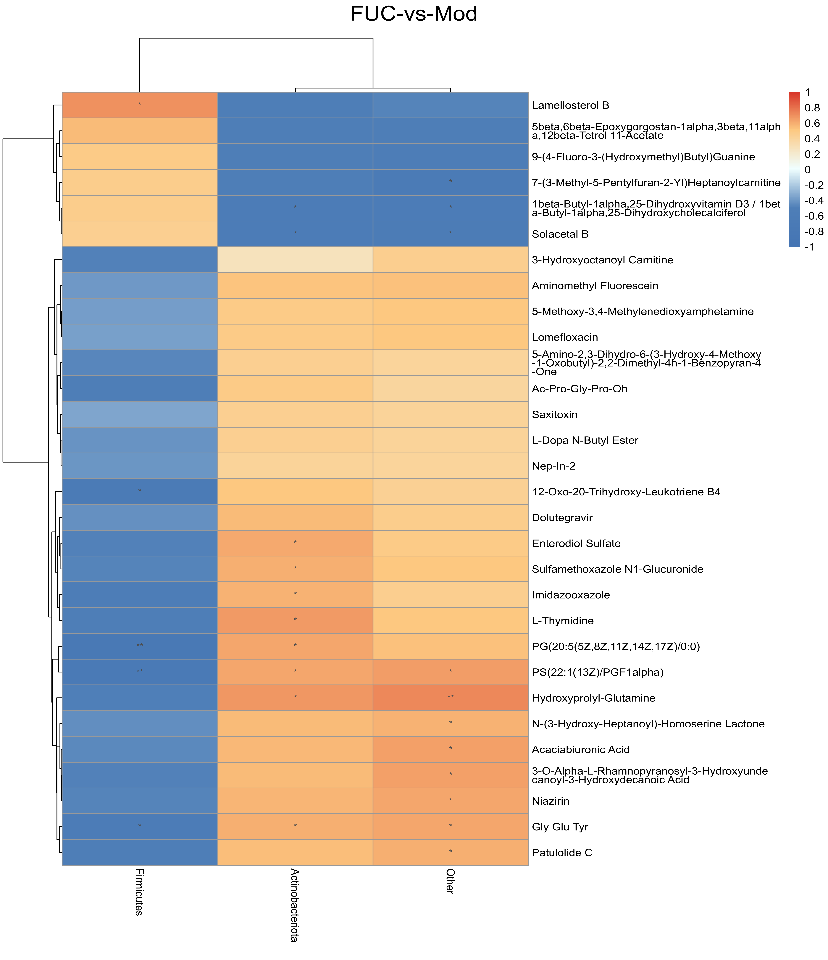
**

**A. Fuc vs. Mod at the Phylum level.**

**
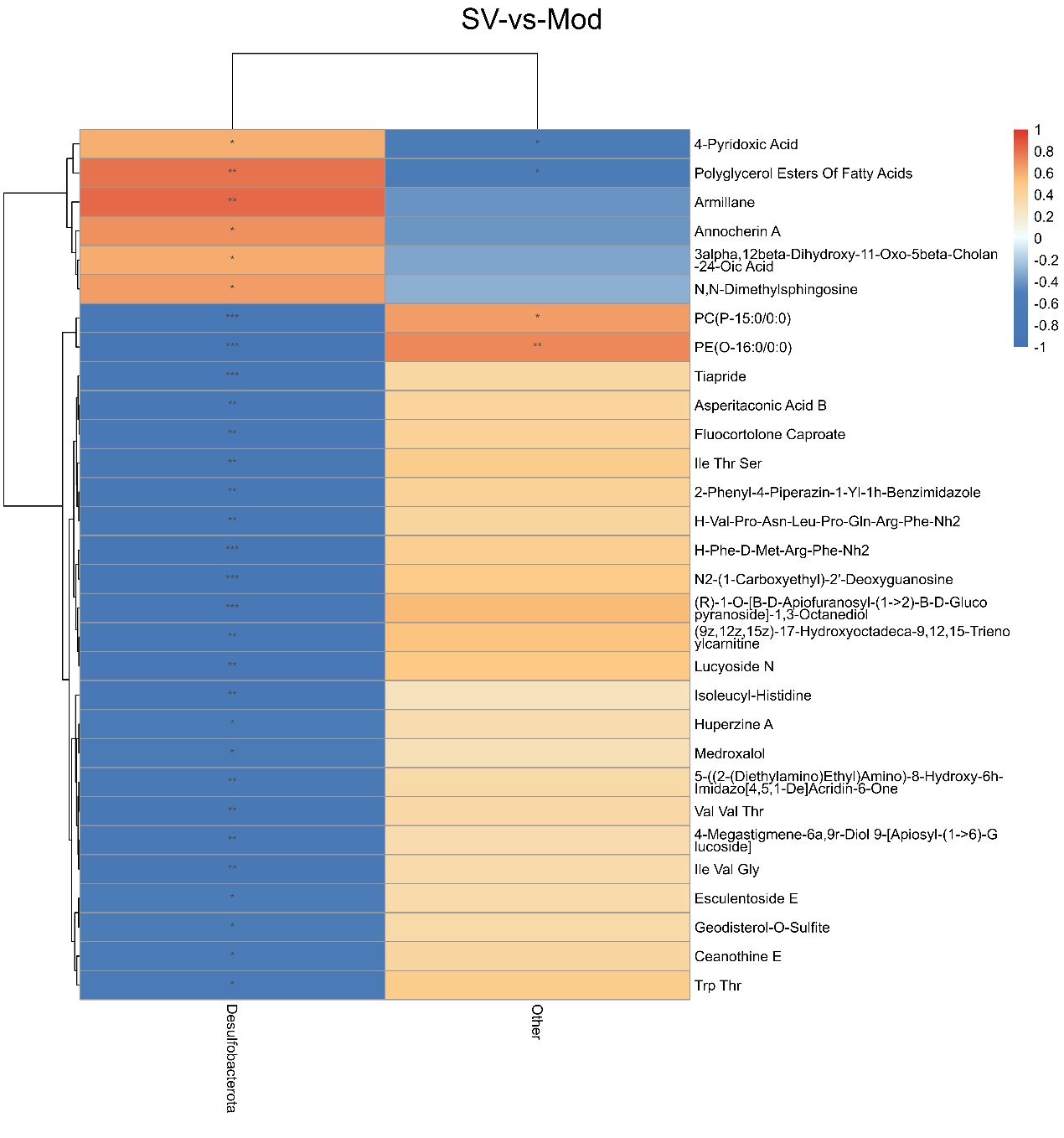
**

**B. SV vs. Mod at the Phylum level.**

**
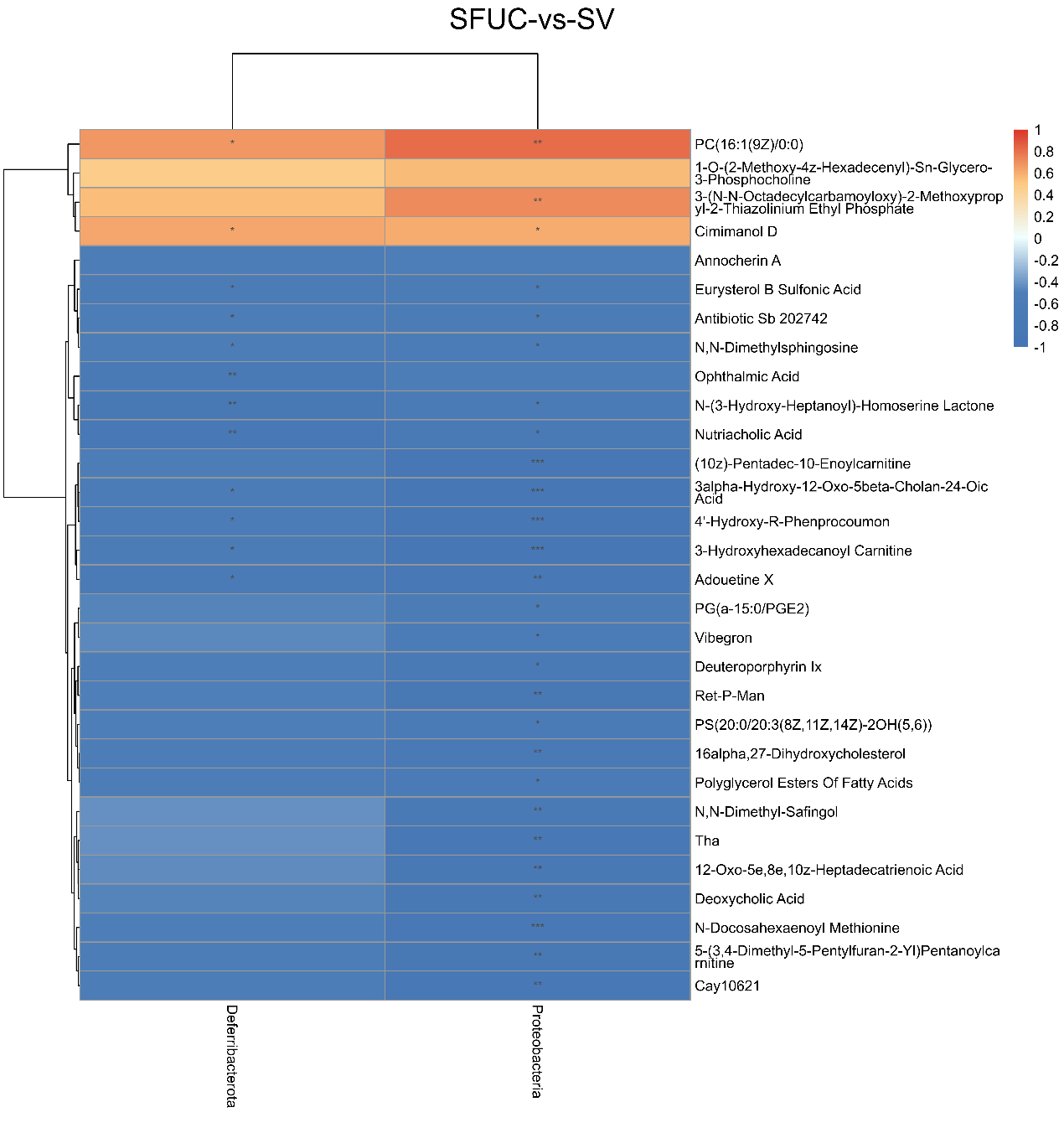
**

**C. SFuc vs. SV at the Phylum level.**

**
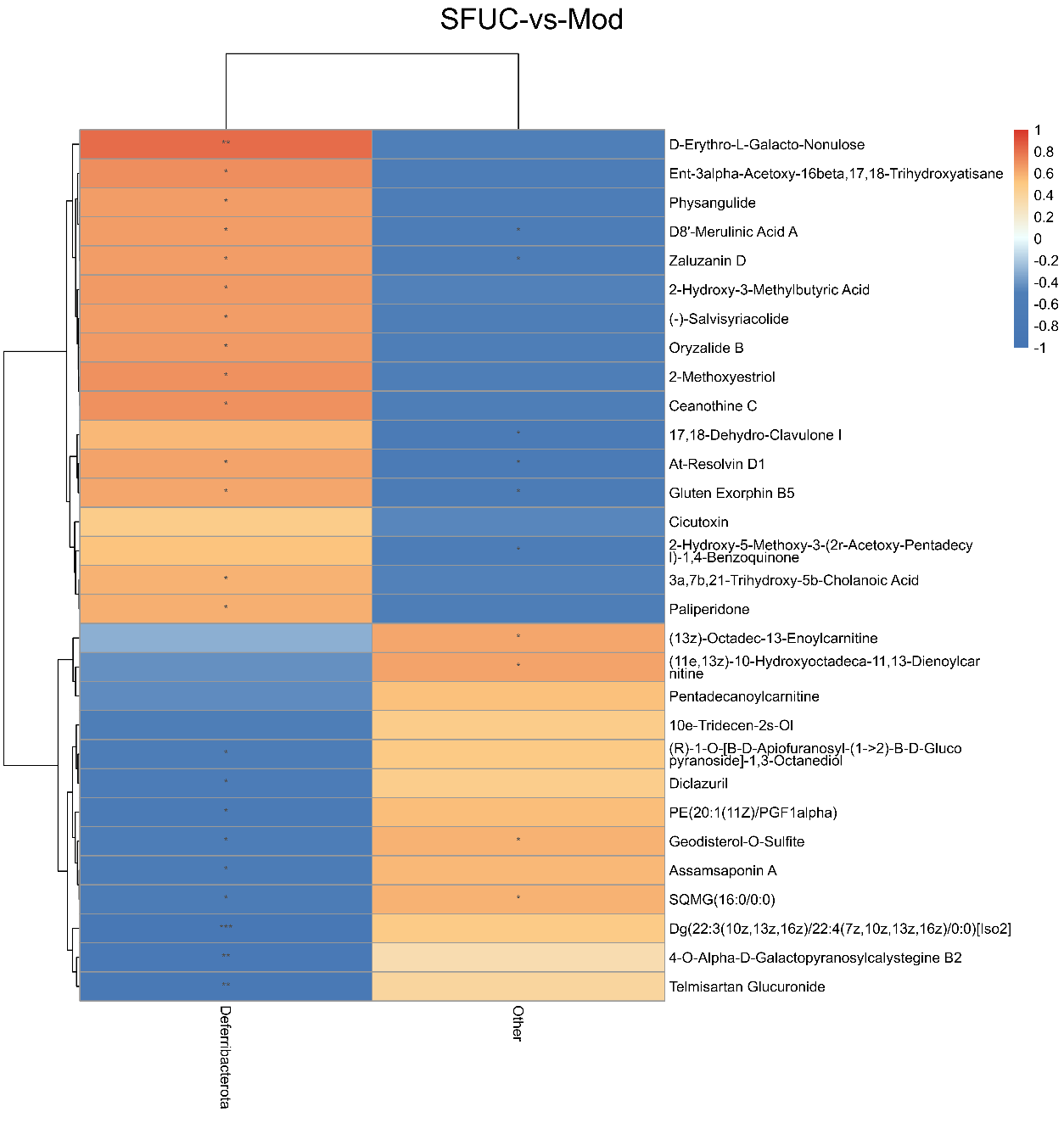
**

**D. SFuc vs. Mod at the Phylum level.**

**
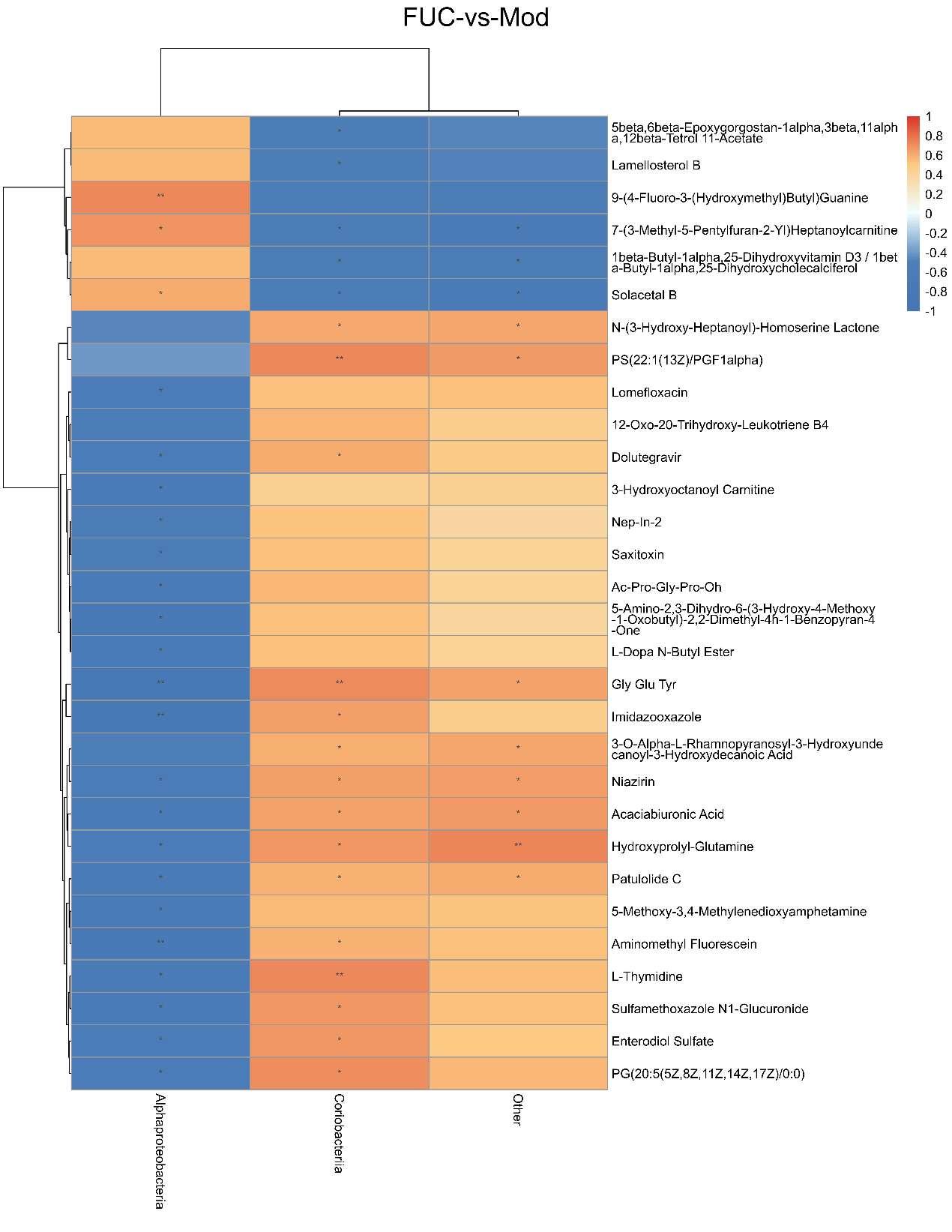
**

**E. Fuc vs. Mod at the Class level.**

**
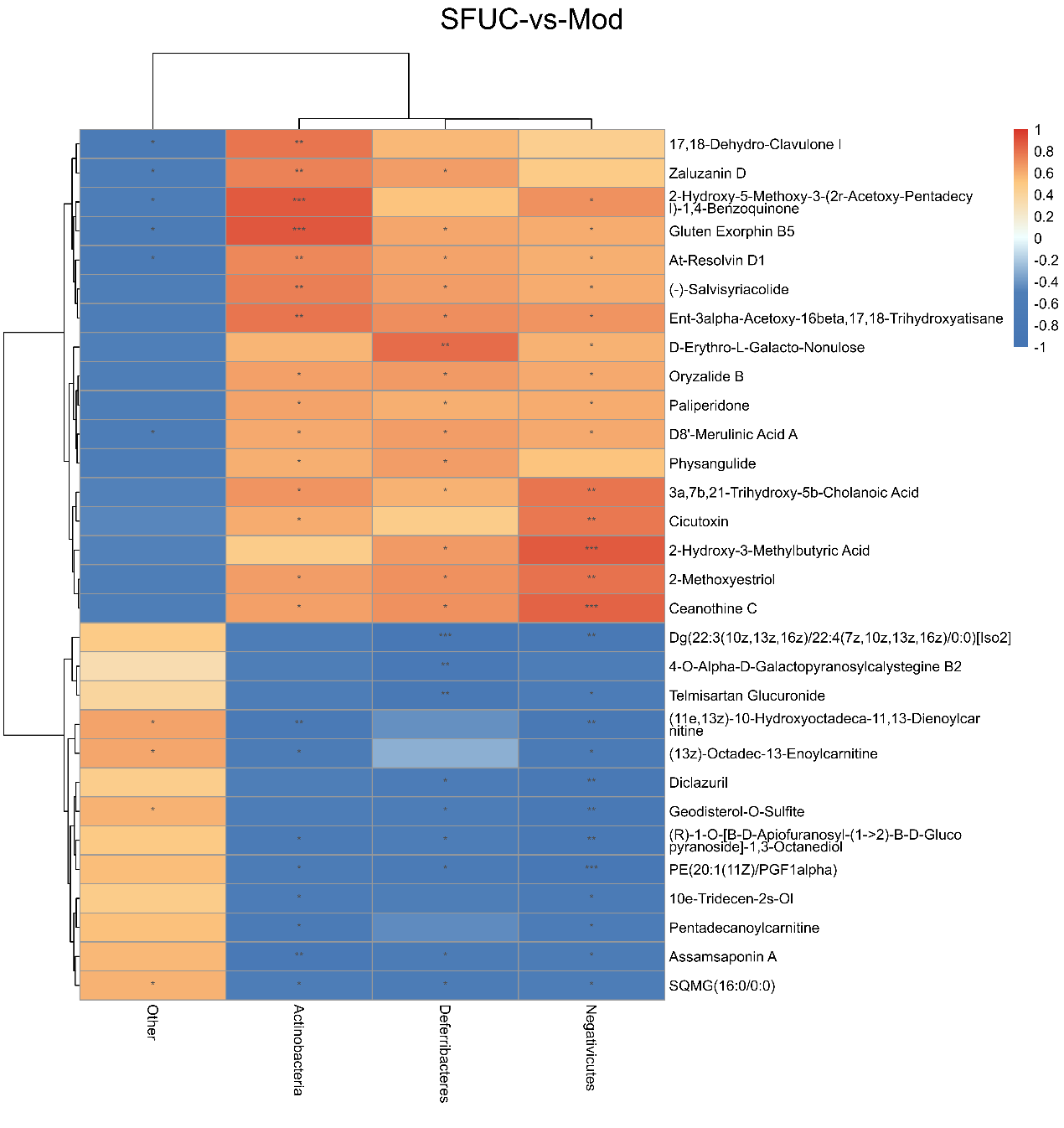
**

**F. SFuc vs. Mod at the Class level.**

**
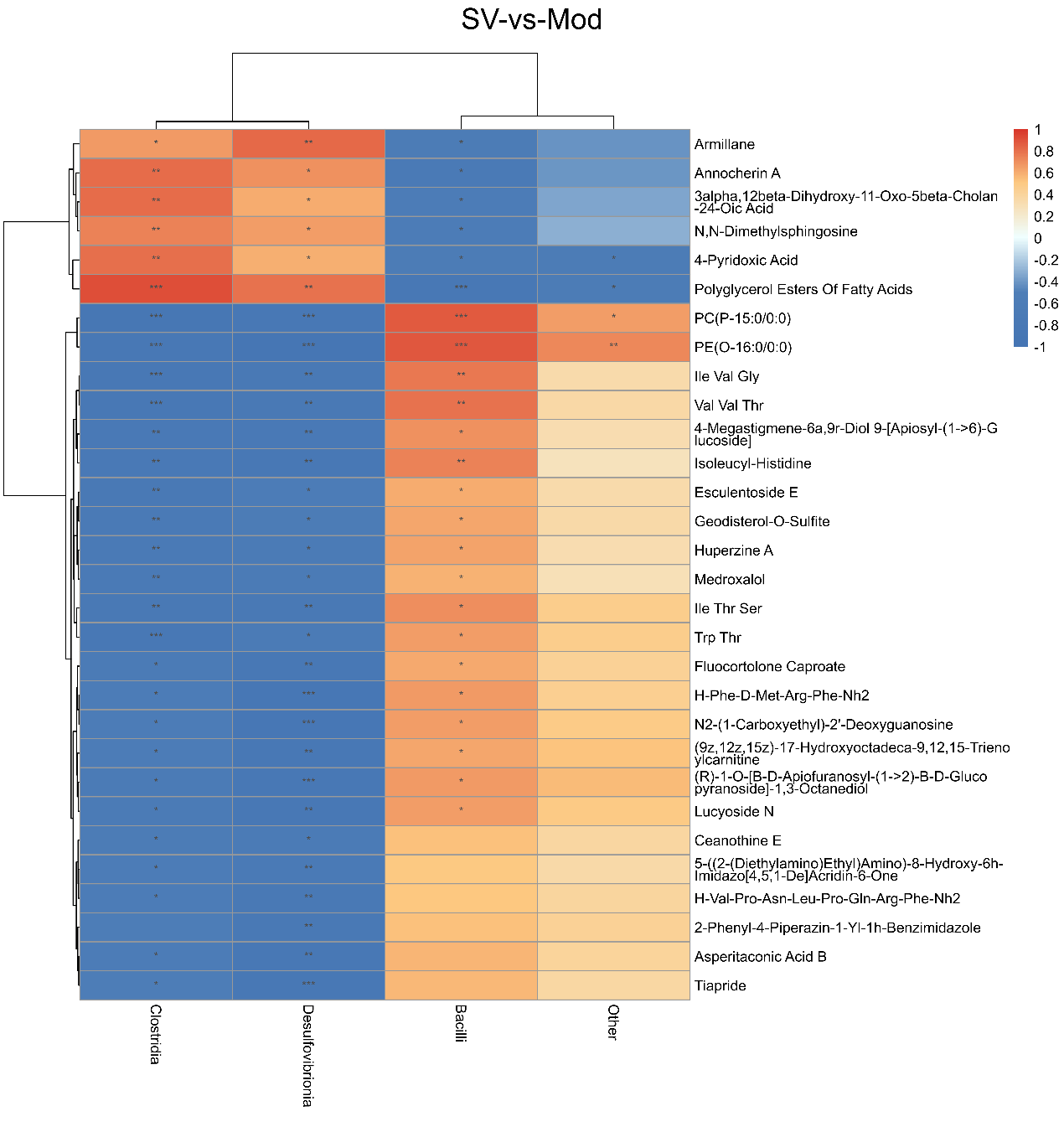
**

**G. SV vs. Mod at the Class level.**

**
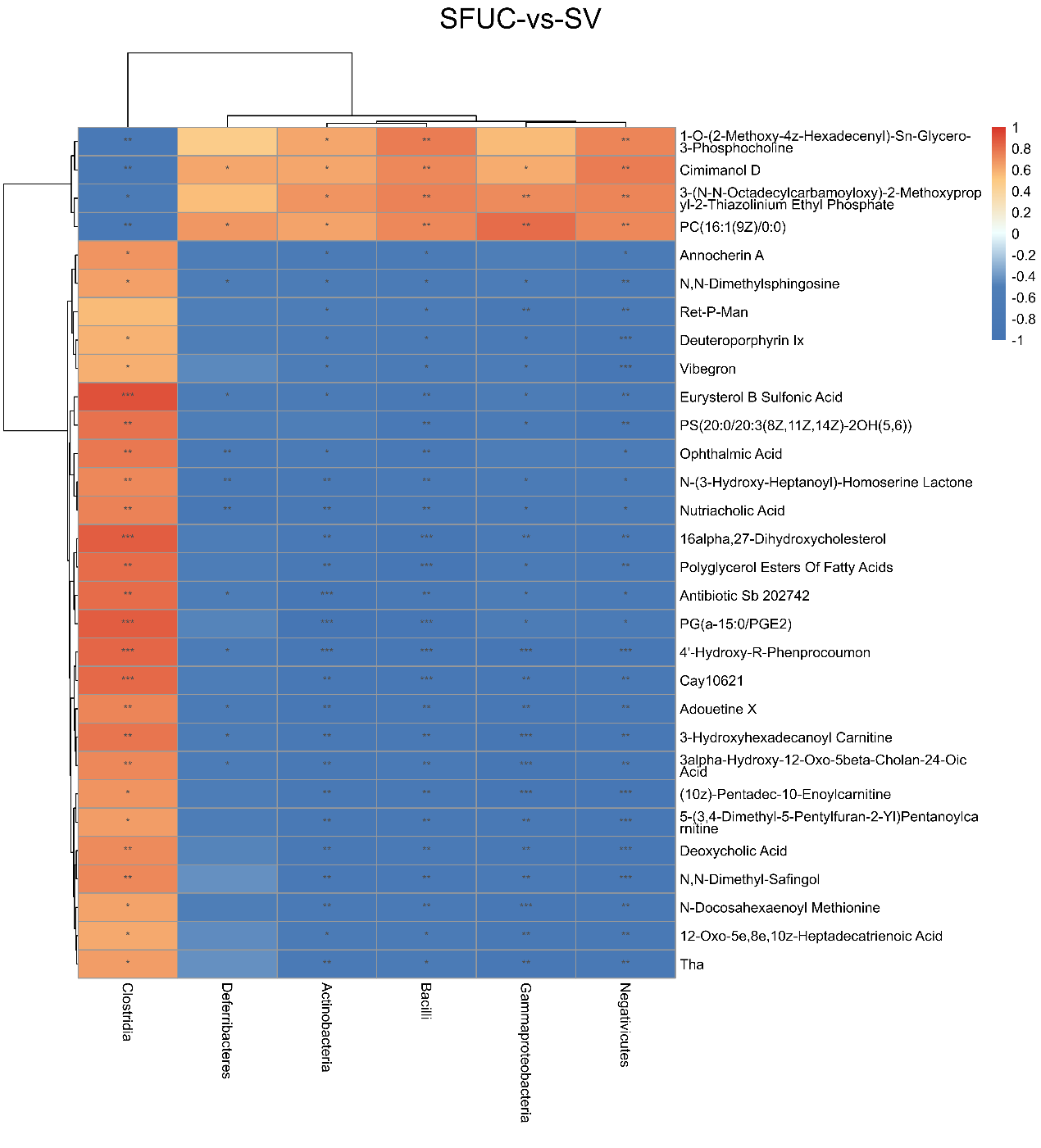
**

**H. SFUC vs. SV at the Class level.**

**
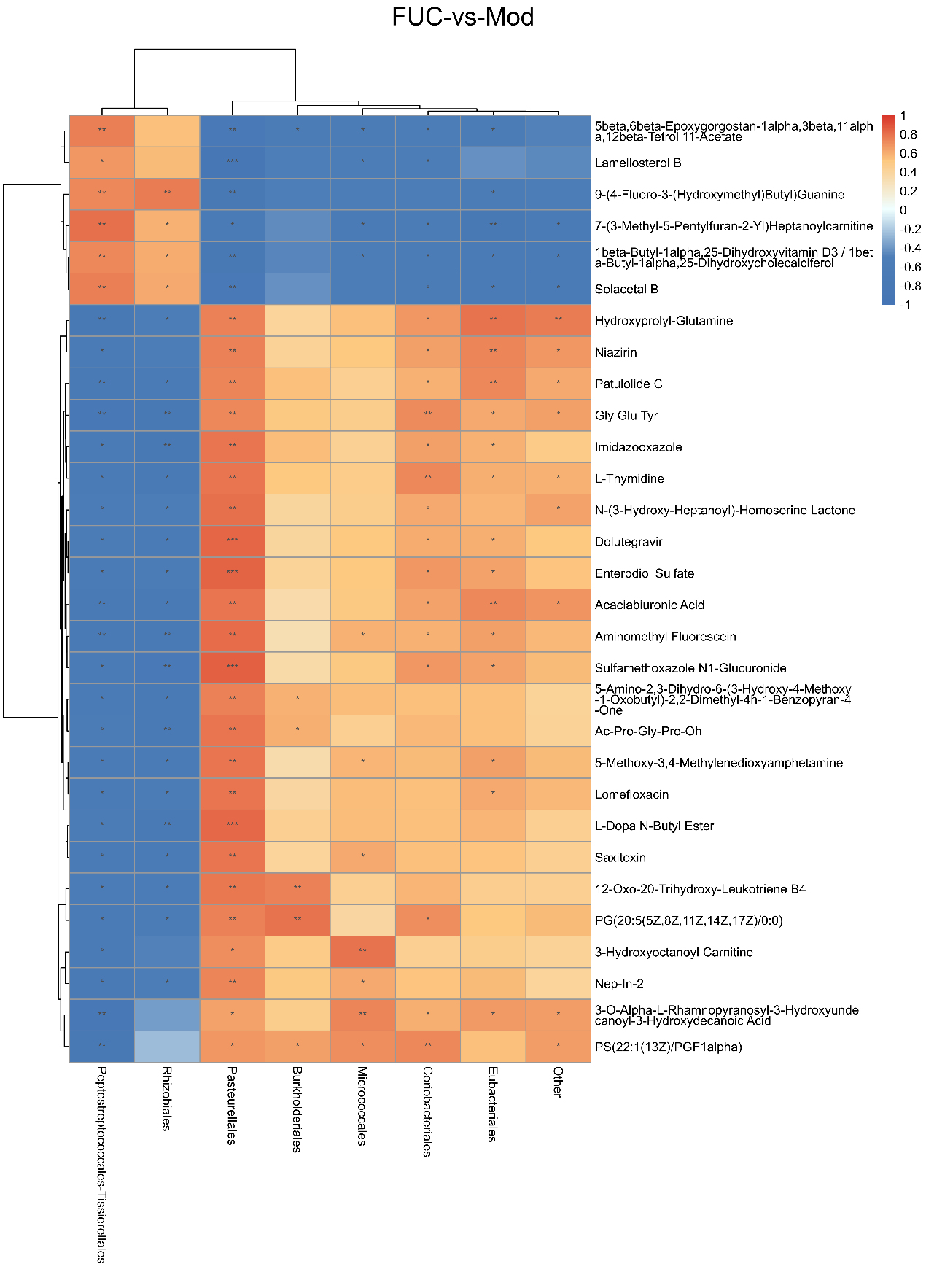
**

**I. Fuc vs. Mod at the Order level.**

**
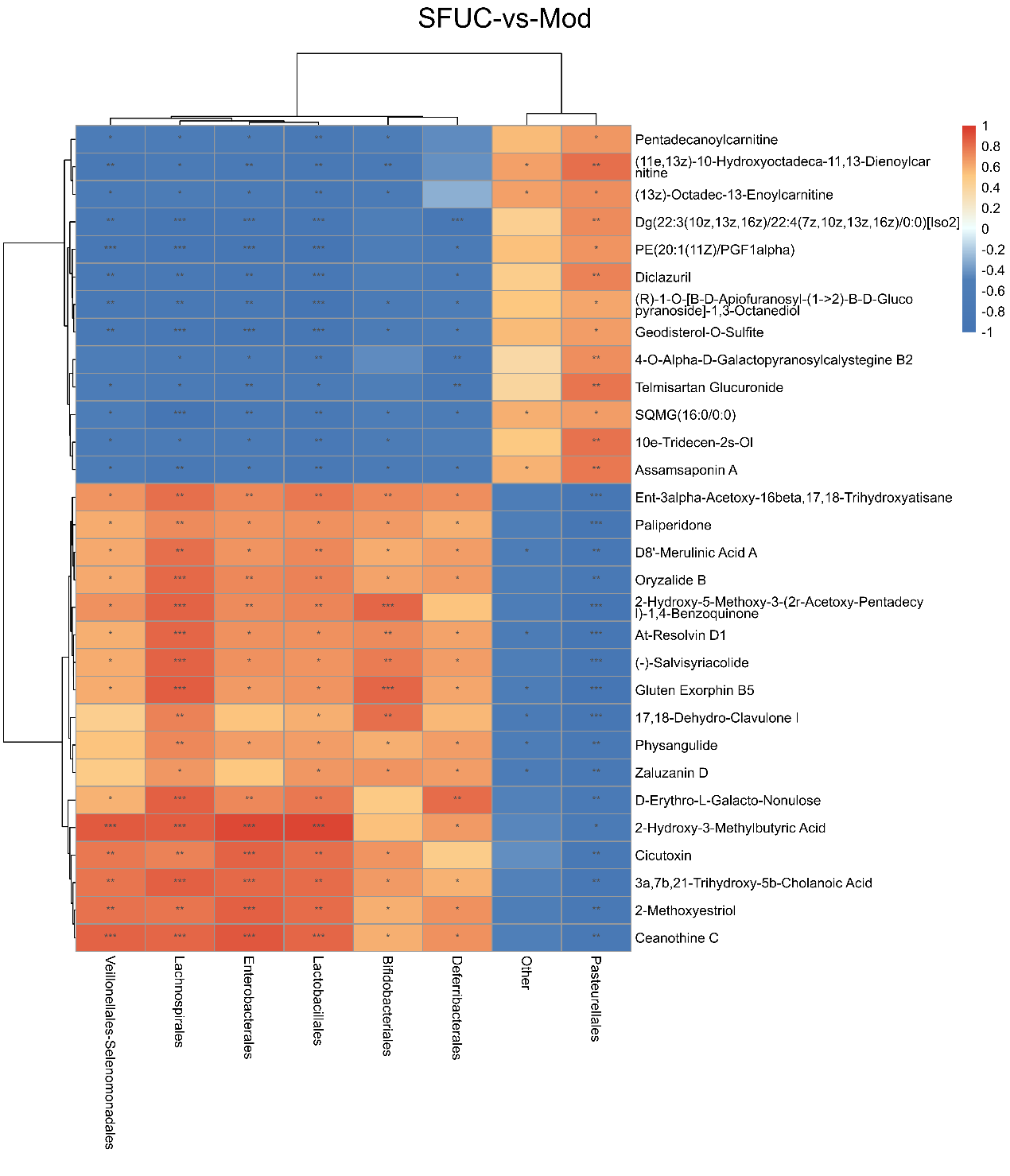
**

**J. SFuc vs. Mod at the Order level.**

**
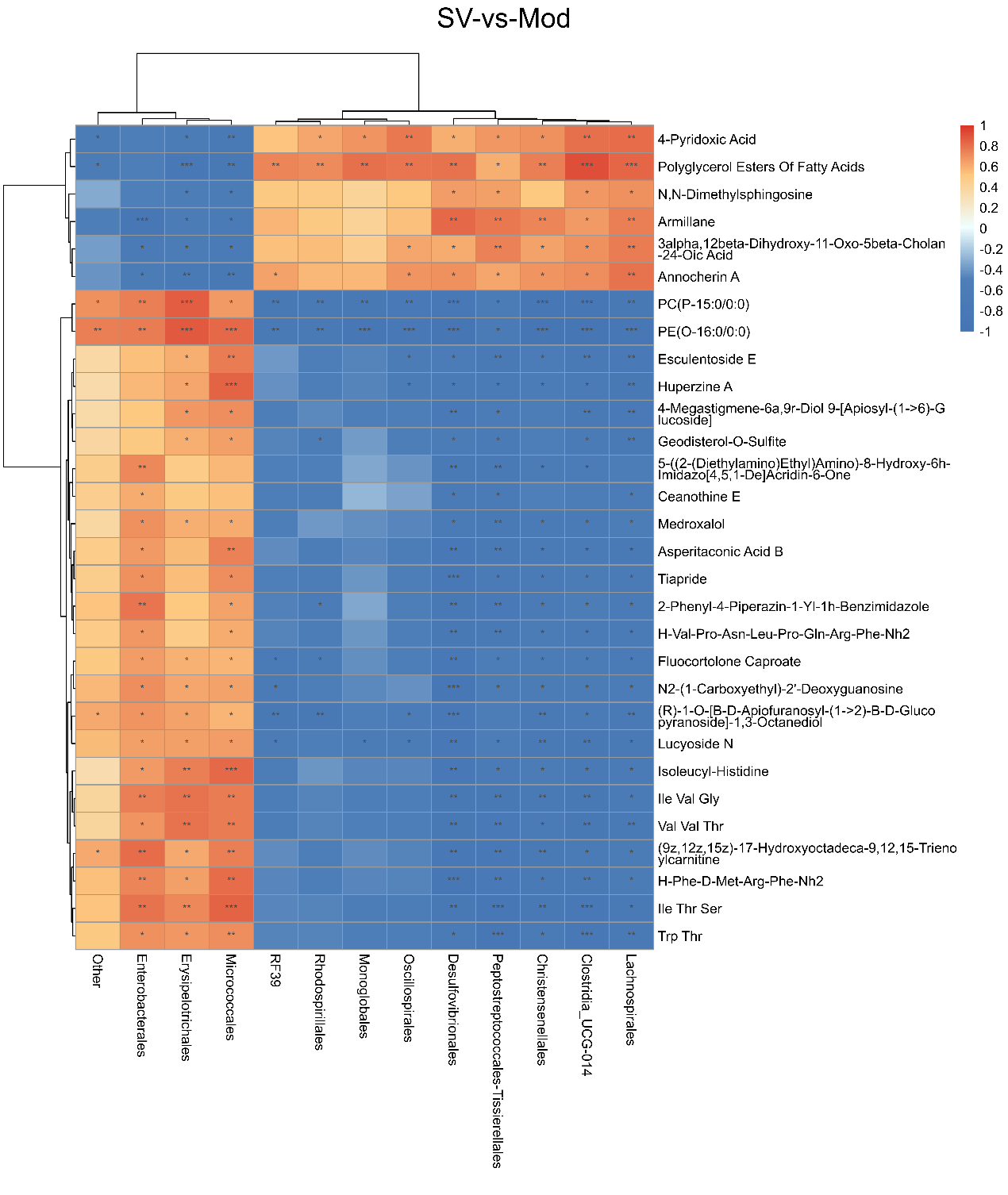
**

**K. SV vs. Mod at the Order level.**

**
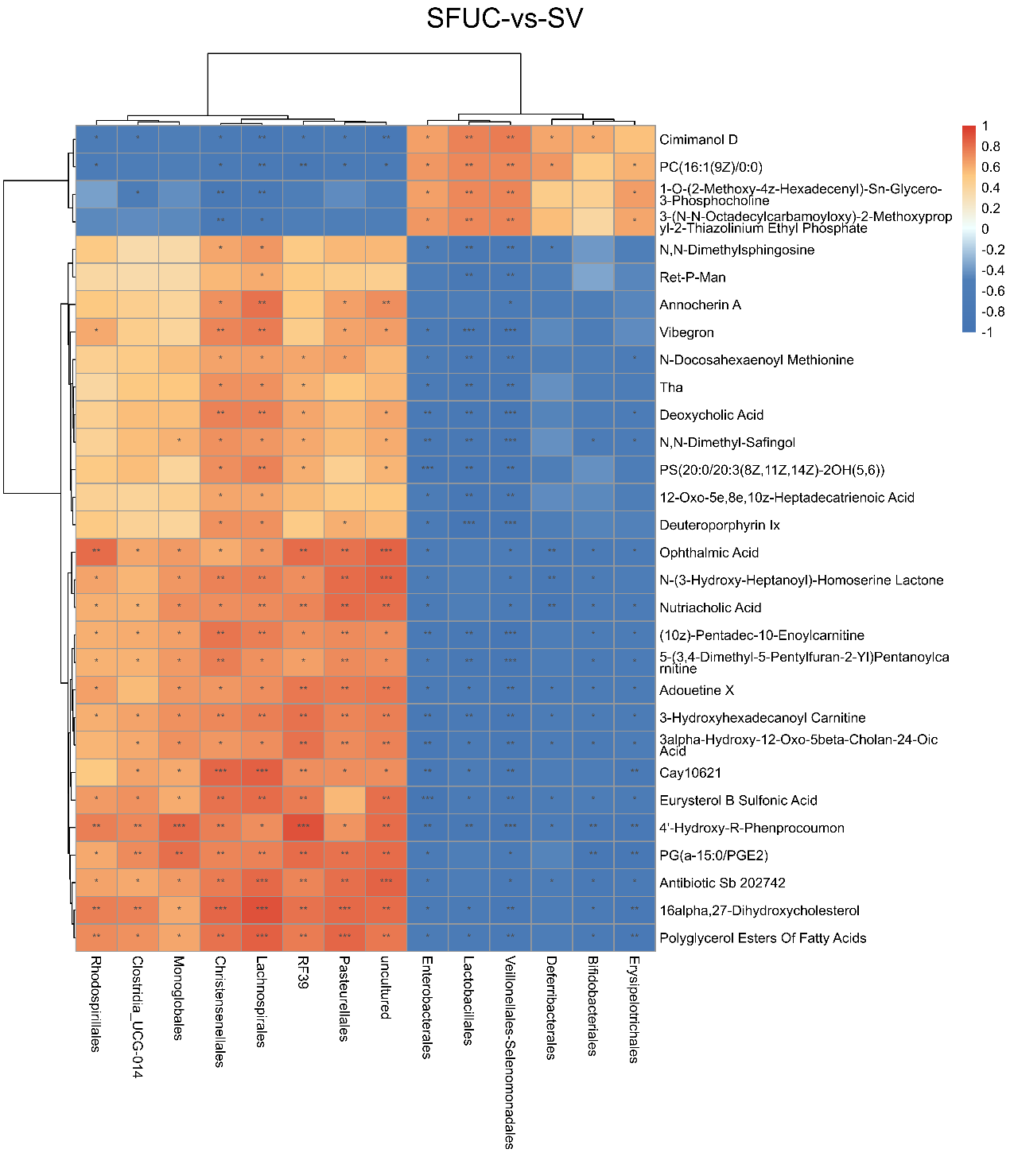
**

**L. SFuc vs. SV at the Order level.**

**
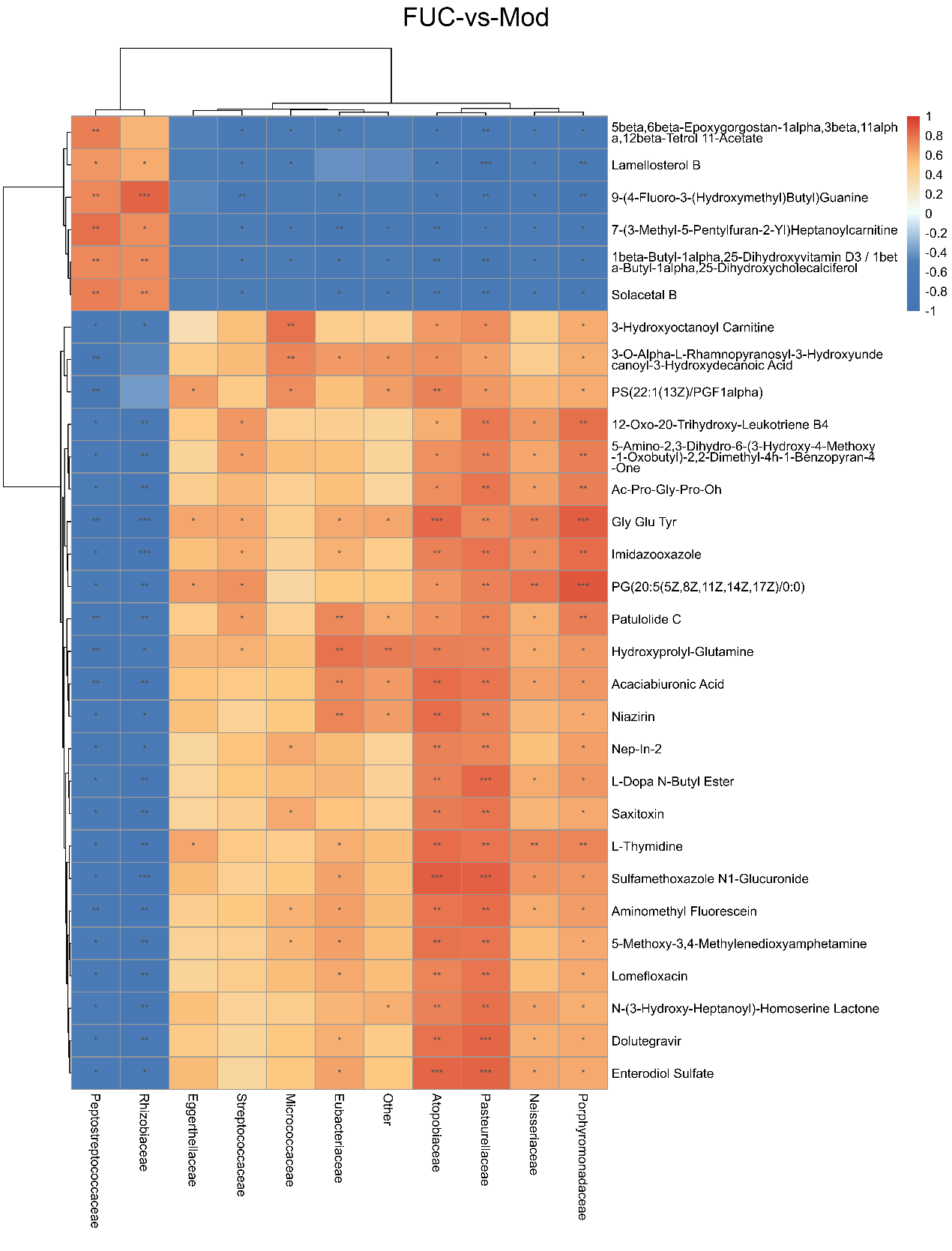
**

**M. Fuc vs. Mod at the Family level.**

**
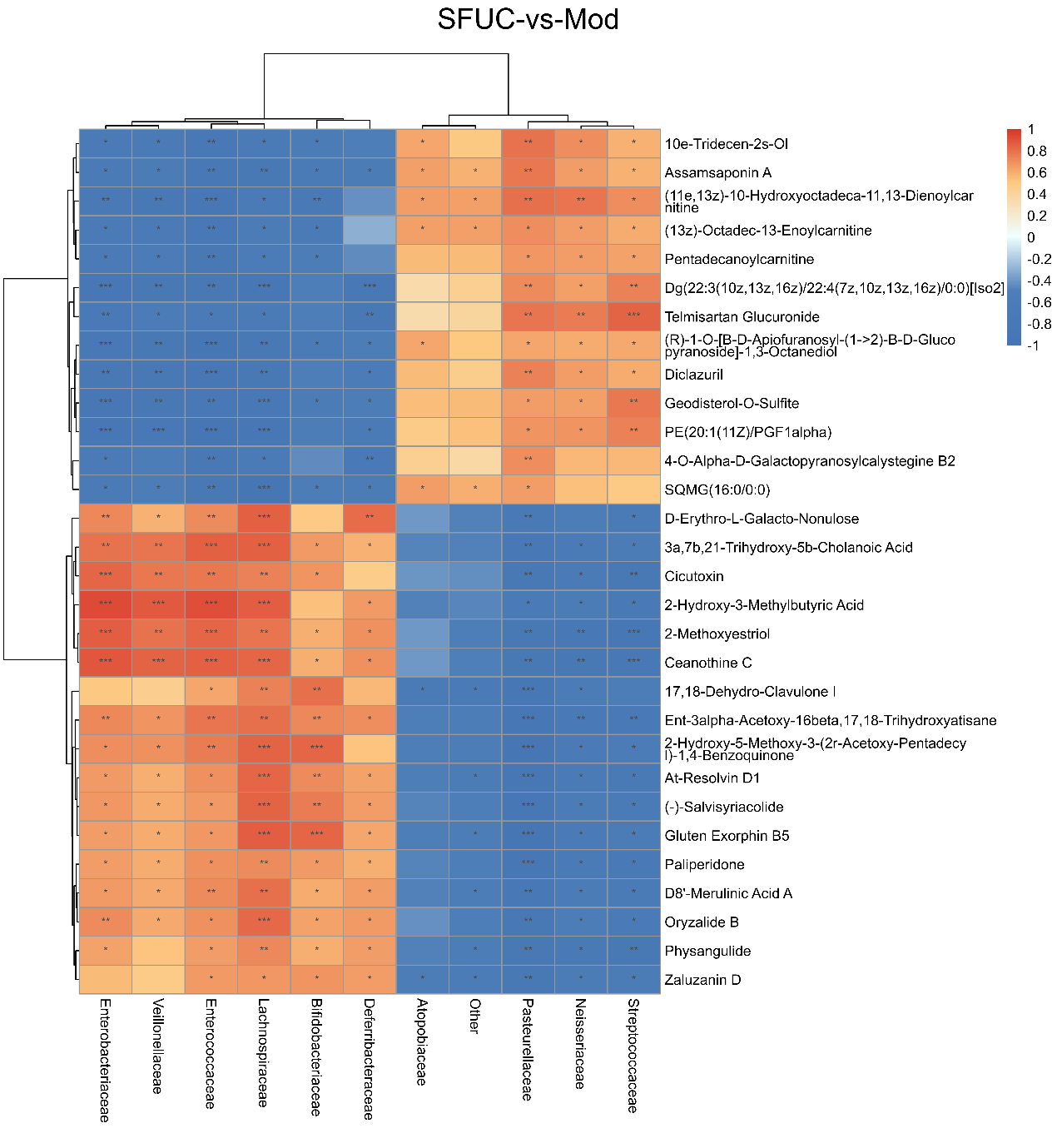
**

**N. SFuc vs. Mod at the Family level.**

**
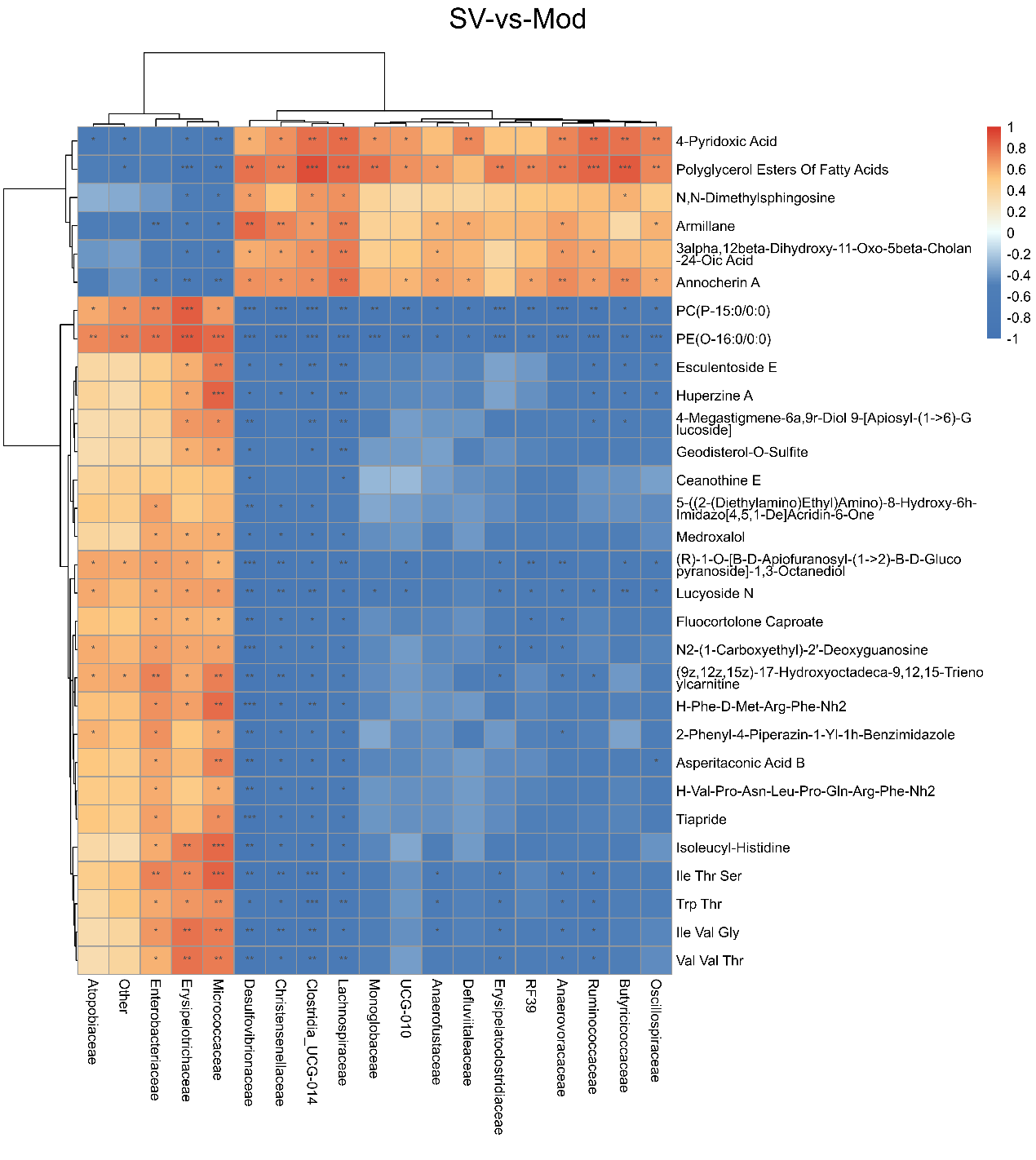
O. SV vs. Mod at the Family level.**

**
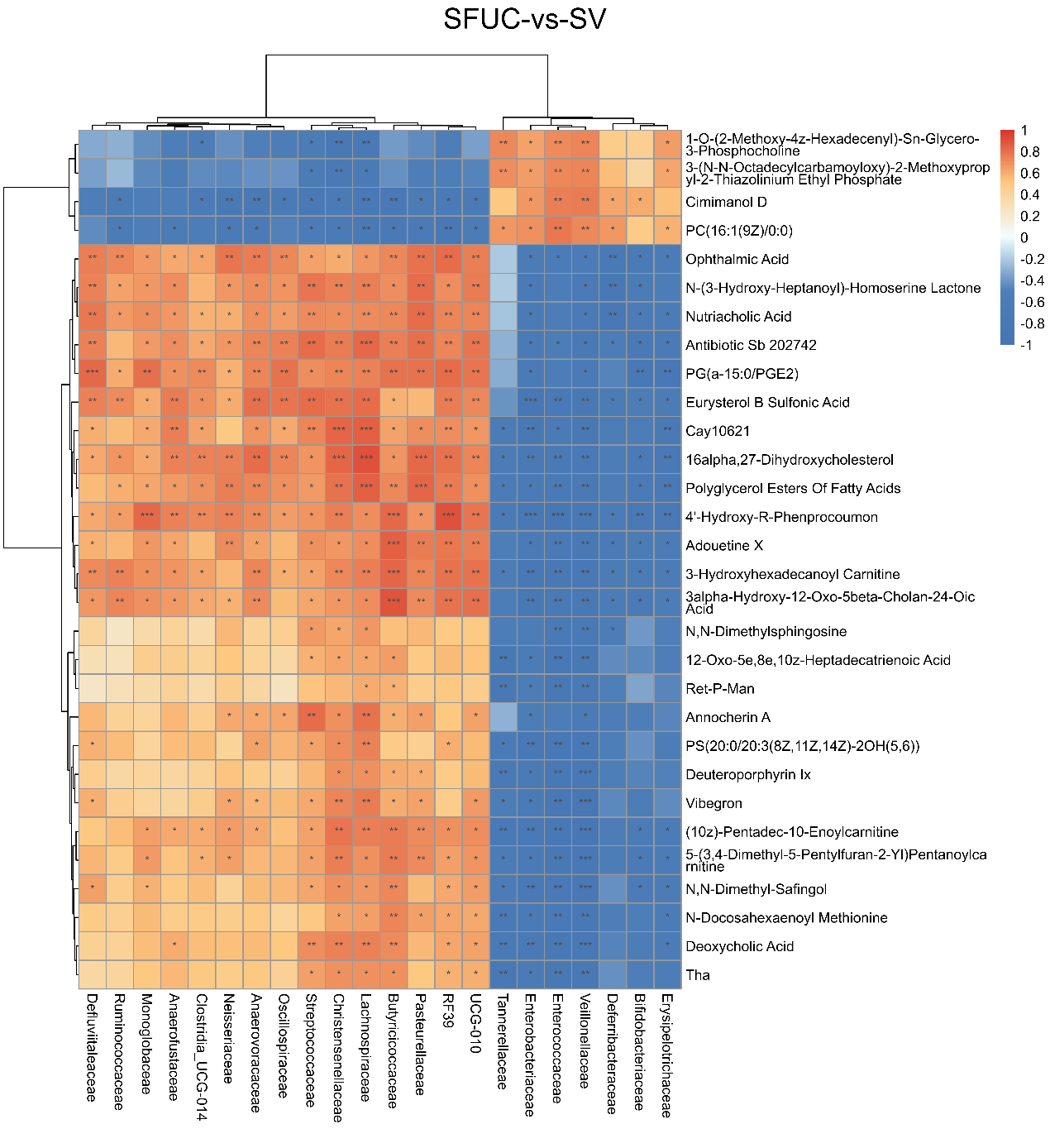
**

**P. SFuc vs. SV at the Family level.**

**
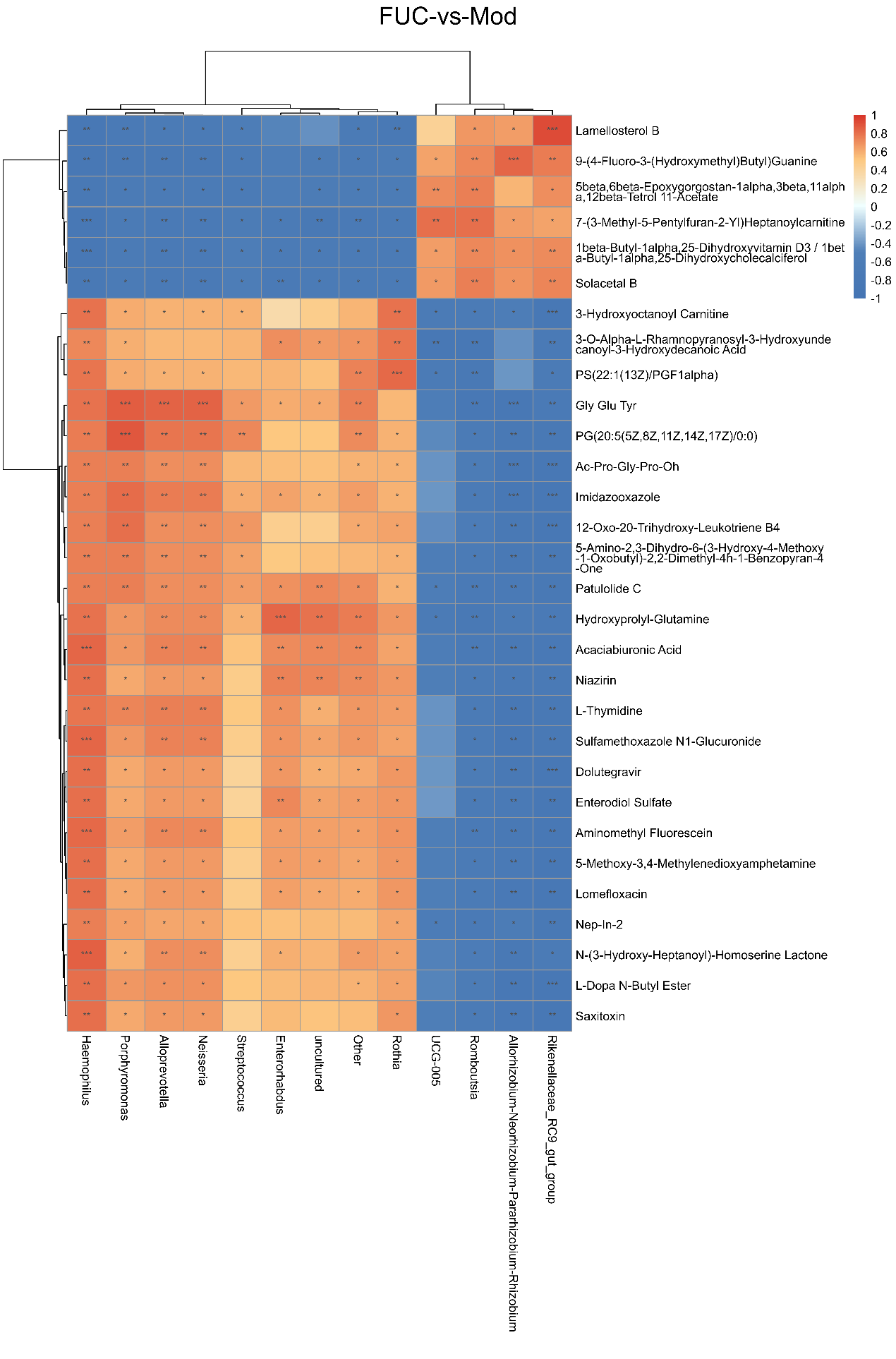
**

**Q. Fuc vs. Mod at the Genes level.** **Note, the abundance of Enterorhabdus was undetectable in more than 5 samples of each group.**

**
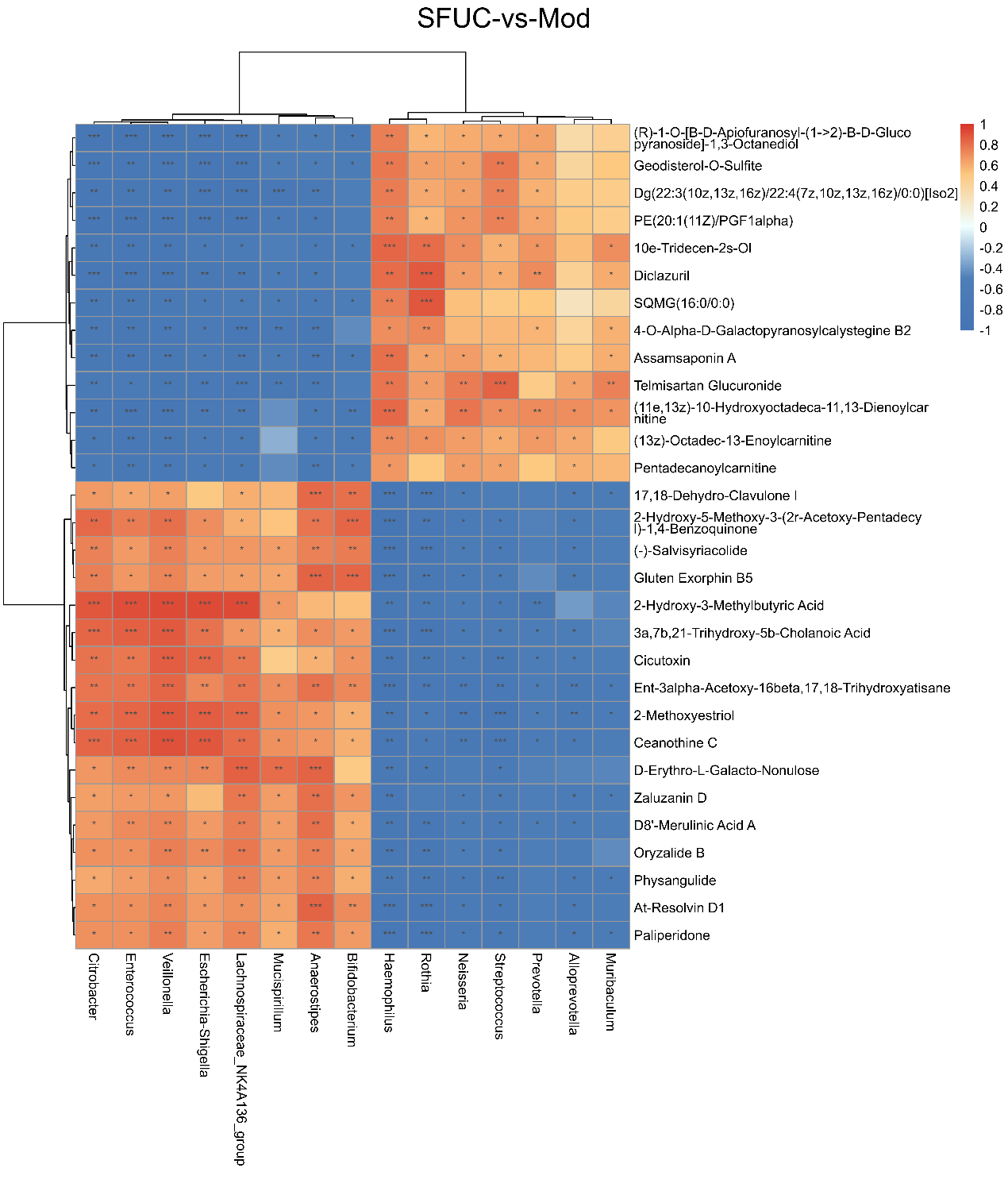
**

**R. SFuc vs. Mod at the Genes level. Note, the abundance of Veillonella was undetectable in more than 5 samples of each group.**

**
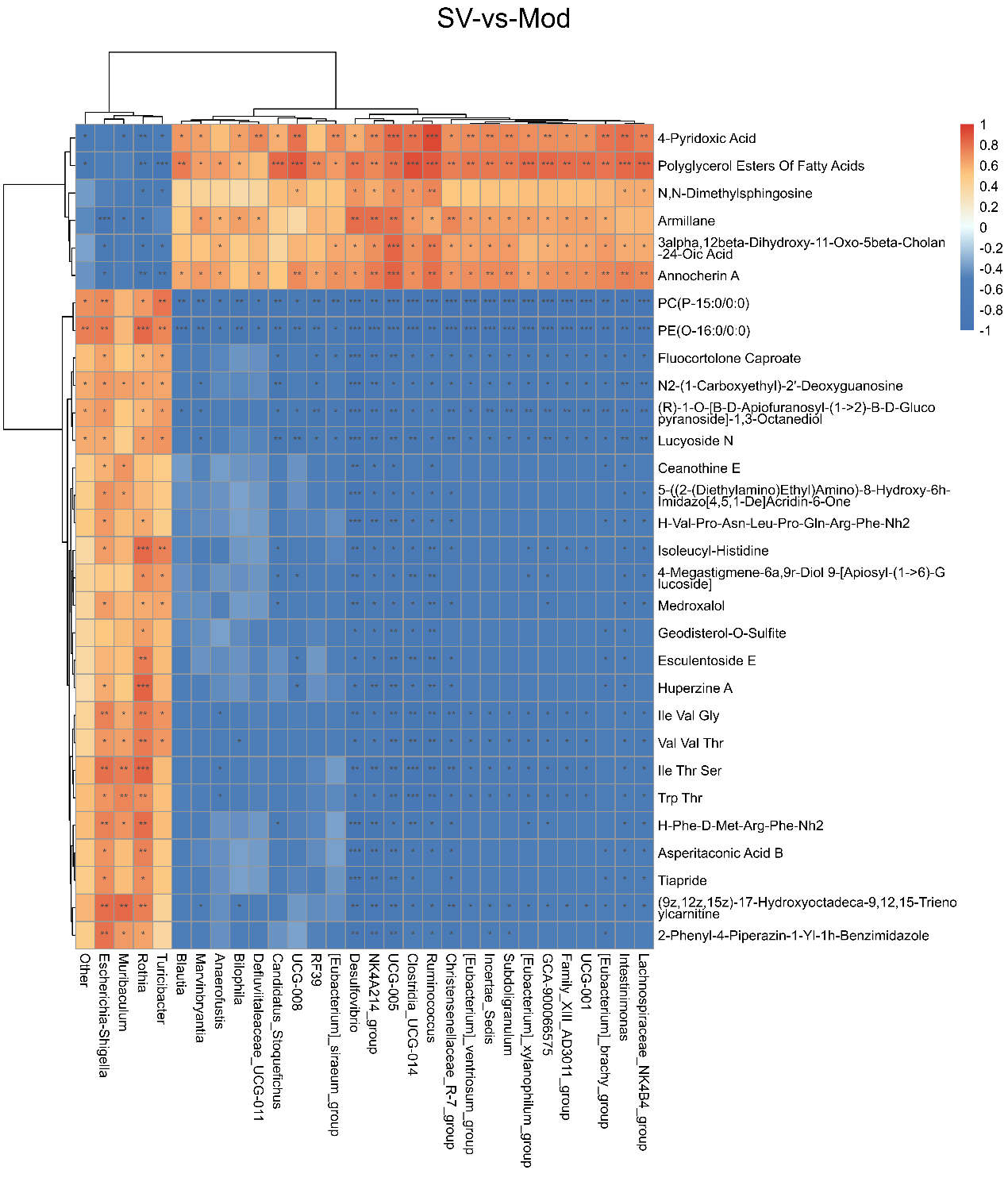
**

**S. SV vs. Mod at the Genes level.**

**
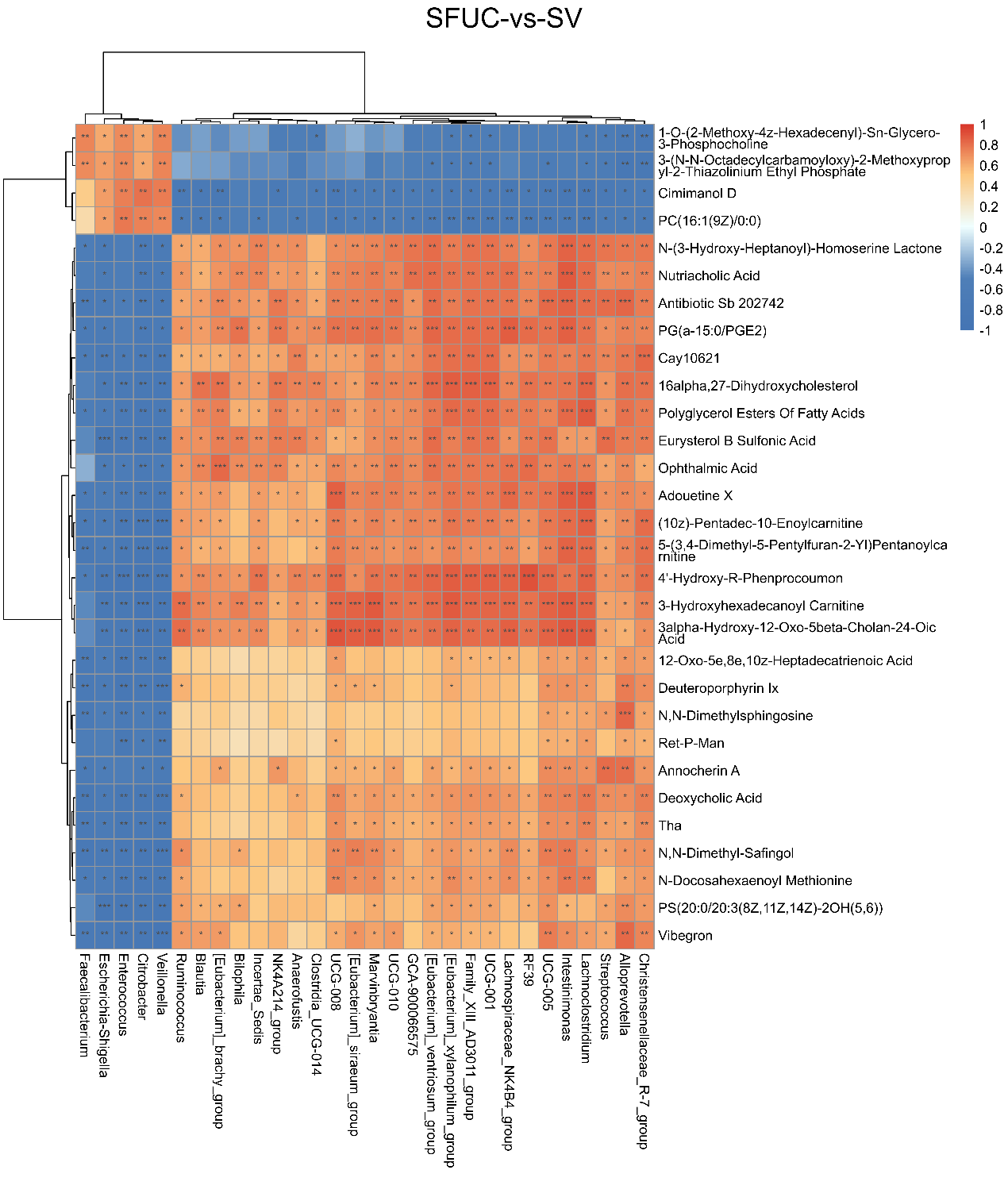
**

**T. SFuc vs. SV at the Genes level.**

**
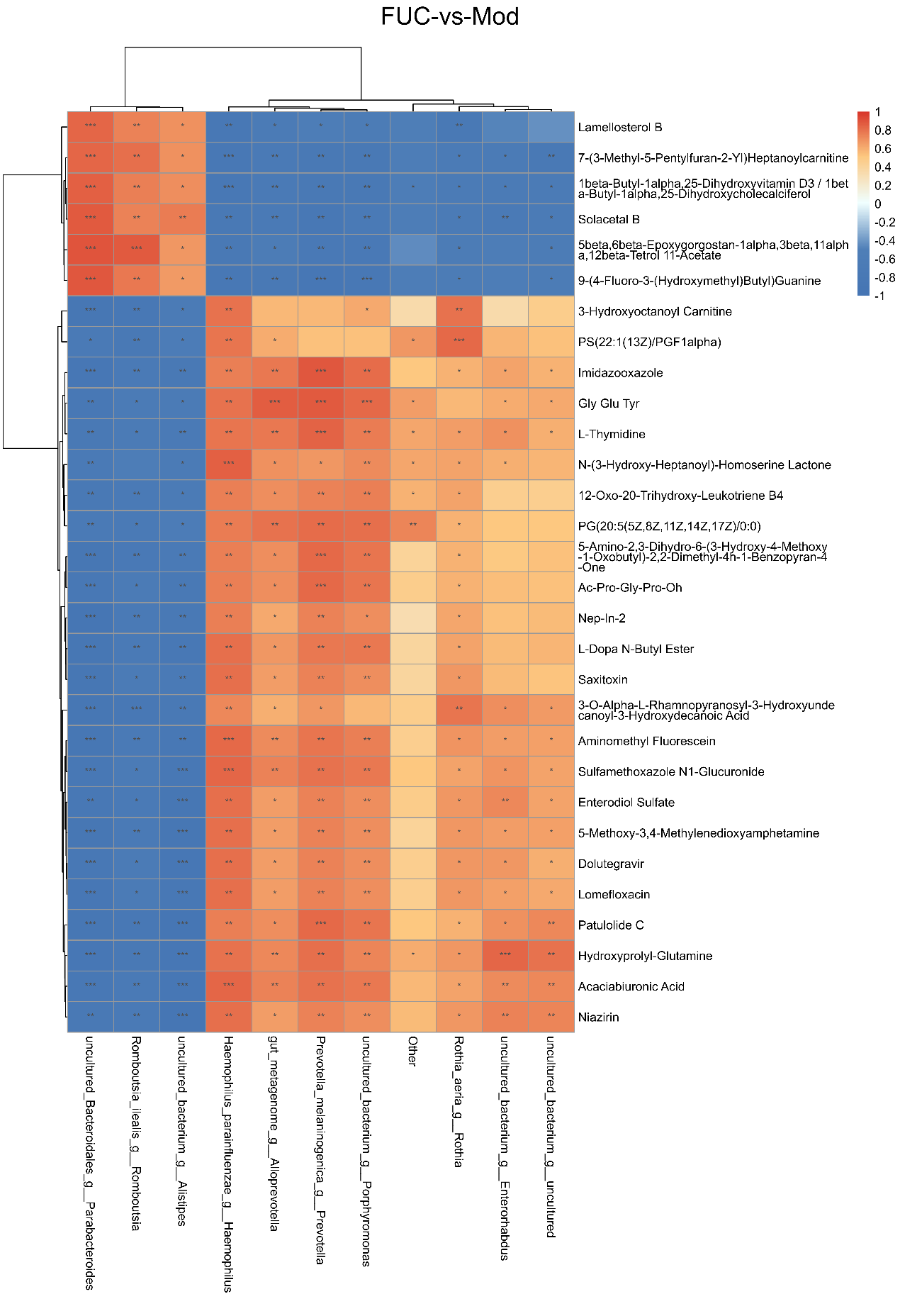
**

**U. Fuc vs. Mod at the Species level.**

**
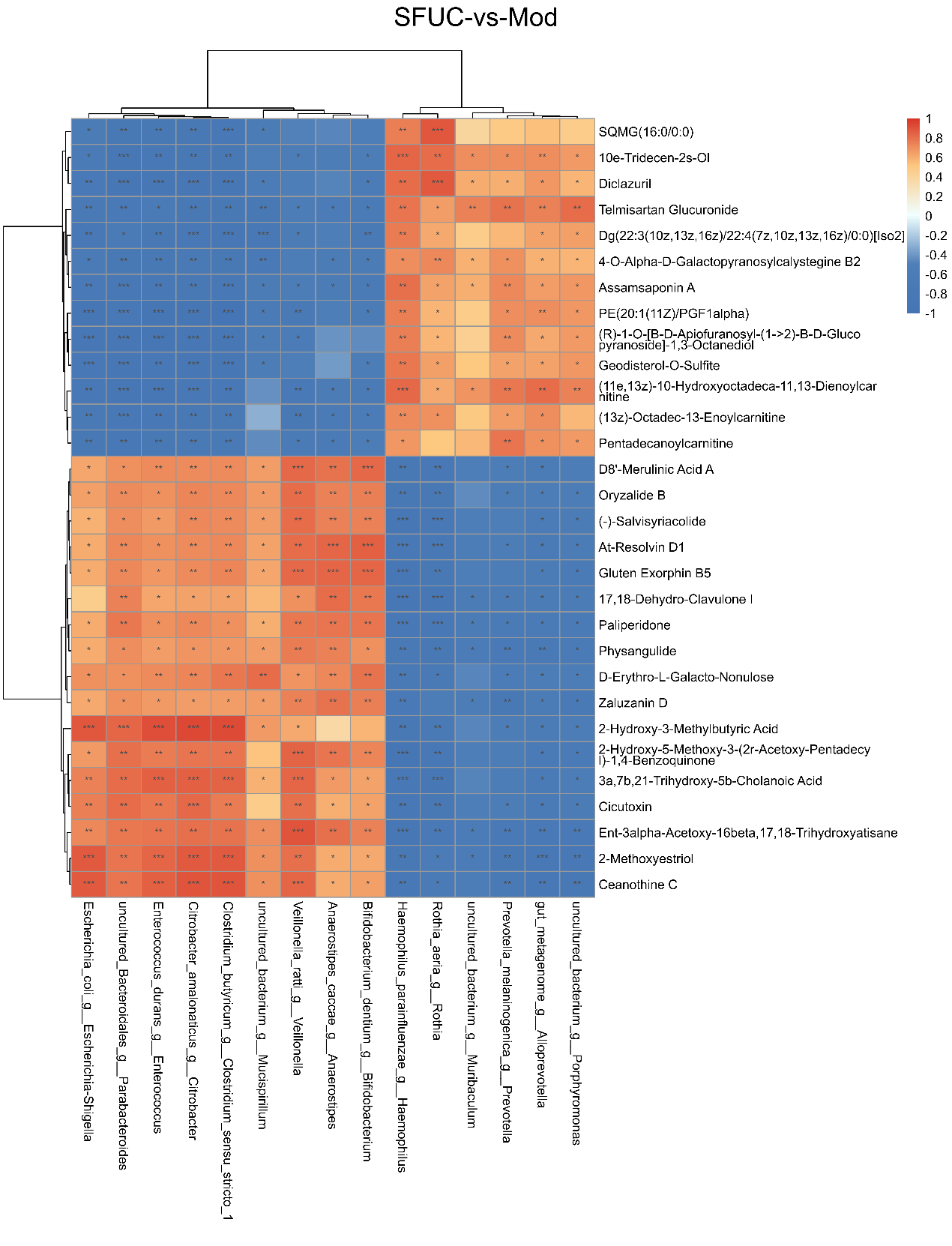
**

**V. SFuc vs. Mod at the Species level.**

**
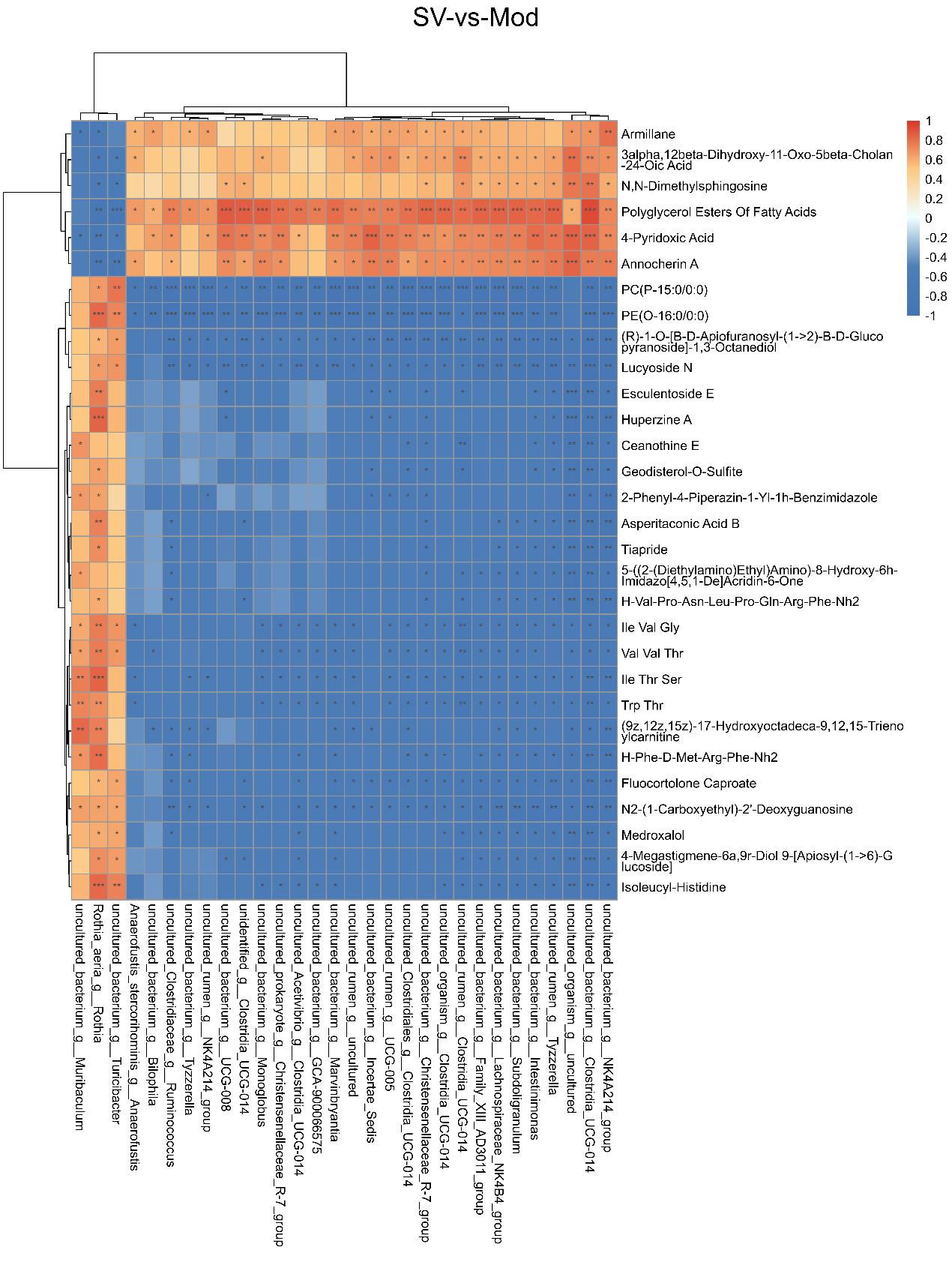
**

**W. SV vs. Mod at the Species level.**

**
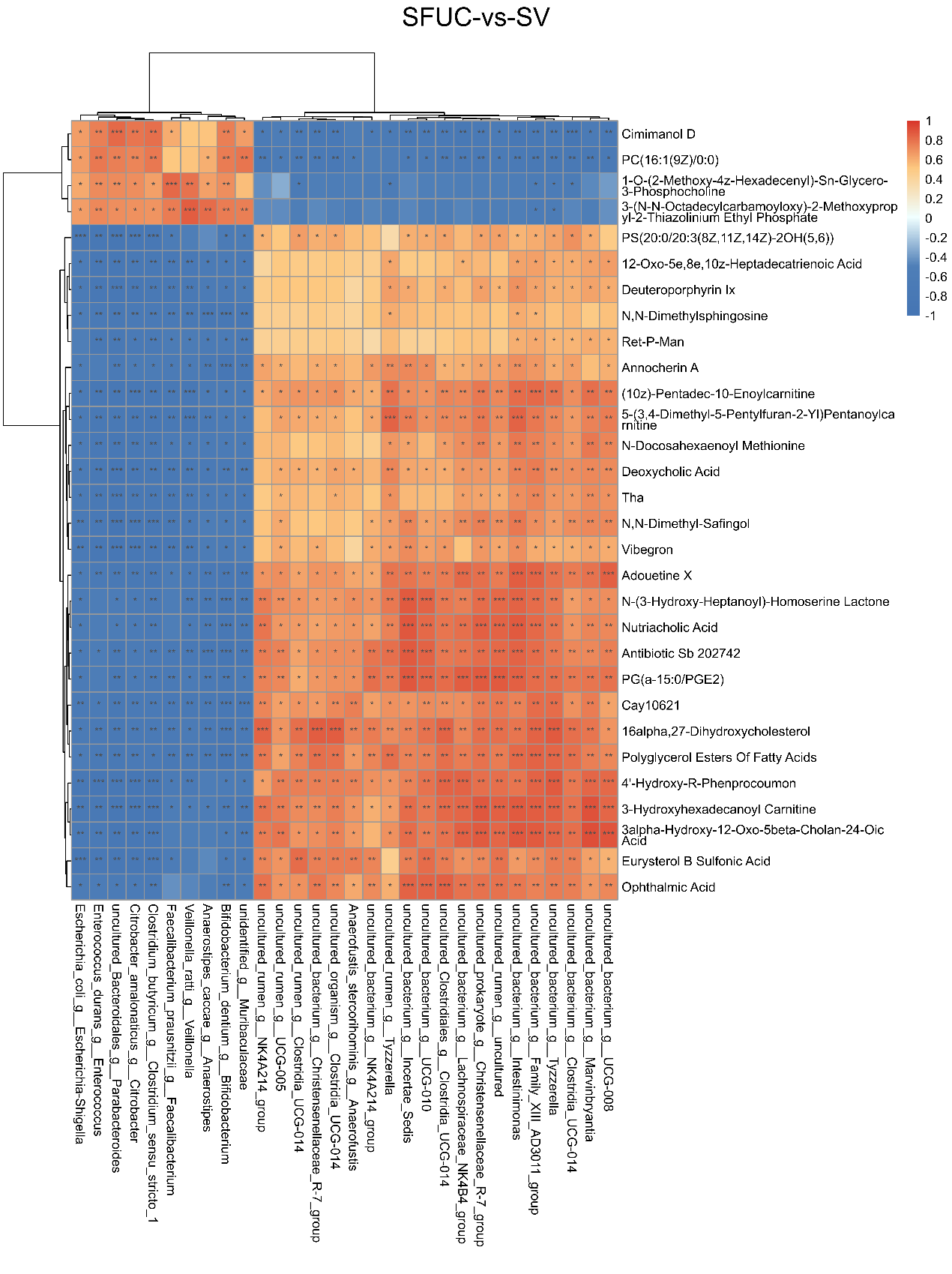
**

**X. SFuc vs. SV at the Species level.**

**Supplementary Figure 4.** Correlation analysis between significantly regulated gut microbiota and metabolites at different levels**.**
